# Plasma metabolites and risk of seven cancers: a two-sample Mendelian randomization study among European descendants

**DOI:** 10.1186/s12916-024-03272-8

**Published:** 2024-03-04

**Authors:** Yaxin Chen, Yufang Xie, Hang Ci, Zhengpei Cheng, Yongjie Kuang, Shuqing Li, Gang Wang, Yawen Qi, Jun Tang, Dan Liu, Weimin Li, Yaohua Yang

**Affiliations:** 1 Institute of Respiratory Health, Frontiers Science Center for Disease-Related Molecular Network, West China Hospital, Sichuan University, Guoxue Alley 37, Chengdu, Sichuan China; 2 Department of Respiratory and Critical Care Medicine, West China Hospital, Sichuan University, Chengdu, Sichuan China; 3 Center for Public Health Genomics, Department of Public Health Sciences, UVA Comprehensive Cancer Center, School of Medicine, University of Virginia, 560 Ray C. Hunt Dr., Rm 4408, Charlottesville, VA USA; 4 Department of Public Health Sciences, UVA Comprehensive Cancer Center, School of Medicine, University of Virginia, Charlottesville, VA USA; 5 Innovation Laboratory for Precision Diagnostics, Precision Medicine Research Center, Precision Medicine Key Laboratory of Sichuan Province, West China Hospital, Sichuan University, Chengdu, Sichuan China

**Keywords:** Plasma metabolites, Cancer risk, Mendelian randomization

## Abstract

**Background:**

While circulating metabolites have been increasingly linked to cancer risk, the causality underlying these associations remains largely uninterrogated.

**Methods:**

We conducted a comprehensive 2-sample Mendelian randomization (MR) study to evaluate the potential causal relationship between 913 plasma metabolites and the risk of seven cancers among European-ancestry individuals. Data on variant-metabolite associations were obtained from a genome-wide association study (GWAS) of plasma metabolites among 14,296 subjects. Data on variant-cancer associations were gathered from large-scale GWAS consortia for breast (*N* = 266,081), colorectal (*N* = 185,616), lung (*N* = 85,716), ovarian (*N* = 63,347), prostate (*N* = 140,306), renal cell (*N* = 31,190), and testicular germ cell (*N* = 28,135) cancers. MR analyses were performed with the inverse variance-weighted (IVW) method as the primary strategy to identify significant associations at Bonferroni-corrected *P* < 0.05 for each cancer type separately. Significant associations were subjected to additional scrutiny via weighted median MR, Egger regression, MR-Pleiotropy RESidual Sum and Outlier (MR-PRESSO), and reverse MR analyses. Replication analyses were performed using an independent dataset from a plasma metabolite GWAS including 8,129 participants of European ancestry.

**Results:**

We identified 94 significant associations, suggesting putative causal associations between 66 distinct plasma metabolites and the risk of seven cancers. Remarkably, 68.2% (45) of these metabolites were each associated with the risk of a specific cancer. Among the 66 metabolites, O-methylcatechol sulfate and 4-vinylphenol sulfate demonstrated the most pronounced positive and negative associations with cancer risk, respectively. Genetically proxied plasma levels of these two metabolites were significantly associated with the risk of lung cancer and renal cell cancer, with an odds ratio and 95% confidence interval of 2.81 (2.33–3.37) and 0.49 (0.40–0.61), respectively. None of these 94 associations was biased by weak instruments, horizontal pleiotropy, or reverse causation. Further, 64 of these 94 were eligible for replication analyses, and 54 (84.4%) showed *P* < 0.05 with association patterns consistent with those shown in primary analyses.

**Conclusions:**

Our study unveils plausible causal relationships between 66 plasma metabolites and cancer risk, expanding our understanding of the role of circulating metabolites in cancer genetics and etiology. These findings hold promise for enhancing cancer risk assessment and prevention strategies, meriting further exploration.

**Supplementary Information:**

The online version contains supplementary material available at 10.1186/s12916-024-03272-8.

## Background

Cancer is the second leading cause of human mortality, imposing substantial medical and socioeconomic burdens [1]. Consequently, the prioritization of cancer prevention and screening strategies is of critical importance. Epidemiological studies in recent decades have identified multiple genetic, lifestyle, and environmental factors associated with cancer risk [2, 3]. In particular, genome-wide association studies (GWAS) have identified more than 1000 genetic susceptibility variants for various types of cancer [4–7]. However, the etiology of cancer could not be fully explained by these factors. In addition, the intricate interplay among these factors further complicates the inference of potential causality underlying their associations with cancer risk.

Circulating metabolites are small molecules originating from cells, tissues, and biological fluids, including a variety of compounds such as amino acids, carbohydrates, lipids, and xenobiotics. These molecules have been frequently employed to investigate physiological and pathophysiological processes [8, 9]. Recent observational studies have illuminated metabolic dysregulation as a hallmark of cancer, with multiple circulating metabolites linked to cancer development [10]. For example, elevated plasma pseudouridine was reported to be associated with an increased risk of ovarian cancer [11]. In addition, aberrant L-tryptophan metabolism was shown to drive the progression of breast, renal cell, and bladder cancers [12]. Therefore, investigating the metabolites associated with cancer development not only aids in early cancer screening and prevention, but also enhances our insights into the biological mechanisms underlying cancer treatment. However, these studies mainly focused on a small subset of metabolites and were limited by biases commonly encountered in conventional epidemiological studies, such as small sample sizes, potential confounders, and reverse causation.

Various factors influence metabolite levels, including genetics [13]. The advent of untargeted and targeted metabolomics technologies has facilitated the exploration of the genetic architecture of thousands of metabolites [13–17]. Typically, these investigations measure metabolite abundance in the blood, effectively reflecting the aggregative metabolite concentrations across tissues [18]. Notably, a recent study performed genotyping and untargeted plasma metabolomic profiling among 19,994 subjects of European ancestry and identified 2599 significant associations between genetic variants and metabolites [17]. Intriguingly, a considerable proportion of these variants were found to colocalize with GWAS-identified risk variants for various diseases, including cancer [17]. The shared genetic determinant over both plasma metabolites and cancer forms a strong basis for the exploration of the relationship between them using genetic variants as instrumental variables through Mendelian randomization (MR) studies. Given the random allocation of alleles during gamete formation, findings from MR analyses hold the potential to infer causal connections between exposures and outcomes [19, 20].

Several MR studies have revealed circulating metabolites with genetically predicted levels that might be causally associated with cancer risk. For example, docosapentaenoic acid [21] and high-density lipoprotein [22] were found to be associated with increased lung cancer and breast cancer risk, respectively. In additional, 1-linoleoylglycerophosphoethanolamine was associated with a reduced risk of colorectal cancer [23]. Although these results showcase the potential of circulating metabolites as causal biomarkers for cancer, it is important to note that most of these studies only investigated a limited number of metabolites, primarily owing to the relatively slow adoption of untargeted metabolomics platforms. On the other hand, a majority of these studies did not take full advantage of the most up-to-date GWAS data for both metabolites and cancers. This potential oversight could have resulted in the utilization of weak genetic instruments and less precise effect size estimates for both variant-metabolite and variant-cancer associations.

To address these limitations, we meticulously assembled the most comprehensive GWAS data available to date for untargeted metabolomics and seven cancers among individuals of European descent. Leveraging these datasets, we conducted a two-sample MR study to unravel the potential causal relationship between 913 plasma metabolites and the risk of breast, lung, colorectal, prostate, ovarian, renal cell, and testicular germ cell cancers. For the significant causal associations we identified, a series of complementary analyses were conducted to reinforce their reliability and robustness.

## Methods

### Study design

**Fig. 1:**
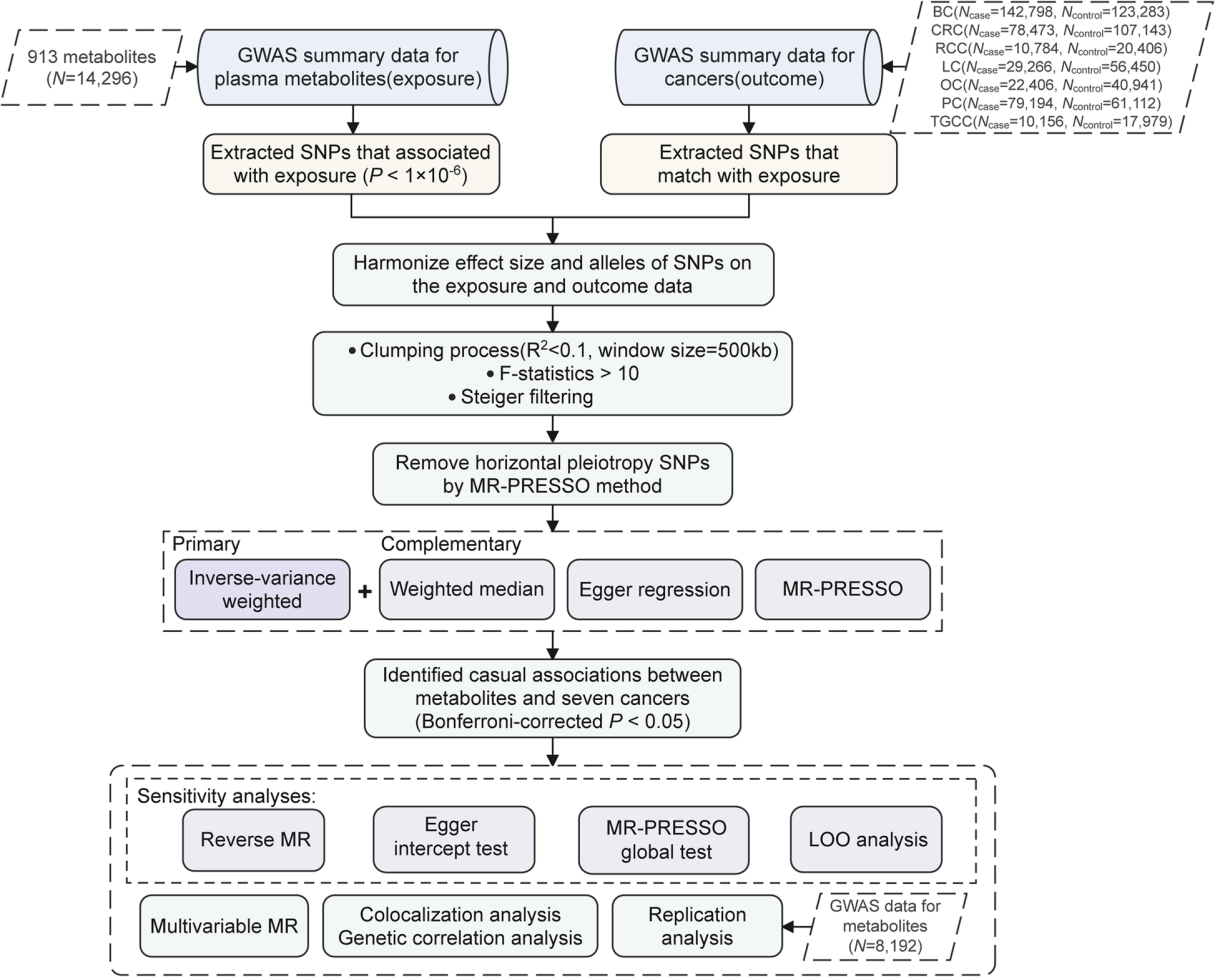
Overall study design and workflow. GWAS, genome-wide association study; BC, breast cancer; CRC, colorectal cancer; RCC, renal cell cancer; LC, lung cancer; OC, ovarian cancer; PC, prostate cancer; TGCC, testicular germ cell cancer; SNPs, single nucleotide polymorphisms; kb, kilobase; MR-PRESSO, Mendelian Randomization Pleiotropy RESidual Sum and Outlier; LOO, leave-one-out

The overall study workflow is illustrated in Fig. 1. In this study, we treated the plasma level of each metabolite as the exposure, and the risk of each cancer as the outcome. Single nucleotide polymorphisms (SNPs) significantly associated with exposure were utilized as instrumental variables (IVs). For a robust MR study, each IV should be significantly associated with the exposure, independent of all the other IVs and potential confounding factors, and impacting the outcome only by influencing the exposure [19, 20]. These principles were carefully adhered to throughout the entire study. We conducted a comprehensive set of downstream analyses to account for potential biases that might undermine the reliability of our findings. Specifically, we estimated *F*-statistics and conducted the Steiger test to ensure the validity of IVs, employed Egger regression and Mendelian Randomization Pleiotropy RESidual Sum and Outlier (MR-PRESSO) to detect and correct for horizontal pleiotropy and outliers, conducted leave-one-out (LOO) analyses to evaluate the presences of predominant IVs, and performed reverse MR analyses to determine the possibility of reverse causation [24–26]. We additionally implemented genetic correlation analyses to assess if shared genetic factors between metabolites and cancer risk might have confounded our MR estimates. Colocalization analyses were also conducted to examine the presence of shared causal variants between metabolites and cancer risk in genomic loci where IVs reside. For metabolites showing significant associations with the risk of each specific cancer type, we further performed multivariable MR (MVMR) analyses [27] to nominate metabolites that might directly influence cancer risk independent of the effects of all the other metabolites. Finally, an independent plasma metabolites GWAS dataset was employed to evaluate the findings from our primary analyses.

### Exposure data

GWAS data of plasma metabolites were sourced from an interactive web server accessible at https://omicscience.org/apps/mgwas/mgwas.table.php. This dataset comprised a total of 913 metabolites quantified for 14,296 individuals of European descent from the EPIC-Norfolk study and the INTERVAL study [17]. In brief, untargeted plasma metabolomic profiling was conducted using the Metabolon HD4 platform. Genotyping was performed using the Affymetrix Axiom Array, and data were imputed with the 1000 Genomes Phase 3-UK10K data as the reference panel. GWAS analyses were conducted within each cohort via linear regression analyses adjusting for age and sex, and the results were combined through inverse variance-weighted fixed-effect meta-analyses [17].

To externally validate the findings based on data from the EPIC-Norfolk and INTERVAL study, we utilized data from an independent GWAS of 1091 blood metabolites among 8192 individuals of European ancestry from the Canadian Longitudinal Study on Aging (CLSA) [14]. Summary-level GWAS statistics for these 1091 metabolites were retrieved from the GWAS catalog, under the accession numbers GCST90199621-GCST90201020.

### Outcome data

Summary statistics data for GWAS on seven distinct cancers among European ancestry subjects were collected from large-scale GWAS consortia. Detailed information of these data and consortia is presented in Additional file 1: Table S1. Briefly, breast cancer data were obtained from the Breast Cancer Association Consortium (BCAC), including 142,798 cases and 123,283 controls [28]. Data on colorectal cancer were sourced from the GWAS catalog (GCST90255675), including 78,473 cases and 107,143 controls [29] from the Colorectal Cancer Transdisciplinary Study (CORECT), the Genetics and Epidemiology of Colorectal Cancer Consortium (GECCO), the Colon Cancer Family Registry (CCFR), and the UK Biobank. Data on lung cancer were obtained from the GWAS catalog (GCST004748), including 29,266 cases and 56,450 controls from the Lung Cancer Cohort Consortium (LC3) and the Transdisciplinary Research of Cancer in Lung of the International Lung Cancer Consortium (TRICL-ILCCO) [6]. Data on renal cell cancer were acquired from the database of Genotypes and Phenotypes (dbGaP; phs001736.v2.p1), including 10,784 cases and 20,406 controls from the International Agency for Research on Cancer (IARC), the National Cancer Institute (NCI), the University of Texas MD Anderson Cancer, and the Institute of Cancer Research, UK [30]. Data of prostate cancer was accessed from the Prostate Cancer Association Group to Investigate Cancer Associated Alterations in the Genome (PRACTICAL) consortium, including 79,194 cases and 61,112 controls [4]. Data on ovarian cancer were obtained from the GWAS catalog under GCST004415, including 22,406 cases and 40,941 controls from the Ovarian Cancer Association Consortium (OCAC) [31]. Data on testicular germ cell cancer were retrieved from dbGaP (phs001349.v2.p1), including 10,156 cases and 17,979 controls from the Testicular Cancer Consortium (TCC) [7].

### Selection of genetic IVs

For each metabolite, non-palindromic SNPs with a minor allele frequency (MAF) of > 0.05 in the 1000 Genome Project (phase 3 version 5 focusing on European descendants) and shown in cancer GWAS data were used for IV selection. Linkage disequilibrium (LD) clumping was performed with a window size of 500 kilobase (kb) to select SNPs that were independently (pairwise LD *r*^2^ < 0.1) associated with plasma metabolites at *P* < 1 × 10^−6^, as previously described [23, 32, 33]. For each metabolite, the variance in its plasma level explained by each IV (*R*^2^) and the strength of each IV (*F*-statistics) were calculated using formulas ${R}^{2}=(2{\beta }^{2}\times {\text{EAF}}\times (1-{\text{EAF}}))/(2{\beta }^{2}\times {\text{EAF}}\times (1-EAF) +2N\times {\text{EAF}}\times (1-{\text{EAF}}) \times {{\text{SE}}}^{2})$ and $F=({R}^{2}\times (N-2))/(1-{R}^{2})$, respectively. In these formulas, ${\text{EAF}}$ denotes the effect allele frequency. $\beta$ and ${\text{SE}}$ represent the effect size and standard error of the SNP-metabolite association, respectively. $N$ is the sample size of the metabolite GWAS [34]. After excluding weak IVs by *F*-statistic < 10 and the Steiger test [32], metabolites with at least three IVs were eligible for MR analyses. We further applied a more stringent threshold, i.e., a window size of 1000 kb, *P* < 5 × 10^−8^, and LD *r*^2^ < 0.001, to select IVs for MR analyses to evaluate the robustness of our findings.

### MR analysis

We employed the inverse variance-weighted (IVW) method as the primary strategy for MR analyses. IVW estimates, known as the assumption of no horizontal pleiotropy across all SNPs, are derived from a comprehensive analysis of Wald ratios for all genetic variants [35]. To account for type I error, Bonferroni correction was applied to analysis results for each cancer type to identify significant associations at Bonferroni-corrected *P* < 0.05. To ensure the robustness of findings, complementary analyses were performed using three additional MR approaches. Specifically, the weighted median [36] method, which assumes that up to half of IVs are invalid, was utilized to address the potential deviations from the strong assumption of IVW that all IVs are valid. Egger regression [26] was applied to identify and adjust for pleiotropic effects, wherein genetic variants influence both the exposure and the outcome. MR-PRESSO [25] was utilized to detect and correct for the impacts of outliers on MR estimates.

### Complementary, sensitivity, and reverse MR analyses

To assess the robustness of significant association identified by IVW, we conducted a series of complementary and sensitivity analyses, including heterogeneity tests to assess the validity of IVs, Egger intercept test and MR-PRESSO global test to evaluate horizontal pleiotropy, and LOO analyses to examine the presence of dominant IVs [37]. To examine the possible reverse causality of the identified significant associations, we performed reverse MR analyses in which cancer was treated as the exposure and metabolites as the exposure. Given the substantially larger sample size of cancer GWAS, more stringent criteria as recommended by previous studies [32] were applied to select SNPs that were independently (pairwise LD *r*^2^ < 0.001 in 1000 kb window) associated with cancer at the genome-wide statistical significance level of *P* < 5 × 10^−8^ as IVs. Associations with *P* < 0.05 estimated by the IVW method were considered significant.

Finally, a significant metabolite-cancer association was considered confident if it met a series of stringent criteria: (1) the significance of association reached Bonferroni-corrected *P* < 0.05 using IVW as well as *P* < 0.05 using at least one of the other three approaches, (2) the association pattern was consistent across all MR approaches, (3) all IVs had an *F*-statistics of > 10, (4) there was no significant heterogeneity among IVs, (5) there was no evidence of horizontal pleiotropy (Egger *P* for intercept > 0.05 and MR-PRESSO global test *P* > 0.05), and (6) MR estimates were not significantly affected by a single IV in LOO analyses. All statistical analyses were conducted using the R packages *TwoSampleMR* (v0.5.7) [38] and *MR-PRESSO* (v1.0) [25].

### Power calculation

To evaluate the statistical power of MR estimates, we utilized a specialized online tool (https://shiny.cnsgenomics.com/mRnd/) [39]. This tool employs asymptotic theory to estimate power values for the detection of causal effects derived from IVs. We performed power calculations at a type I error rate of 0.05, taking into account parameters such as *R*^2^ of IVs, the proportion of cases of cancer GWAS, and the odds ratio (OR) of MR analyses using the IVW method.

### Multivariable MR

To determine the direct impact of each plasma metabolite on cancer risk, while accounting for the effects of other metabolites, we performed multivariable MR (MVMR) analyses using the R package *MVMR* (v 0.4) [27]. MVMR effectively manages the complexities arising from interdependencies among genetic variations linked to different exposures by including multiple exposures that interact with one another [27]. For each cancer type, MVMR were performed employing all IVs involved in significant metabolite-cancer associations identified in univariate MR analyses.

### Genetic correlation and colocalization analysis

MR estimates can violate causal effects in the presence of a genetic correlation between the exposure and the outcome of interest. To address this, we conducted genetic correlation analyses using linkage disequilibrium score regression (LDSC, v2.0.1), which estimates coinheritance using chi-squared statistics based on the full summary statistics of two traits [23, 40]. On the other hand, studies have suggested that colocalization analysis could complement MR by addressing its limitations related to pleiotropy and linkage disequilibrium, providing a more nuanced understanding of the shared genetic underpinnings of exposures and outcomes [41]. For each significant association identified in primary analyses, we examined the colocalization between the metabolite and the risk of cancer at each genomic locus where each IV resides using the R package *coloc* (v5.2.2) [42] to investigate whether identified causal associations between metabolites and each cancer risk were driven by high LD, as reported in a previous study [43]. A posterior probability (PP4) of > 0.5 was considered as evidence for moderate colocalization.

## Results

### Genetic IVs

We obtained summary statistics for a total of 517,882 associations between 162,261 common genetic variants (MAF > 0.05) and 913 metabolites at *P* < 10^−5^ from the study by Surendran et al. [17]. At the LD clumping criteria of pairwise LD *r*^2^ < 0.1 within a 500 kb window and the significance threshold of *P* < 10^−6^, 911 of these 913 had at least 1 IV. After excluding weak IVs based on *F*-statistics < 10 and the Steiger test, and outliers (MR-PRESSO outliner test *P* < 0.05), 579 metabolites, each with at least 3 IVs (median 7; interquartile range [IQR] 4–20), were retained for MR analyses. The detailed information on the IVs selected for downstream MR analyses is shown in Additional file 1: Table S2.

### Overall MR results

**Fig. 2:**
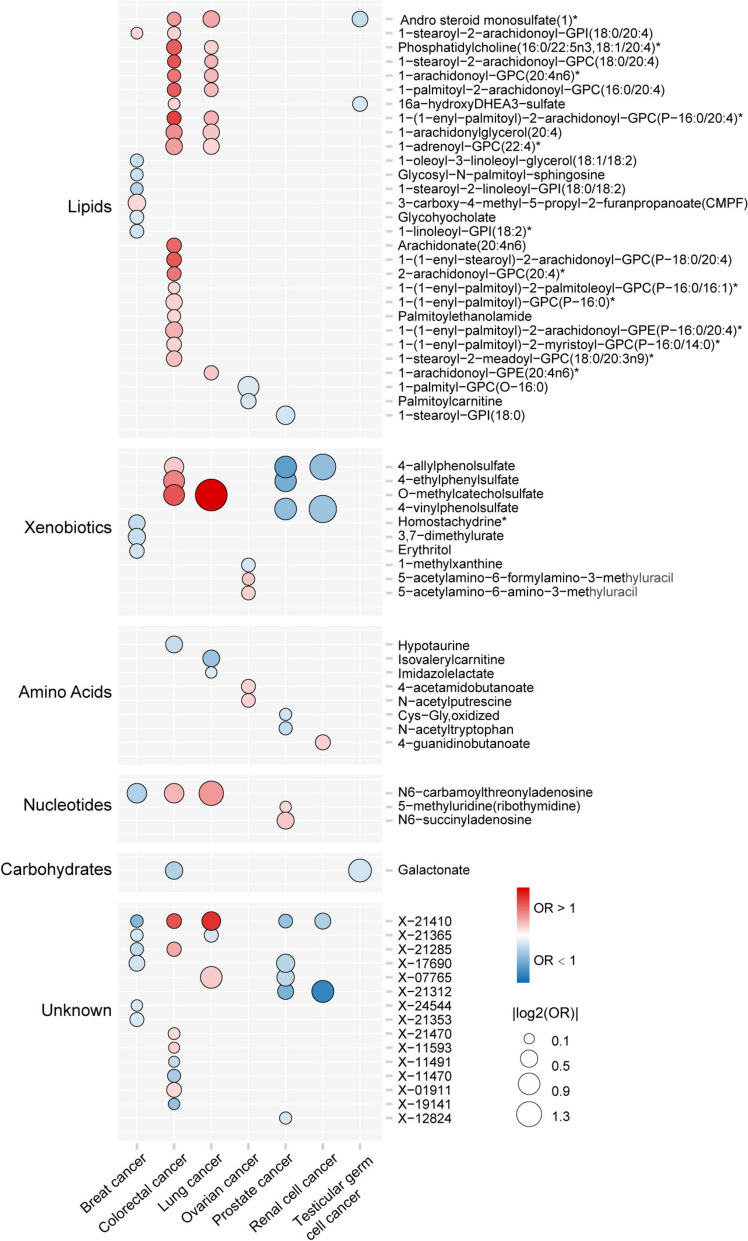
Dot plot displaying IVW-based MR estimates of significant associations with Bonferroni-corrected *P* < 0.05 within each cancer type. OR, odds ratio. The *x*-axis represents seven different cancer types, while the *y*-axis corresponds to the identified metabolites. Circle size indicates the OR, with red indicating ORs greater than 1 and blue indicating ORs smaller than 1. A more intense red or blue color signifies associations with smaller *P* values

At Bonferroni-corrected *P* < 0.05 for each cancer type, we identified a total of 94 significant associations, including 17 for breast cancer, 33 for colorectal cancer, 16 for lung cancer, 7 for ovarian cancer, 13 for prostate cancer, 5 for renal cell cancer, and 3 for testicular germ cell cancer (Fig. 2 and Additional file 1: Table S3). The median variance in plasma metabolite levels explained by the IVs for these associations was 10.20% (IQR 2.68–18.07%) (Additional file 1: Tables S3). Scatter plots illustrating these associations are presented in Additional file 2: Fig. S1. Out of the 66 distinct metabolites involved in these associations, 45 (68.20%) were associated with the risk of a specific cancer (Table 1), while the remaining 21 metabolites were each associated with risk of at least two different cancers (Table 2). These 66 metabolites comprised 29 lipids, 10 xenobiotics, 8 amino acids, 3 nucleotides, 1 carbohydrate, and 15 compounds that are not yet well annotated (Fig. 2). To evaluate the robustness of these significant associations, we performed MR analyses using IVs that were selected under a more stringent threshold, i.e., *P* < 5 × 10^−8^, LD *r*^2^ < 0.001, window size of 1000 kb. Of these 94 associations, 76 had a sufficient number of IVs (> 3), 50 of which showed *P* < 0.05 using the IVW method. Under the Bonferroni-corrected *P* < 0.05, 11 of these associations remained significant (Additional file 1: Table S4).

**Table 1:** Mendelian randomization (MR) results for metabolites that were significantly associated with the risk of a particular cancer type using the inverse-variance weighted (IVW) approach

Metabolite ID	Annotation	Number of IVs	IVW			Weighted median		Egger regression		MR-PRESSO	
			**OR (95% CI)**	***P***	***P***_**bonfer**_	**OR (95% CI)**	***P***	**OR (95% CI)**	***P***	**OR (95% CI)**	***P***
**Breast cancer**											
M52468	1-Stearoyl-2-linoleoyl-GPI (18:0/18:2)	30	0.93 (0.91–0.95)	1.67 × 10^−8^	7.96 × 10^−6^	0.94 (0.90–0.98)	7.46 × 10^−3^	0.92 (0.87–0.98)	0.01	0.93 (0.91–0.95)	1.33 × 10^−6^
M53013	Glycosyl-N-palmitoyl-sphingosine	21	0.93 (0.90–0.96)	2.61 × 10^−6^	1.24 × 10^−3^	0.96 (0.91–1.00)	0.06	0.97 (0.91–1.04)	0.45	0.93 (0.90–0.96)	1.38 × 10^−4^
M46799	1-Oleoyl-3-linoleoyl-glycerol (18:1/18:2)	20	0.92 (0.89–0.95)	1.42 × 10^−6^	6.78 × 10^−4^	0.90 (0.83–0.97)	0.02	0.94 (0.82–1.07)	0.33	0.92 (0.89–0.95)	3.26 × 10^−5^
M36594	1-Linoleoyl-GPI (18:2)*	9	0.91 (0.87–0.95)	1.63 × 10^−5^	7.75 × 10^−3^	0.89 (0.83–0.95)	0.01	0.92 (0.78–1.09)	0.38	0.91 (0.87–0.95)	2.58 × 10^−3^
M42574	Glycohyocholate	8	0.91 (0.87–0.95)	5.72 × 10^−5^	0.03	0.94 (0.87–1.02)	0.21	0.96 (0.83–1.11)	0.60	0.91 (0.88–0.94)	1.19 × 10^−3^
M20699	Erythritol	6	0.89 (0.85–0.94)	1.32 × 10^−5^	6.31 × 10^−3^	0.91 (0.84–0.97)	0.04	0.93 (0.85–1.02)	0.18	0.89 (0.86–0.94)	4.60 × 10^−3^
M31787	3-Carboxy-4-methyl-5-propyl-2-furanpropanoate (CMPF)	5	1.24 (1.12–1.39)	8.84 × 10^−5^	0.04	1.33 (1.10–1.60)	0.04	1.15 (0.69–1.94)	0.63	1.24 (1.12–1.39)	0.02
M33009	Homostachydrine*	4	0.84 (0.78–0.90)	4.37 × 10^−7^	2.09 × 10^−4^	0.85 (0.78–0.94)	0.04	0.95 (0.71–1.28)	0.79	0.84 (0.80–0.87)	3.21 × 10^−3^
M34399	3,7-Dimethylurate	4	0.82 (0.75–0.89)	7.89 × 10^−7^	3.77 × 10^−4^	0.79 (0.70–0.89)	0.03	0.99 (0.62–1.57)	0.96	0.82 (0.76–0.88)	0.01
M52865	X-24544	26	0.95 (0.93–0.97)	5.62 × 10^−5^	0.03	0.95 (0.91–1.00)	0.05	1.00 (0.94–1.06)	0.97	0.95 (0.93–0.97)	1.14 × 10^−4^
M46398	X-21353	7	0.90 (0.86–0.95)	9.53 × 10^−5^	0.04	0.91 (0.86–0.97)	0.03	0.91 (0.80–1.03)	0.20	0.90 (0.87–0.94)	2.56 × 10^−3^
**Colorectal cancer**											
M33387	2-Arachidonoyl-GPC (20:4)*	21	1.11 (1.08–1.13)	4.13 × 10^−15^	2.07 × 10^−12^	1.11 (1.08–1.14)	1.84 × 10^−6^	1.11 (1.05–1.16)	5.55 × 10^−4^	1.11 (1.08–1.13)	1.56 × 10^−7^
M52713	1-(1-Enyl-palmitoyl)-2-palmitoleoyl-GPC (P-16:0/16:1)*	21	1.06 (1.03–1.09)	7.60 × 10^−5^	0.04	1.08 (1.03–1.13)	2.99 × 10^−3^	1.05 (0.99–1.12)	0.12	1.06 (1.03–1.09)	3.47 × 10^−4^
M01110	Arachidonate (20:4n6)	20	1.13 (1.10–1.16)	5.62 × 10^−17^	2.82 × 10^−14^	1.14 (1.10–1.19)	1.18 × 10^−6^	1.15 (1.09–1.21)	4.41 × 10^−5^	1.13 (1.10–1.16)	1.03 × 10^−8^
M52613	1-(1-Enyl-stearoyl)-2-arachidonoyl-GPC (P-18:0/20:4)	20	1.13 (1.10–1.16)	1.61 × 10^−18^	8.04 × 10^−16^	1.12 (1.08–1.16)	2.98 × 10^−6^	1.13 (1.08–1.19)	1.14 × 10^−4^	1.13 (1.10–1.16)	6.35 × 10^−9^
M00590	Hypotaurine	9	0.83 (0.78–0.90)	5.84 × 10^−7^	2.93 × 10^−4^	0.86 (0.75–0.99)	0.07	0.73 (0.58–0.93)	0.03	0.83 (0.79–0.88)	8.75 × 10^−5^
M52673	1-(1-Enyl-palmitoyl)-2-arachidonoyl-GPE (P-16:0/20:4)*	9	1.21 (1.14–1.30)	3.92 × 10^−9^	1.97 × 10^−6^	1.25 (1.16–1.35)	3.87 × 10^−4^	1.32 (1.16–1.49)	3.71 × 10^−3^	1.21 (1.14–1.30)	3.67 × 10^−4^
M52715	1-(1-Enyl-palmitoyl)-2-myristoyl-GPC (P-16:0/14:0)*	9	1.13 (1.07–1.19)	2.35 × 10^−5^	0.01	1.12 (1.04–1.22)	0.02	1.04 (0.90–1.21)	0.59	1.13 (1.07–1.19)	2.88 × 10^−3^
M52738	1-Stearoyl-2-meadoyl-GPC (18:0/20:3n9)*	9	1.16 (1.10–1.23)	1.86 × 10^−7^	9.31 × 10^−5^	1.19 (1.13–1.25)	1.88 × 10^−4^	1.21 (1.10–1.34)	7.19 × 10^−3^	1.16 (1.10–1.23)	8.09 × 10^−4^
M38165	Palmitoyl ethanolamide	8	1.09 (1.05–1.14)	6.08 × 10^−5^	0.03	1.10 (1.03–1.16)	0.02	1.07 (0.95–1.22)	0.32	1.09 (1.06–1.13)	1.58 × 10^−3^
M52474	1-(1-Enyl-palmitoyl)-GPC (P-16:0)*	7	1.19 (1.10–1.29)	2.22 × 10^−5^	0.01	1.22 (1.07–1.40)	0.02	0.97 (0.71–1.32)	0.85	1.19 (1.10–1.29)	4.20 × 10^−3^
M46701	X-19141	70	0.95 (0.94–0.97)	3.08 × 10^−12^	1.54 × 10^−9^	0.95 (0.93–0.97)	1.14 × 10^−6^	0.96 (0.94–0.98)	5.96 × 10^−4^	0.95 (0.94–0.96)	5.08 × 10^−12^
M46601	X-11470	55	0.92 (0.90–0.95)	2.30 × 10^−10^	1.15 × 10^−7^	0.92 (0.86–0.98)	8.76 × 10^−3^	0.93 (0.87–1.00)	0.06	0.92 (0.90–0.95)	1.70 × 10^−8^
M47651	X-11491	45	0.96 (0.95–0.98)	3.72 × 10^−7^	1.86 × 10^−4^	0.96 (0.94–0.98)	4.13 × 10^−5^	0.95 (0.92–0.97)	5.86 × 10^−5^	0.96 (0.95–0.98)	5.19 × 10^−6^
M47653	X-11593	34	1.04 (1.02–1.06)	1.50 × 10^−5^	7.49 × 10^−3^	1.03 (1.01–1.06)	4.69 × 10^−3^	1.01 (0.98–1.04)	0.48	1.04 (1.02–1.05)	1.81 × 10^−6^
M46515	X-21470	24	1.06 (1.03–1.09)	7.74 × 10^−5^	0.04	1.04 (1.00–1.08)	0.05	1.03 (0.98–1.09)	0.26	1.06 (1.03–1.09)	6.33 × 10^−4^
M46459	X-01911	6	1.13 (1.06–1.20)	9.52 × 10^−5^	0.05	1.12 (1.03–1.22)	0.04	1.15 (0.97–1.37)	0.19	1.13 (1.07–1.20)	8.61 × 10^−3^
**Lung cancer**											
M34407	Isovalerylcarnitine	21	0.83 (0.79–0.88)	9.37 × 10^−12^	4.25 × 10^−9^	0.80 (0.73–0.88)	1.09 × 10^−4^	0.80 (0.69–0.94)	0.01	0.83 (0.80–0.87)	5.25 × 10^−8^
M35186	1-Arachidonoyl-GPE (20:4n6)*	24	1.12 (1.07–1.17)	1.32 × 10^−6^	5.97 × 10^−4^	1.10 (1.03–1.17)	8.96 × 10^−3^	1.12 (1.02–1.22)	0.03	1.12 (1.08–1.16)	3.89 × 10^−6^
M15716	Imidazole lactate	44	0.95 (0.92–0.97)	7.22 × 10^−5^	0.03	0.96 (0.92–0.99)	0.02	0.95 (0.90–1.00)	0.05	0.95 (0.93–0.97)	4.70 × 10^−5^
M46410	X-21365	25	0.90 (0.85–0.94)	3.09 × 10^−5^	0.01	0.88 (0.81–0.96)	7.63 × 10^−3^	0.90 (0.80–1.00)	0.07	0.90 (0.85–0.94)	2.45 × 10^−4^
**Ovarian cancer**											
M34401	5-Acetylamino-6-formylamino-3-methyluracil	41	1.07 (1.04–1.09)	8.96 × 10^−7^	4.36 × 10^−4^	1.06 (1.03–1.10)	1.77 × 10^−3^	1.05 (1.01–1.10)	0.02	1.07 (1.04–1.09)	6.19 × 10^−6^
M34424	5-Acetylamino-6-amino-3-methyluracil	28	1.10 (1.05–1.15)	1.71 × 10^−5^	8.33 × 10^−3^	1.11 (1.05–1.18)	9.77 × 10^−4^	1.12 (1.04–1.21)	6.68 × 10^−3^	1.10 (1.05–1.15)	1.53 × 10^−4^
M01558	4-Acetamidobutanoate	25	1.11 (1.06–1.16)	2.20 × 10^−5^	0.01	1.12 (1.06–1.19)	9.65 × 10^−4^	1.14 (1.05–1.24)	5.08 × 10^−3^	1.11 (1.06–1.16)	1.48 × 10^−4^
M37496	N-acetylputrescine	25	1.10 (1.06–1.15)	1.44 × 10^−5^	7.03 × 10^−3^	1.11 (1.05–1.18)	1.93 × 10^−3^	1.13 (1.05–1.22)	5.19 × 10^−3^	1.10 (1.06–1.15)	2.87 × 10^−5^
M34389	1-Methylxanthine	22	0.91 (0.87–0.95)	3.50 × 10^−5^	0.02	0.89 (0.83–0.95)	1.70 × 10^−3^	0.86 (0.79–0.94)	2.18 × 10^−3^	0.91 (0.87–0.95)	1.90 × 10^−4^
M44681	Palmitoylcarnitine	22	0.87 (0.82–0.93)	4.14 × 10^−5^	0.02	0.86 (0.77–0.97)	0.02	0.82 (0.69–0.97)	0.03	0.87 (0.83–0.92)	3.25 × 10^−5^
M20458	1-Palmityl-GPC (O-16:0)	5	0.70 (0.58–0.84)	9.85 × 10^−5^	0.05	0.67 (0.51–0.90)	0.05	0.86 (0.43–1.74)	0.71	0.70 (0.65–0.74)	4.01 × 10^−4^
**Prostate cancer**											
M18368	Cys-Gly, oxidized	36	0.94 (0.91–0.96)	5.51 × 10^−6^	2.71 × 10^−3^	0.92 (0.87–0.97)	3.29 × 10^−3^	0.91 (0.85–0.98)	0.01	0.94 (0.91–0.96)	3.66 × 10^−5^
M35136	5-Methyluridine (ribothymidine)	31	1.05 (1.03–1.08)	5.65 × 10^−5^	0.03	1.06 (1.02–1.10)	4.71 × 10^−3^	1.06 (1.01–1.12)	0.02	1.05 (1.03–1.07)	4.63 × 10^−6^
M33959	N-acetyltryptophan	24	0.92 (0.89–0.95)	4.15 × 10^−7^	2.04 × 10^−4^	0.92 (0.87–0.98)	0.01	0.94 (0.87–1.01)	0.11	0.92 (0.89–0.95)	2.30 × 10^−5^
M19324	1-Stearoyl-GPI (18:0)	5	0.78 (0.70–0.87)	1.49 × 10^−5^	7.33 × 10^−3^	0.83 (0.68–1.01)	0.13	0.54 (0.13–2.29)	0.47	0.78 (0.70–0.87)	0.01
M48130	N6-succinyladenosine	4	1.21 (1.12–1.30)	9.48 × 10^−7^	4.66 × 10^−4^	1.19 (1.09–1.31)	0.03	1.22 (0.97–1.54)	0.23	1.21 (1.12–1.30)	0.02
M46471	X-12824	31	0.94 (0.91–0.97)	3.60 × 10^−5^	0.02	0.94 (0.90–0.99)	0.02	0.98 (0.92–1.04)	0.43	0.94 (0.91–0.97)	2.66 × 10^−4^
**Renal cell cancer**											
M15681	4-Guanidinobutanoate	22	1.14 (1.08–1.21)	8.67 × 10^−6^	4.12 × 10^−3^	1.12 (1.04–1.22)	8.39 × 10^−3^	1.14 (1.02–1.27)	0.03	1.14 (1.08–1.20)	8.59 × 10^−5^

*IVs* instrumental variables, *OR* odds ratio per standard deviation (SD) in genetically predicted plasma metabolite level, *CI* confidence interval, *P*_*bonfer*_ Bonferroni-corrected *P* value within each cancer type, *MR-PRESSO* Mendelian Randomization Pleiotropy RESidual Sum and Outlier

**Table 2:** MR results for metabolites that were significantly associated with the risk of more than one cancer type using the IVW approach

Metabolite ID	Annotation	Cancer	Number of IVs	IVW			Weighted median		Egger regression		MR-PRESSO	
				**OR (95% CI)**	***P***	***P***_**bonfer**_	**OR (95% CI)**	***P***	**OR (95% CI)**	***P***	**OR (95% CI)**	***P***
M32827	Andro steroid monosulfate (1)*	Colorectal	38	1.10 (1.07–1.13)	2.18 × 10^−11^	1.09 × 10^−8^	1.05 (1.01–1.10)	0.03	1.06 (1.00–1.13)	0.07	1.10 (1.07–1.13)	7.28 × 10^−8^
		Lung	22	1.20 (1.13–1.27)	3.26 × 10^−10^	1.48 × 10^−7^	1.25 (1.09–1.43)	4.22 × 10^−3^	1.22 (1.02–1.45)	0.04	1.20 (1.13–1.27)	3.11 × 10^−6^
		Testicular germ cell	53	0.86 (0.80–0.91)	1.04 × 10^−6^	5.00 × 10^−4^	0.86 (0.78–0.96)	9.26 × 10^−3^	0.88 (0.77–1.00)	0.05	0.86 (0.80–0.91)	1.03 × 10^−5^
M52689	1-(1-Enyl-palmitoyl)-2-arachidonoyl-GPC (P-16:0/20:4)*	Colorectal	32	1.11 (1.09–1.14)	2.92 × 10^−21^	1.46 × 10^−18^	1.12 (1.08–1.15)	1.21 × 10^−7^	1.12 (1.07–1.16)	1.14 × 10^−5^	1.11 (1.09–1.14)	9.51 × 10^−11^
		Lung	29	1.12 (1.08–1.16)	7.29 × 10^−9^	3.31 × 10^−6^	1.10 (1.03–1.16)	4.10 × 10^−3^	1.10 (1.03–1.17)	9.51 × 10^−3^	1.12 (1.08–1.15)	1.29 × 10^−7^
M33228	1-Arachidonoyl-GPC (20:4n6)*	Colorectal	26	1.09 (1.07–1.11)	1.95 × 10^−15^	9.78 × 10^−13^	1.10 (1.07–1.13)	7.19 × 10^−7^	1.09 (1.05–1.13)	4.32 × 10^−5^	1.09 (1.07–1.11)	4.17 × 10^−9^
		Lung	27	1.10 (1.06–1.13)	3.49 × 10^−8^	1.58 × 10^−5^	1.08 (1.04–1.14)	1.86 × 10^−3^	1.09 (1.04–1.15)	3.11 × 10^−3^	1.10 (1.06–1.13)	7.38 × 10^−6^
M52462	1-Palmitoyl-2-arachidonoyl-GPC (16:0/20:4)	Colorectal	27	1.10 (1.08–1.13)	3.27 × 10^−18^	1.64 × 10^−15^	1.10 (1.07–1.13)	3.26 × 10^−7^	1.10 (1.06–1.14)	5.62 × 10^−5^	1.10 (1.08–1.13)	3.53 × 10^−9^
		Lung	27	1.10 (1.07–1.14)	5.56 × 10^−8^	2.52 × 10^−5^	1.09 (1.05–1.14)	2.76 × 10^−4^	1.10 (1.03–1.17)	6.29 × 10^−3^	1.10 (1.07–1.14)	1.08 × 10^−5^
M42450	1-Stearoyl-2-arachidonoyl-GPC (18:0/20:4)	Colorectal	25	1.09 (1.07–1.11)	6.62 × 10^−19^	3.32 × 10^−16^	1.09 (1.06–1.12)	1.05 × 10^−6^	1.08 (1.05–1.12)	8.22 × 10^−5^	1.09 (1.07–1.11)	4.29 × 10^−9^
		Lung	25	1.09 (1.06–1.13)	1.52 × 10^−8^	6.91 × 10^−6^	1.08 (1.04–1.13)	8.84 × 10^−4^	1.07 (1.02–1.13)	0.01	1.09 (1.06–1.13)	6.71 × 10^−6^
M52719	Phosphatidylcholine (16:0/22:5n3, 18:1/20:4)*	Colorectal	21	1.14 (1.10–1.17)	1.03 × 10^−17^	5.16 × 10^−15^	1.15 (1.10–1.19)	1.24 × 10^−6^	1.16 (1.10–1.22)	2.40 × 10^−5^	1.14 (1.10–1.17)	3.96 × 10^−8^
		Lung	23	1.10 (1.06–1.16)	1.25 × 10^−5^	5.66 × 10^−3^	1.11 (1.04–1.18)	3.61 × 10^−3^	1.13 (1.05–1.23)	6.44 × 10^−3^	1.10 (1.06–1.15)	2.11 × 10^−4^
M52698	1-Adrenoyl-GPC (22:4)*	Colorectal	9	1.20 (1.14–1.27)	6.02 × 10^−11^	3.01 × 10^−8^	1.25 (1.17–1.34)	2.01 × 10^−4^	1.30 (1.16–1.44)	2.14 × 10^−3^	1.20 (1.14–1.27)	1.80 × 10^−4^
		Lung	9	1.17 (1.08–1.27)	6.76 × 10^−5^	0.03	1.17 (1.05–1.29)	0.02	1.21 (0.99–1.48)	0.10	1.17 (1.09–1.27)	3.99 × 10^−3^
M46111	O-methylcatechol sulfate	Colorectal	9	1.41 (1.31–1.52)	6.25 × 10^−19^	3.13 × 10^−16^	1.49 (1.25–1.78)	2.35 × 10^−3^	1.87 (1.34–2.62)	8.21 × 10^−3^	1.41 (1.31–1.51)	1.18 × 10^−5^
		Lung	4	2.81 (2.33–3.37)	4.32 × 10^−28^	1.96 × 10^−25^	2.85 (2.04–4.00)	8.86 × 10^−3^	4.03 (1.35–12.06)	0.13	2.81 (2.48–3.17)	4.82 × 10^−4^
M34397	1-Arachidonylglycerol (20:4)	Colorectal	6	1.18 (1.13–1.24)	9.69 × 10^−13^	4.86 × 10^−10^	1.20 (1.14–1.27)	1.50 × 10^−3^	1.28 (1.13–1.45)	0.02	1.18 (1.13–1.23)	4.19 × 10^−4^
		Lung	8	1.19 (1.11–1.27)	7.87 × 10^−7^	3.57 × 10^−4^	1.14 (1.05–1.24)	0.02	1.07 (0.93–1.23)	0.40	1.19 (1.13–1.25)	4.07 × 10^−4^
M35157	N6-carbamoylthreonyladenosine	Colorectal	6	1.34 (1.21–1.49)	1.83 × 10^−8^	9.19 × 10^−6^	1.24 (1.00–1.54)	0.11	1.97 (1.19–3.27)	0.06	1.34 (1.22–1.48)	1.65 × 10^−3^
		Lung	5	1.71 (1.47–2.00)	1.38 × 10^−11^	6.28 × 10^−9^	1.61 (1.23–2.12)	0.03	2.77 (1.36–5.66)	0.07	1.71 (1.48–1.99)	2.11 × 10^−3^
		Breast	7	0.75 (0.68–0.82)	3.81 × 10^−9^	1.82 × 10^−6^	0.84 (0.73–0.97)	0.05	0.65 (0.41–1.03)	0.13	0.75 (0.68–0.82)	1.06 × 10^−3^
M46455	X-21410	Colorectal	45	1.13 (1.10–1.16)	3.61 × 10^−19^	1.81 × 10^−16^	1.15 (1.08–1.24)	1.97 × 10^−4^	1.19 (1.12–1.26)	3.15 × 10^−6^	1.13 (1.10–1.16)	1.83 × 10^−11^
		Lung	25	1.29 (1.22–1.35)	7.06 × 10^−23^	3.21 × 10^−20^	1.24 (1.14–1.35)	3.79 × 10^−5^	1.17 (1.05–1.30)	0.01	1.29 (1.22–1.35)	6.64 × 10^−10^
		Breast	45	0.93 (0.91–0.94)	5.24 × 10^−14^	2.50 × 10^−11^	0.93 (0.90–0.97)	2.44 × 10^−3^	0.94 (0.90–0.99)	0.01	0.93 (0.91–0.94)	1.95 × 10^−9^
		Prostate	46	0.91 (0.89–0.93)	5.48 × 10^−12^	2.70 × 10^−9^	0.89 (0.84–0.95)	6.35 × 10^−4^	0.88 (0.83–0.94)	3.37 × 10^−4^	0.91 (0.89–0.93)	1.47 × 10^−8^
		Renal cell	46	0.85 (0.81–0.90)	1.19 × 10^−9^	5.66 × 10^−7^	0.94 (0.85–1.04)	0.24	0.89 (0.80–0.99)	0.04	0.85 (0.81–0.90)	1.62 × 10^−7^
M36098	4-Vinylphenol sulfate	Prostate	8	0.68 (0.61–0.76)	9.77 × 10^−13^	4.81 × 10^−10^	0.74 (0.62–0.90)	0.02	0.52 (0.31–0.88)	0.05	0.68 (0.61–0.76)	1.88 × 10^−4^
		Renal cell	8	0.49 (0.40–0.61)	1.77 × 10^−11^	8.43 × 10^−9^	0.41 (0.24–0.71)	0.01	0.38 (0.04–3.57)	0.43	0.49 (0.41–0.59)	1.28 × 10^−4^
M37181	4-Allylphenol sulfate	Prostate	8	0.68 (0.63–0.74)	9.30 × 10^−19^	4.58 × 10^−16^	0.64 (0.52–0.79)	4.39 × 10^−3^	0.88 (0.55–1.39)	0.60	0.68 (0.63–0.74)	4.78 × 10^−5^
		Renal cell	9	0.54 (0.45–0.64)	8.33 × 10^−13^	3.96 × 10^−10^	0.68 (0.49–0.95)	0.05	1.35 (0.45–4.09)	0.61	0.54 (0.45–0.64)	9.66 × 10^−5^
		Colorectal	5	1.32 (1.18–1.48)	2.11 × 10^−6^	1.06 × 10^−3^	1.41 (1.15–1.73)	0.03	1.13 (0.49–2.61)	0.80	1.32 (1.21–1.44)	3.56 × 10^−3^
M46356	X-21312	Prostate	25	0.85 (0.82–0.89)	3.84 × 10^−15^	1.89 × 10^−12^	0.91 (0.85–0.97)	0.01	0.79 (0.71–0.88)	3.23 × 10^−4^	0.85 (0.82–0.89)	4.31 × 10^−8^
		Renal cell	27	0.67 (0.62–0.72)	4.00 × 10^−23^	1.90 × 10^−20^	0.63 (0.52–0.77)	1.33 × 10^−4^	0.57 (0.42–0.75)	6.79 × 10^−4^	0.67 (0.62–0.72)	1.18 × 10^−11^
		Breast	24	0.86 (0.83–0.88)	5.71 × 10^−25^	2.72 × 10^−22^	0.90 (0.83–0.98)	0.03	0.82 (0.73–0.92)	2.17 × 10^−3^	0.86 (0.83–0.88)	4.20 × 10^−10^
M38168	16a-Hydroxy DHEA 3-sulfate	Colorectal	30	1.06 (1.03–1.08)	6.26 × 10^−5^	0.03	1.05 (1.01–1.09)	0.03	1.04 (0.98–1.10)	0.19	1.06 (1.03–1.08)	1.01 × 10^−4^
		Testicular germ cell	42	0.88 (0.82–0.94)	7.00 × 10^−5^	0.03	0.89 (0.82–0.97)	0.01	0.88 (0.79–0.98)	0.02	0.88 (0.82–0.94)	2.78 × 10^−4^
M27719	Galactonate	Colorectal	5	0.81 (0.76–0.87)	3.67 × 10^−9^	1.84 × 10^−6^	0.81 (0.73–0.90)	0.02	0.82 (0.64–1.04)	0.19	0.81 (0.78–0.85)	4.64 × 10^−4^
		Testicular germ cell	5	0.64 (0.52–0.78)	9.60 × 10^−6^	4.62 × 10^−3^	0.66 (0.49–0.88)	0.05	0.93 (0.46–1.86)	0.84	0.64 (0.57–0.71)	1.52 × 10^−3^
M52449	1-Stearoyl-2-arachidonoyl-GPI (18:0/20:4)	Breast	22	1.06 (1.03–1.08)	8.31 × 10^−5^	0.04	1.09 (1.03–1.15)	4.44 × 10^−3^	1.05 (0.99–1.12)	0.13	1.06 (1.03–1.08)	5.25 × 10^−4^
		Colorectal	22	1.09 (1.05–1.13)	2.27 × 10^−5^	0.01	1.06 (1.00–1.12)	0.06	1.07 (0.97–1.18)	0.20	1.09 (1.05–1.13)	3.69 × 10^−4^
M36099	4-Ethylphenylsulfate	Colorectal	8	1.42 (1.30–1.55)	5.07 × 10^−14^	2.54 × 10^−11^	1.50 (1.26–1.79)	2.66 × 10^−3^	1.52 (1.00–2.31)	0.10	1.42 (1.30–1.55)	1.34 × 10^−4^
		Prostate	8	0.69 (0.63–0.75)	9.05 × 10^−16^	4.45 × 10^−13^	0.65 (0.54–0.77)	1.91 × 10^−3^	0.76 (0.45–1.27)	0.34	0.69 (0.63–0.75)	8.83 × 10^−5^
M46294	X-21285	Breast	24	0.92 (0.89–0.95)	1.95 × 10^−7^	9.30 × 10^−5^	0.86 (0.77–0.96)	0.01	0.95 (0.82–1.11)	0.55	0.92 (0.89–0.95)	2.82 × 10^−5^
		Colorectal	25	1.12 (1.08–1.16)	8.72 × 10^−10^	4.37 × 10^−7^	1.16 (1.06–1.26)	2.40 × 10^−3^	1.20 (1.07–1.34)	4.08 × 10^−3^	1.12 (1.08–1.16)	2.47 × 10^−6^
M46590	X-07765	Lung	4	1.47 (1.25–1.73)	3.41 × 10^−6^	1.55 × 10^−3^	1.36 (1.15–1.61)	0.04	1.55 (0.84–2.86)	0.30	1.47 (1.25–1.73)	0.02
		Prostate	8	0.81 (0.75–0.87)	6.92 × 10^−8^	3.41 × 10^−5^	0.85 (0.78–0.93)	0.01	0.73 (0.56–0.95)	0.06	0.81 (0.75–0.87)	1.02 × 10^−3^
M46710	X-17690	Breast	6	0.86 (0.80–0.92)	1.06 × 10^−5^	5.06 × 10^−3^	0.89 (0.79–0.99)	0.08	0.88 (0.58–1.31)	0.56	0.86 (0.80–0.92)	6.99 × 10^−3^
		Prostate	9	0.79 (0.73–0.86)	1.69 × 10^−8^	8.33 × 10^−6^	0.85 (0.78–0.93)	6.11 × 10^−3^	0.91 (0.64–1.27)	0.59	0.79 (0.73–0.86)	4.87 × 10^−4^

*IVs* instrumental variables, *OR* odds ratio per SD in genetically predicted plasma metabolite level, *CI* confidence interval, *P*_*bonfer*_ Bonferroni-corrected *P* value within each cancer type, *MR-PRESSO* Mendelian Randomization Pleiotropy RESidual Sum and Outlier

#### Metabolites exclusively associated with the risk of a specific cancer

Of the 66 metabolites, 45 (68.2%) were each causally associated with specific cancer types, including 11 for breast cancer, 16 for colorectal cancer, 4 for lung cancer, 7 for ovarian cancer, 6 for prostate cancer, and 1 for renal cell cancer (Table 1). Intriguingly, none of the 7 metabolites associated with ovarian cancer exhibited any significant associations with the other 6 cancer types. In contrast, all of the three metabolites associated with testicular germ cell cancer were spontaneously associated with other cancers. Among these 45 metabolites, the strongest contributory effects on cancer risk were observed for 3-carboxy-4-methyl-5-propyl-2-furanpropanoate (CMPF) on breast cancer risk (OR 1.24; 95% confidence interval [CI] 1.12–1.39), 1-(1-enyl-palmitoyl)-2-arachidonoyl-GPE (P-16:0/20:4)* on colorectal cancer risk (OR 1.21; 95% CI 1.14–1.30), 1-arachidonoyl-GPE (20:4n6)* on lung cancer risk (OR 1.12; 95% CI 1.07–1.17), 4-acetamidobutanoate on ovarian cancer risk (OR 1.11; 95% CI 1.06–1.16), N6-succinyladenosine on prostate cancer risk (OR 1.21; 95% CI 1.12–1.30), and 4-guanidinobutanoate on testicular germ cell cancer risk (OR 1.14; 95% CI 1.08–1.21) (Table 1, Fig. 2). In contrast, 3,7-dimethylurate, hypotaurine, isovalerylcarnitine, 1-palmityl-GPC (O-16:0), and 1-stearoyl-GPI (18:0) showed the strongest protective effect on the risk of breast (OR 0.82; 95% CI 0.75–0.89), colorectal (OR 0.83; 95% CI 0.78–0.90), lung (OR 0.83; 95% CI 0.79–0.88), ovarian (OR 0.70; 95% CI 0.58–0.84), and prostate cancer (OR 0.78; 95% CI 0.70–0.87), respectively (Table 1, Fig. 2).

#### Metabolites associated with the risk of multiple cancers

Of the remaining 21 metabolites, 1 was associated with the risk of 5 cancers, 4 were each associated with the risk of 3 cancers, and 16 were each associated with the risk of 2 cancers (Table 2, Fig. 2). An unannotated metabolite X-21,410 showed significant association with the risk of most cancer types, including the increased risk of lung (OR 1.13; 95% CI 1.10–1.16) and colorectal (OR 1.29; 95% CI 1.22–1.35) cancers and decreased risk of breast (OR 0.93; 95% CI 0.91–0.94), prostate (OR 0.91; 95% CI 0.89–0.93), and renal cell (OR 0.85; 95% CI 0.81–0.90) cancers. Of the 16 metabolites each showing associations with 2 cancer types, 11 were each associated with colorectal and lung cancer risk, and notably, all of them exhibited contributory effects on both cancer types. Among them, O-methylcatechol sulfate showed the strongest association, the genetically predicted plasma levels of which were associated with a 1.41-fold (95% CI 1.31–1.52) and 2.81-fold (95% CI 2.33–3.37) increased risk of colorectal cancer and lung cancer, respectively (Table 2, Fig. 2). Conversely, 4 metabolites were each associated with both prostate and renal cell cancer, and all of them were protective against the risk of both cancers. Among them, 4-vinylphenol sulfate displayed the most pronounced protective effects, with ORs of 0.68 (95% CI 0.61–0.76) for prostate cancer and 0.49 (95% CI 0.40–0.61) for renal cell cancer (Table 2, Fig. 2).

### Complementary, sensitivity, and reverse MR analyses

As shown in Additional file 1: Table S3, all the 94 significant associations identified using the IVW method consistently demonstrated the same association patterns in the results from all 3 additional MR approaches. Notably, more than 90% (85) of these associations showed *P* < 0.05 in at least 2 of the 3 additional MR analyses. Notably, none of these 94 associations was influenced by horizontal pleiotropy, as evidenced by Egger regression (all *P*_intercept_ > 0.05) and MR-PRESSO global test (all *P* > 0.05) (Additional file 1: Table S3). In addition, no significant heterogeneity was detected among IVs for any of these 94 associations (*P* for heterogeneity > 0.05). Further, LOO analyses confirmed that none of these 94 associations was dominated by a single IV. The statistical power of all MR estimates based on the IVW methods ranged from 0.98 to 1.00 (Additional file 1: Table S3). In reverse MR analyses, 195, 133, 15, 12, 158, 12, and 48 SNPs were selected as IVs for breast, colorectal, lung, ovarian, prostate, renal cell, and testicular germ cell cancers, respectively (Additional file 1: Table S5). Utilizing these IVs and the IVW method, none of the 94 associations showed the possibility of reverse causation (all *P* > 0.05) (Additional file 1: Table S3).

### Replication analysis using an independent plasma metabolite GWAS dataset

Of the 64 unique metabolites included in the 94 significant associations, 44 metabolites in 64 associations had available data in the CLSA study. After selecting IVs using the same criteria, MR analyses were performed using the same cancer GWAS data with IVW as the primary method, supplemented by weighted median, Egger regression, and MR-PRESSO. Remarkably, all but 2 of these 64 associations showed an association direction that is consistent with those observed analyses using EPIC-Norfold and INTERVAL data, and 54 of them (84.4%) even reached the nominal significance of *P* < 0.05 (Additional file 1: Table S6).

### MVMR analyses

To uncover metabolites that might be directly associated with cancer risk independent of other metabolites, we conducted MVMR analyses. For each cancer type, IVs for all significant associations identified in univariate MR analyses were included in MVMR analyses using the IVW model. A total of 21 metabolites were found to be independently associated with cancer risk at MVMR *P* < 0.05, including 7 associated with breast cancer risk, 5 associated colorectal cancer risk, 2 associated with lung cancer risk, 3 associated with ovarian cancer risk, 3 associated with prostate cancer risk, and 1 associated with testicular germ cell cancer risk (Additional file 1: Table S7).

### Genetic correlation and colocalization analyses

Among the 94 identified significant associations, LDSC analyses detected nominally significant (*P* < 0.05) genetic correlations between seven metabolite-cancer pairs (Additional file 1: Table S8). This result suggests that for most of our identified significant associations, the causal effects were unlikely to be confused by the coheritability between metabolites and cancer risk. On the other hand, in colocalization analyses, 70 (74.5%) of these 94 metabolite-cancer pairs showed a moderate colocalization with (PP4 > 0.5) in at least 1 locus where their IVs reside (Additional file 1: Table S9), indicating the existence of shared causal variants between metabolites and cancer risk in these genomic regions. Among these 70 metabolite-cancer pairs, the median percentage of IVs whose loci exhibited colocalization signals was 50.0% (IQR 20.0–68.1%).

## Discussion

In this comprehensive MR study empowered by the unprecedented resources of large-scale GWAS data, we discovered 94 significant associations indicating the potential causal influences of 66 unique plasma metabolites on the risk of 7 cancers. Over two-thirds of these metabolites were exclusively identified for specific cancer types. Of the 64 associations eligible for external validation analyses, nearly 85% (54) were successfully replicated. Further, MVMR analyses revealed that 21 of these 66 metabolites likely have direct effects on cancer risk. These findings provide additional insights into the complex interplay between genetics and metabolites in cancer development, fostering the development of innovative strategies for cancer prevention and treatment.

Developing effective strategies for cancer risk assessment and prevention is critically important. The emergence of metabolomics technologies has fueled interest in exploring the clinical utility of circulating metabolites as a non-invasive biomarker, given their ability to reflect both endogenous and exogenous physiological processes [18, 44]. Metabolic molecules, such as those involved in nucleotide metabolism, have shown potential as therapeutic targets in impeding tumor progression in preclinical studies [45]. Although previous studies have identified metabolites involved in cancer mechanisms, their role in risk assessment and prevention was constrained by unclear causal links [46]. Leveraging recent large metabolite GWAS data, well-powered cancer GWAS data, and the MR framework, we systematically explored the potential causal relationship between plasma metabolites and the risk of common cancers. Our findings, if future validated by future case-control studies nested in large population-based cohorts, hold the potential to significantly contribute to the development of metabolites-based panels for cancer risk stratification and the identification of new therapeutic targets, thereby substantially improving cancer management and treatment strategies.

Although the majority of the 94 significant metabolite-cancer associations were first reported by our study, several of them are in line with the findings from previous studies. The negative association between isovalerylcarnitine, a specific activator of high calcium [47], and lung cancer risk is consistent with a recent report based on both MR and nested case-control investigations. The 4-guanidinobutanoate, an intermediate product in the polyamine synthesis pathway, was found to be associated with increased renal cell cancer risk in our study. This metabolite was previously reported to be correlated with an increased estimated glomerular filtration rate (eGFR), indicating its possible role in kidney dysfunction [48]. Oxidized Cys-Gly, which showed protective effects on prostate cancer risk in the current study, was previously found to be associated with a decreased risk of gastric cardia adenocarcinoma [49]. All of these showcase the validity of our findings.

We found 22 metabolites each showing associations with more than one cancer type. Interestingly, 11 of them were spontaneously associated with the increased risk of both lung cancer and colorectal cancer. These results might be partially explained by their shared metabolic-related risk factors, such as physical inactivity [50, 51] and a diet low in fiber [52, 53]. Future studies are needed to appraise the putative shared genetic and metabolic architecture of these two cancers. On the other hand, some metabolites showed contradictory effects on different cancers, such as N6-carbamoylthreonyladenosine, which was linked to an elevated risk of lung cancer and colorectal cancer but a reduced risk of breast cancer. This metabolite was previously correlated with elevated blood interleukin-6 in older adults, which was associated with an increased risk of cancer and mortality [54]. The possible protective effects of this metabolite on breast cancer need further investigation.

The human metabolome is profoundly influenced by a wide range of endogenous and exogenous factors, including genetic as well as dietary-, drug-, and disease-related influences, making etiologic studies interrogating its impacts on various cancers extremely difficult. MR largely overcomes those by relying upon the random assignment of alleles at conception, yet it can yield unbiased causal estimates when its assumptions are strictly followed [19]. In addition, we utilized data from the largest GWAS of untargeted plasma metabolome and cancers to date, which ensured unparalleled statistical power for selecting robust IVs with high-accuracy association estimates for MR analyses. Furthermore, a series of complementary analyses were performed to strengthen the reliability and robustness of the findings, including different MR approaches to account for the potential violation of different MR assumptions, LOO analyses to detect associations driven by a single IV, and reverse MR to assess the possibility of reverse causation. Finally, nearly 85% of significant associations that were eligible for external validation were successfully replicated, highlighting the robustness of our findings.

Our study has limitations. First, both metabolites and cancer GWASs focused on individuals of European ancestry due to the small sample sizes of such datasets in understudied populations. This hampered the evaluation of racial/ethnic disparities in metabolite-cancer associations. Second, using sex-specific GWAS for metabolites is ideal for sex-specific cancers; however, such data were not released by either the EPIC-Norfolk and INTERVAL study or the CLSA study [14, 17]. Then, investigating metabolites in cancer-relevant normal tissues would provide more etiological insights. However, metabolomic profiling of solid tissues remains a challenging task. It is well-recognized that for many metabolites, plasma levels represent the aggregation of tissue levels [18]. Therefore, the associations observed in plasma-based analyses should at least partially reflect the carcinogenic roles of these metabolites in tissues. Further, population-based cohort studies of measured plasma metabolite levels and cancer risk, as well as in vitro investigations of the functions of metabolites in cell lines or animal models, are ideal to validate our findings. However, we were unable to carry out such studies due to the unavailability of related resources.

## Conclusions

In this systemic MR study, we unveiled compelling evidence supporting putative causal links between 66 plasma metabolites and the risk of seven cancers, a large proportion of which were successfully replicated. Our results contribute to an advanced understanding of the crucial role of circulating metabolites in cancer genetics and biology. The utility of these metabolites in cancer risk assessment and prevention merits further investigation.

### Supplementary Information

**Additional file 1: Table S1.** Information on genome-wide association studies (GWAS) of cancers among individuals of European ancestry. **Table S2.** Summary statistics of instrumental variables (IVs) for the 94 significant associations identified in primary Mendelian randomization (MR) analyses. **Table S3.** Significant associations identified in primary MR analyses using the inverse-variance weighted (IVW) method. **Table S4.** MR results based on instrumental variables (IVs) selected under a more stringent threshold (*P* < 5 × 10^–8^ & LD r^2^ < 0.001) for the 94 significant associations identified in primary analyses. **Table S5.** Summary statistics of instrumental variables (IVs) used for reverse Mendelian randomization (MR) analyses for the 94 significant associations identified in primary analyses. **Table S6.** Replication analyses results using an independent metabolite GWAS dataset for the 94 significant associations identified in primary analyses. **Table S7.** Twenty-one associations that remained significant in Multivariable MR (MVMR) analyses. **Table S8.** Seven metabolite-cancer pairs showing a nominally significant genetic correlation. **Table S9.** Colocalization analyses for the 94 significant associations identified in primary analyses.

**Additional file 2: Fig. S1.** Scatter plots of the identified 94 significant metabolite-cancer associations.

## Data Availability

All used in the present study are publicly available. The original repositories of these data are described in the “Methods” section and Additional file 1: Table S1. Data that could be used to replication our findings is available in Additional file 1: Table S2 and S5.

## Abbreviations

BCAC: Breast Cancer Association Consortium
CCFR: Colon Cancer Family Registry
CLSA: Canadian Longitudinal Study on Aging
CORECT: Colorectal Cancer Transdisciplinary Study
GECCO: Genetics and Epidemiology of Colorectal Cancer Consortium
GWAS: Genome-wide association study
IV: Instrumental variable
IVW: Inverse variance-weighted
LC3: Lung Cancer Cohort Consortium
LD: Linkage disequilibrium
LDSC: Linkage disequilibrium score regression
LOO: Leave-one-out
MAF: Minor allele frequency
MR: Mendelian randomization
MR-PRESSO: Mendelian Randomization Pleiotropy RESidual Sum and Outlier
MVMR: Multivariable Mendelian randomization
OCAC: Ovarian Cancer Association Consortium
PP: Posterior probability
PRACTICAL: Prostate Cancer Association Group to Investigate Cancer Associated Alterations in the Genome
TCC: Testicular Cancer Consortium
TRICL-ILCCO: Transdisciplinary Research of Cancer in Lung of the International Lung Cancer Consortium
