# Supplementary figures and images for: Plasma metabolites and risk of seven cancers: a two-sample Mendelian randomization study among European descendants
Source: BMC Med. 2024 Mar 4;22:90. doi: 10.1186/s12916-024-03272-8 (PMC10910673; doi:10.1186/s12916-024-03272-8)

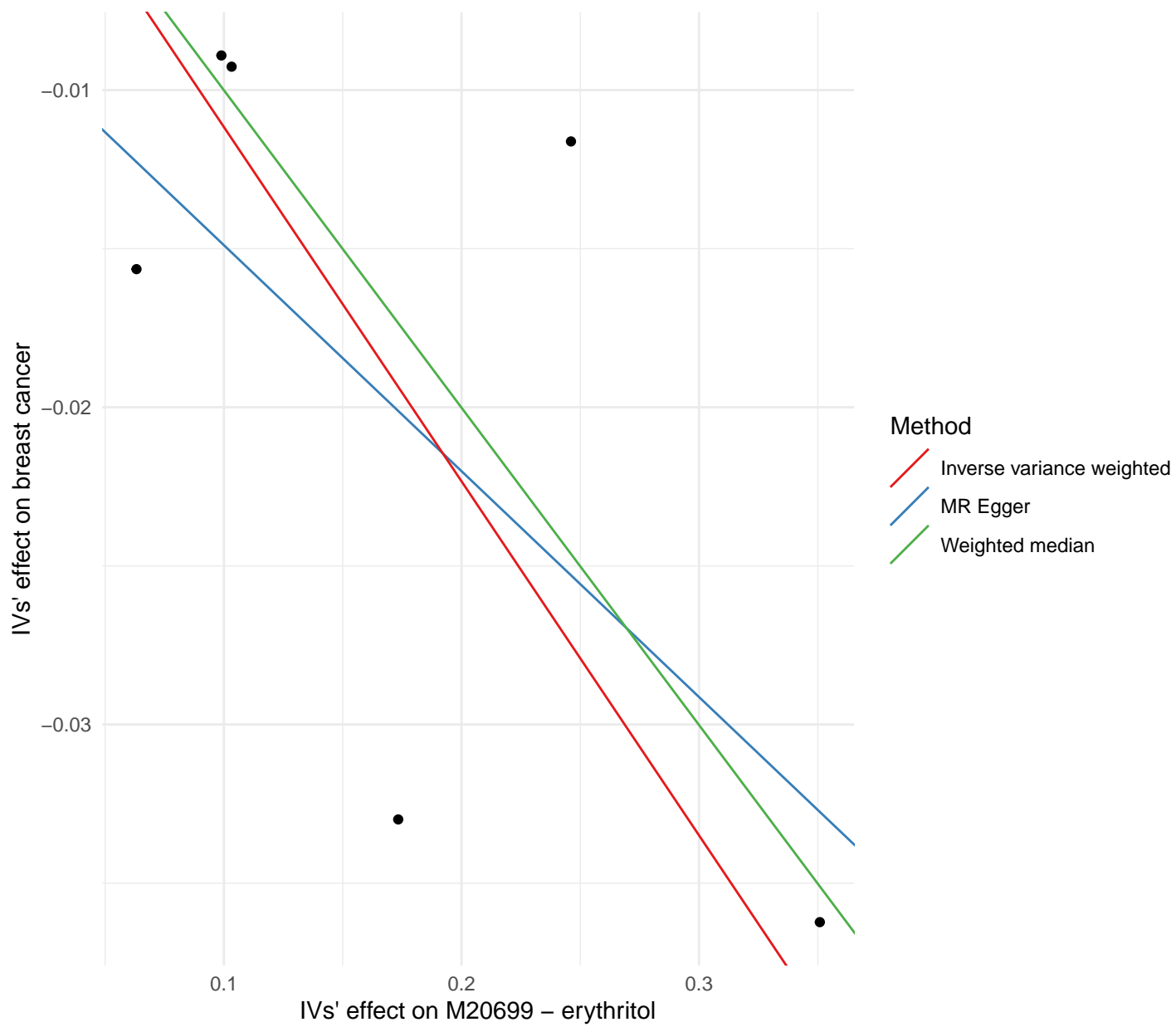

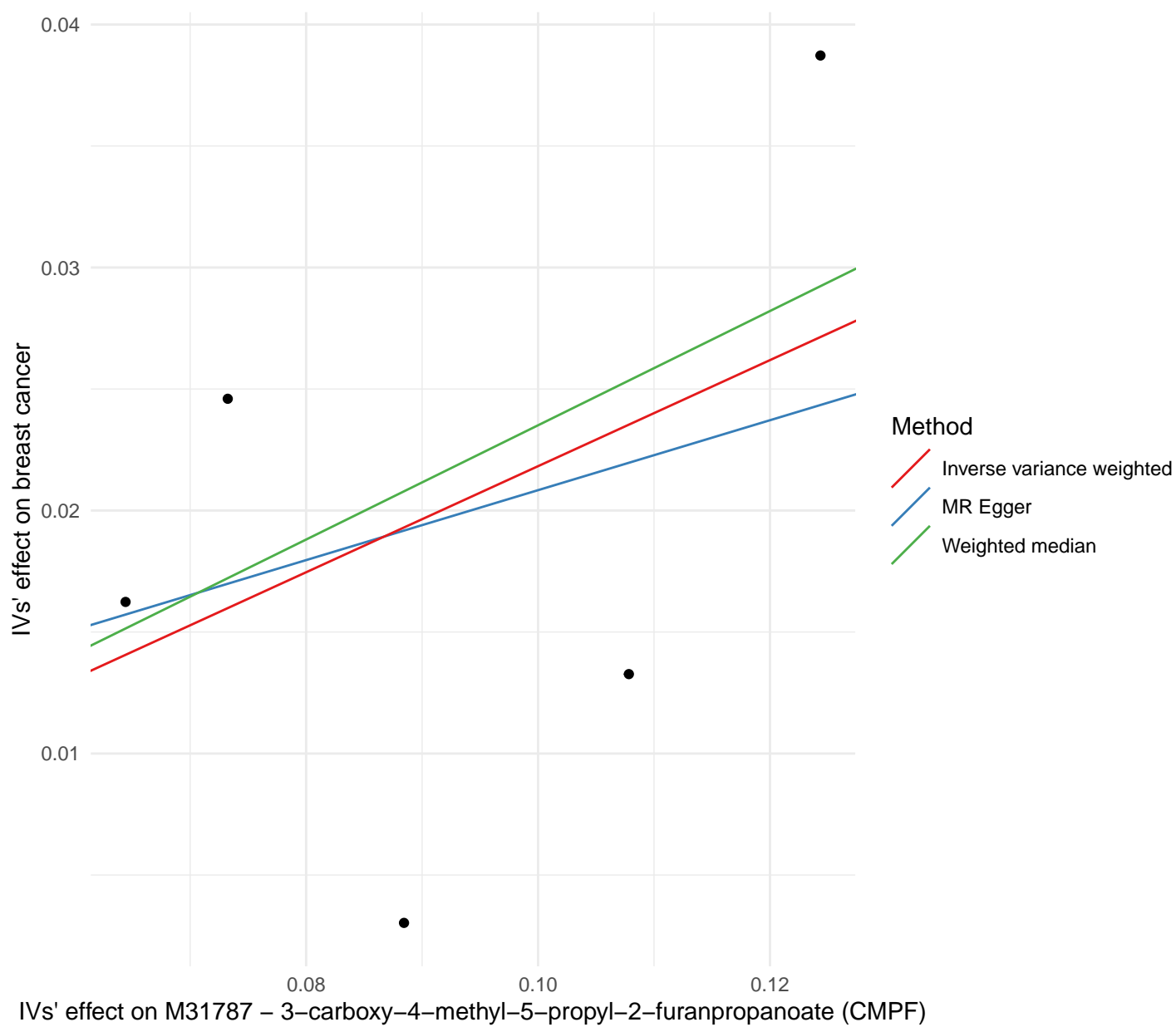

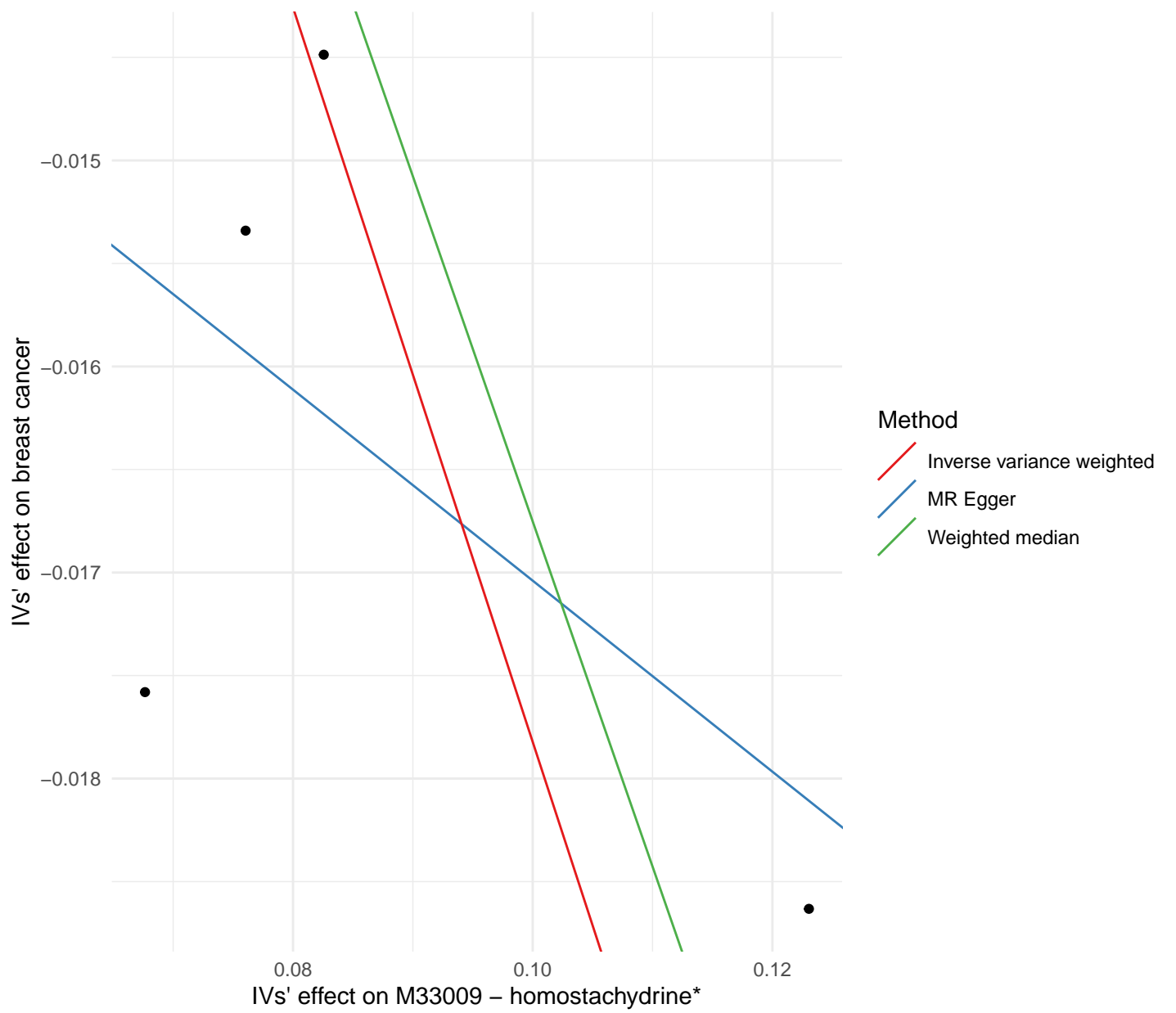

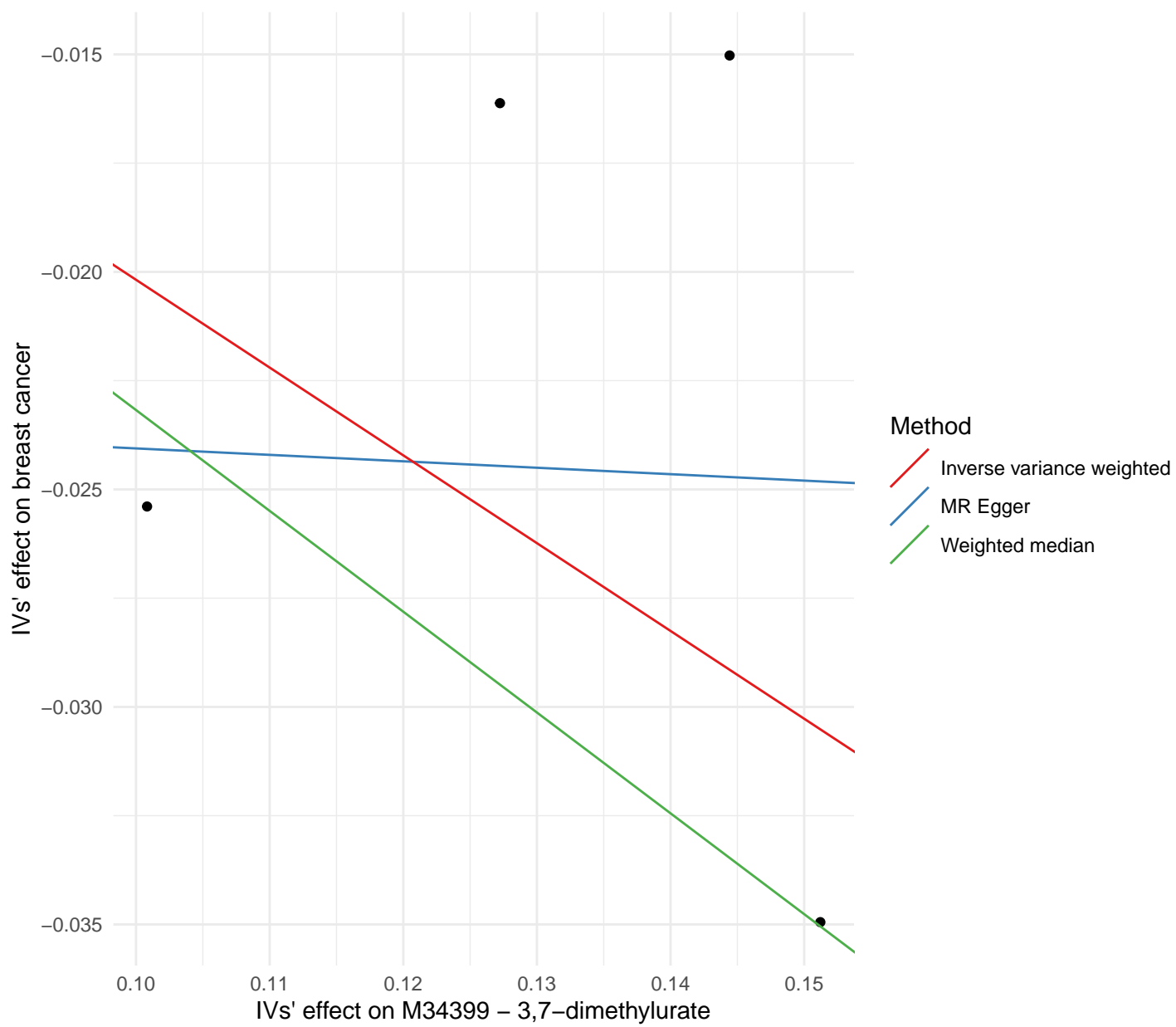

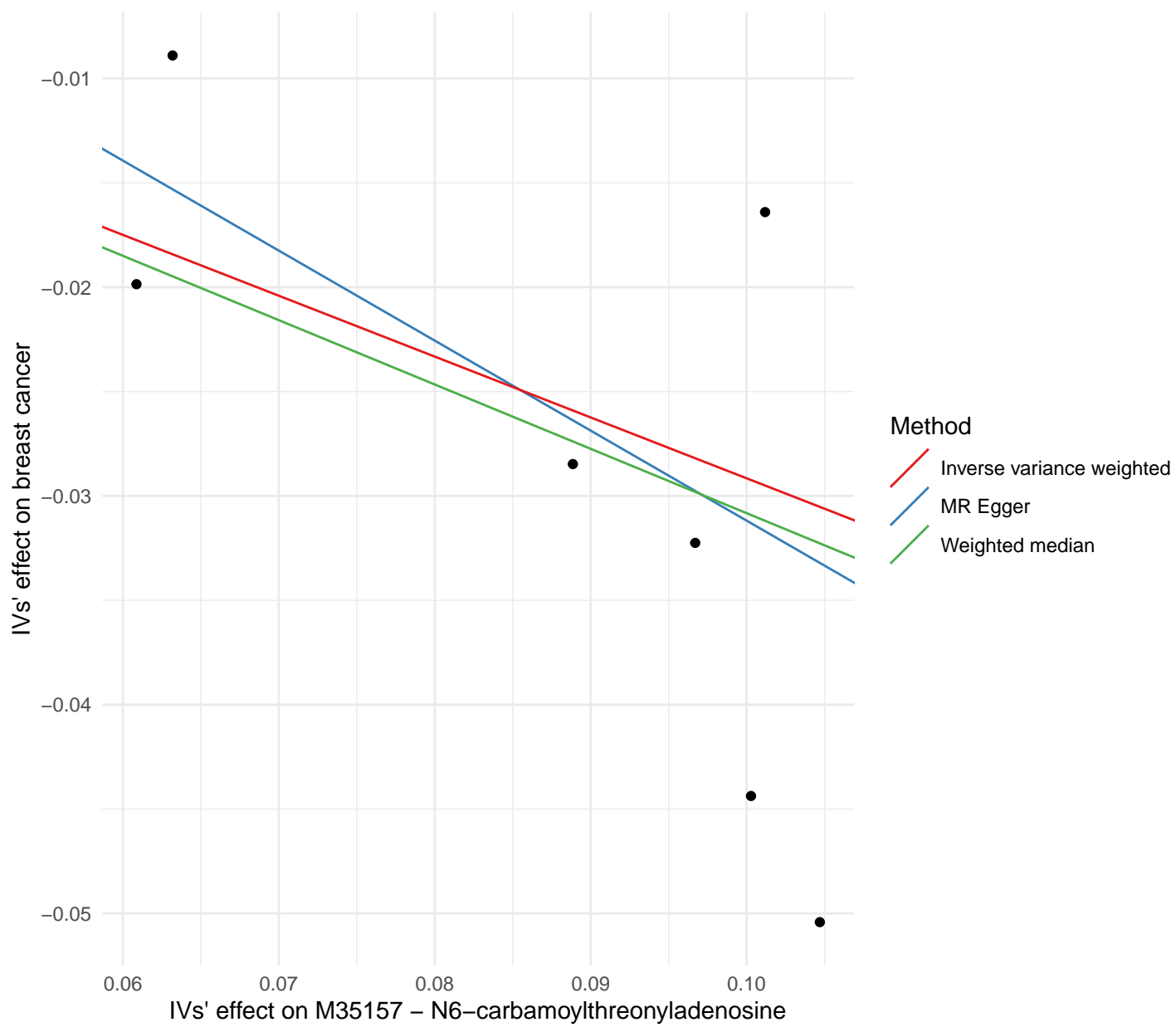

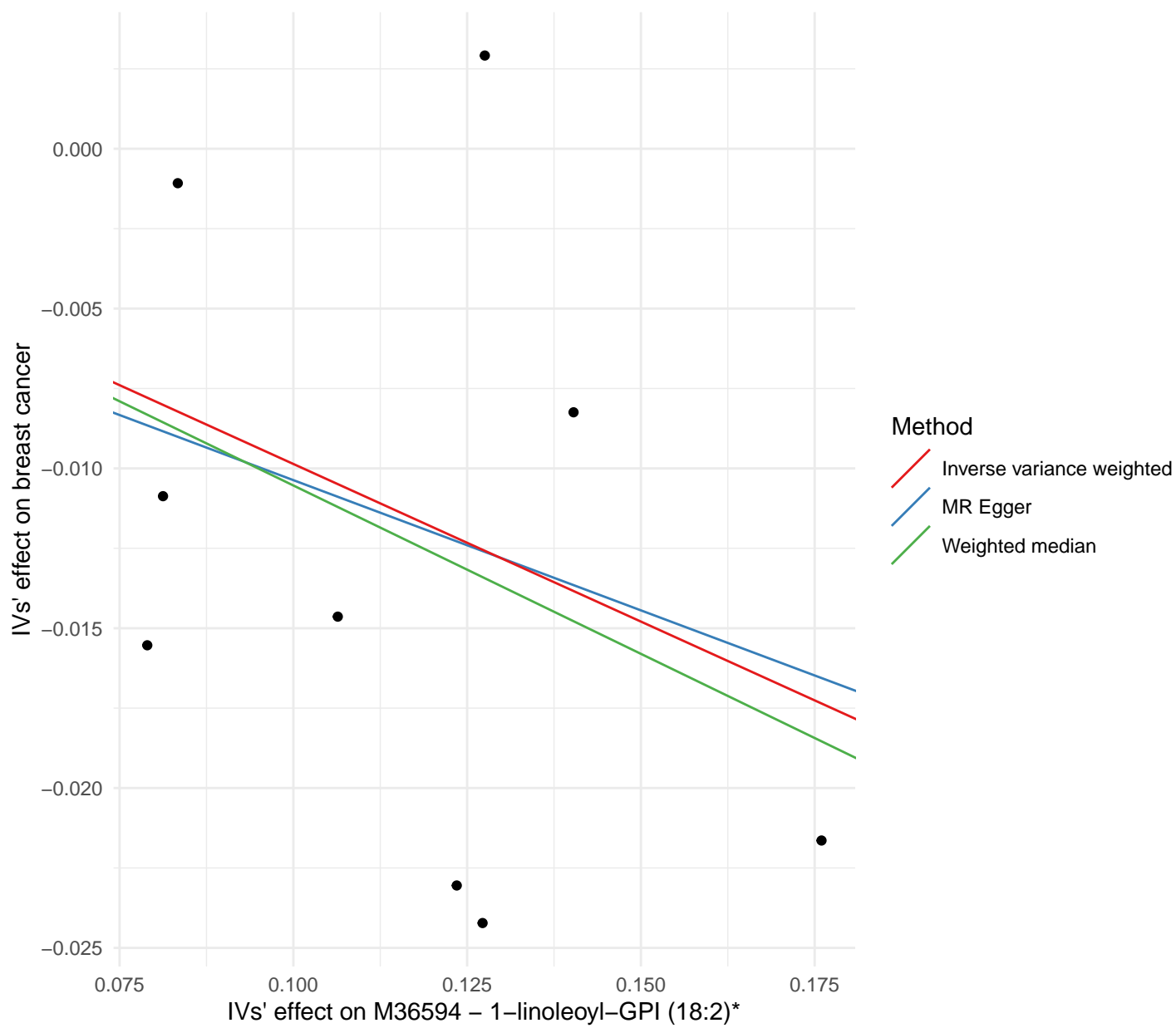

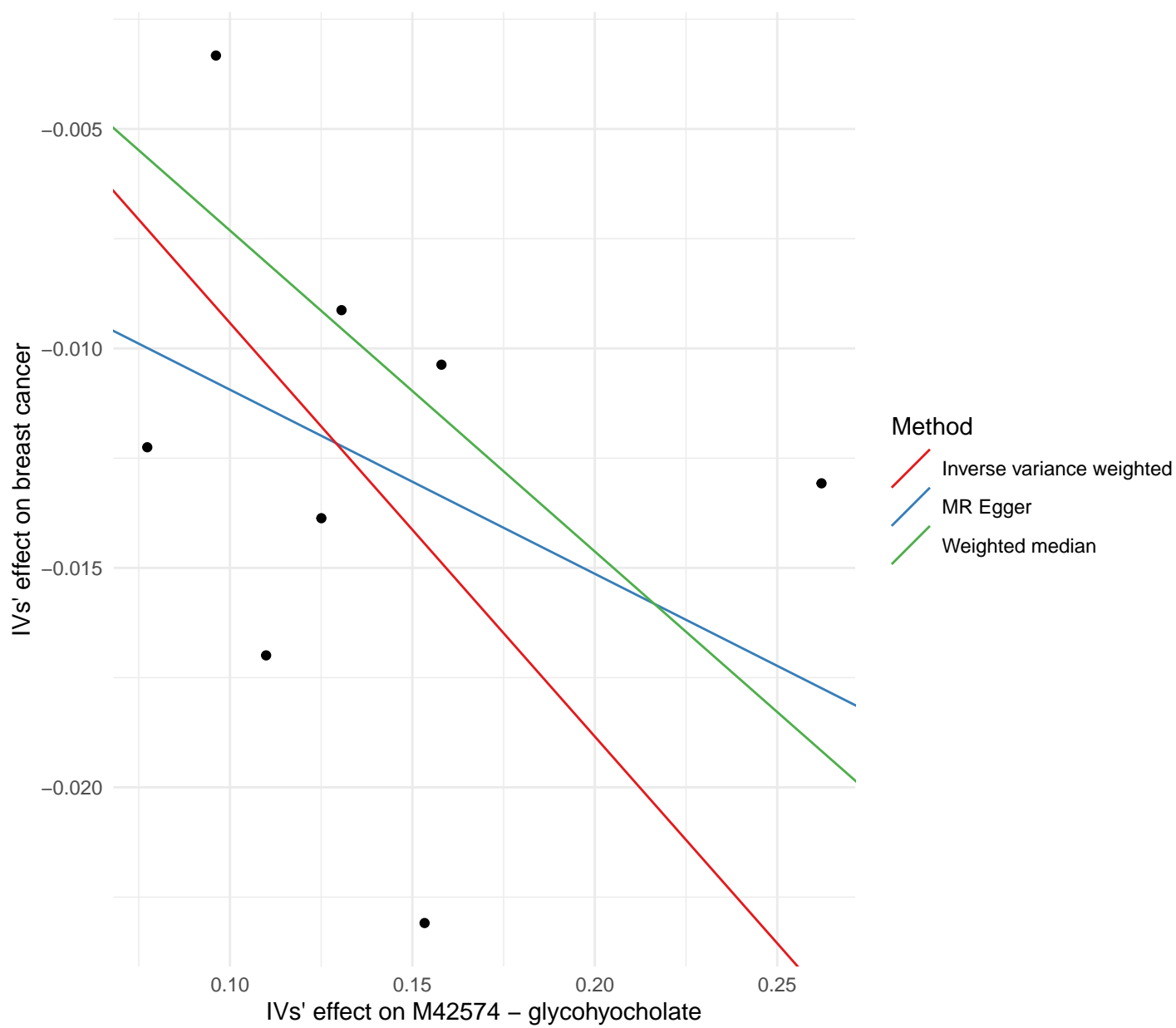

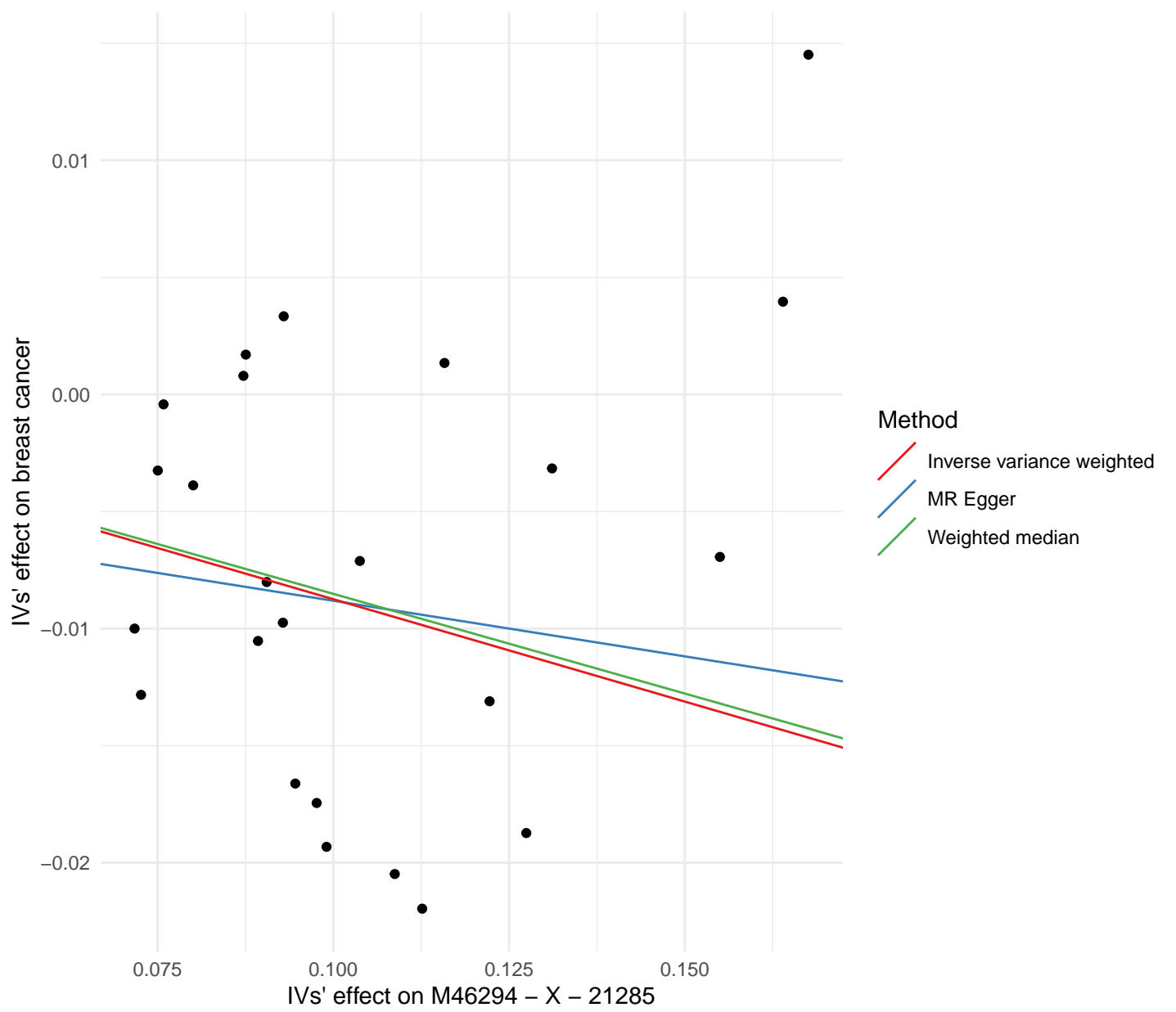

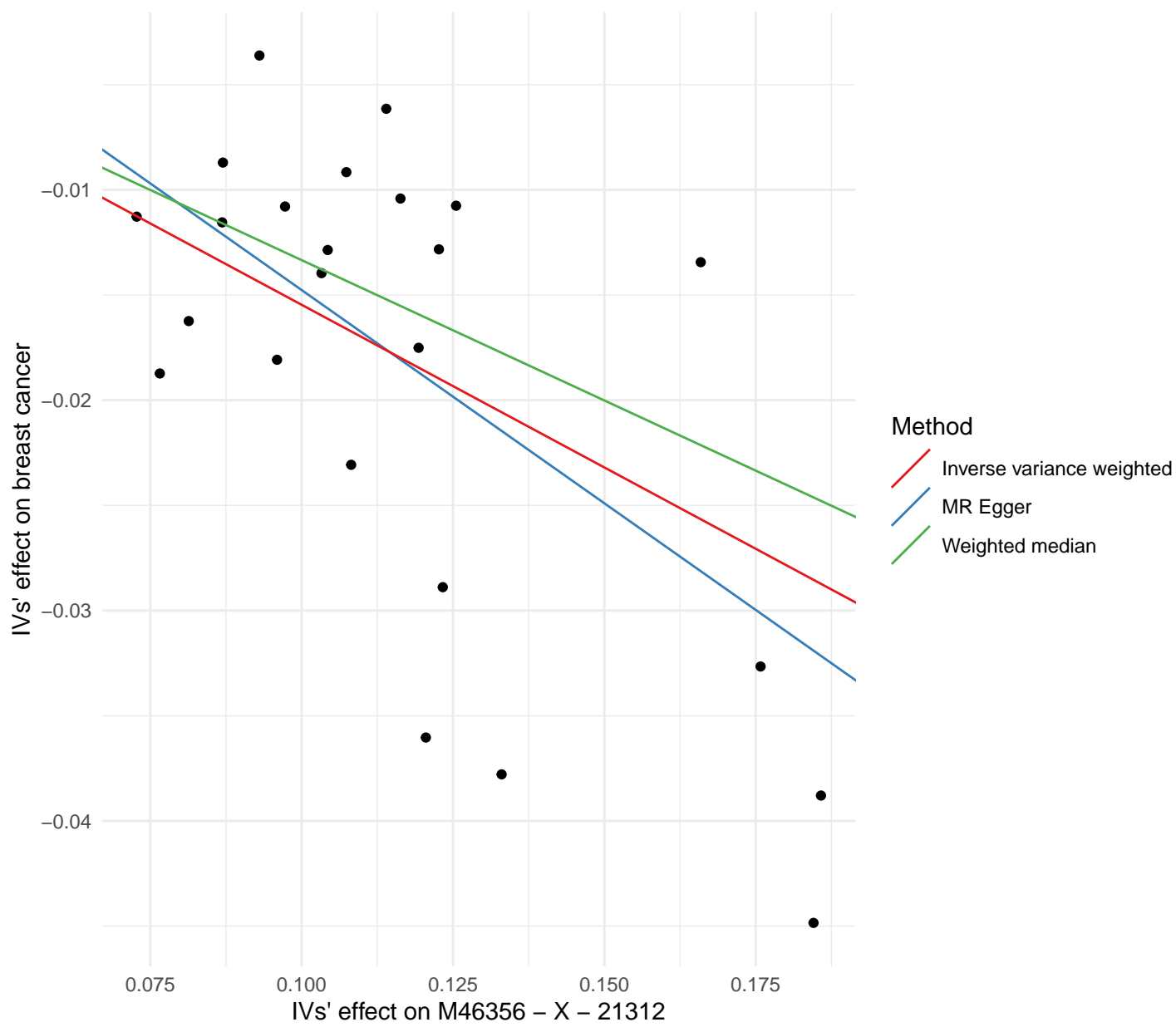

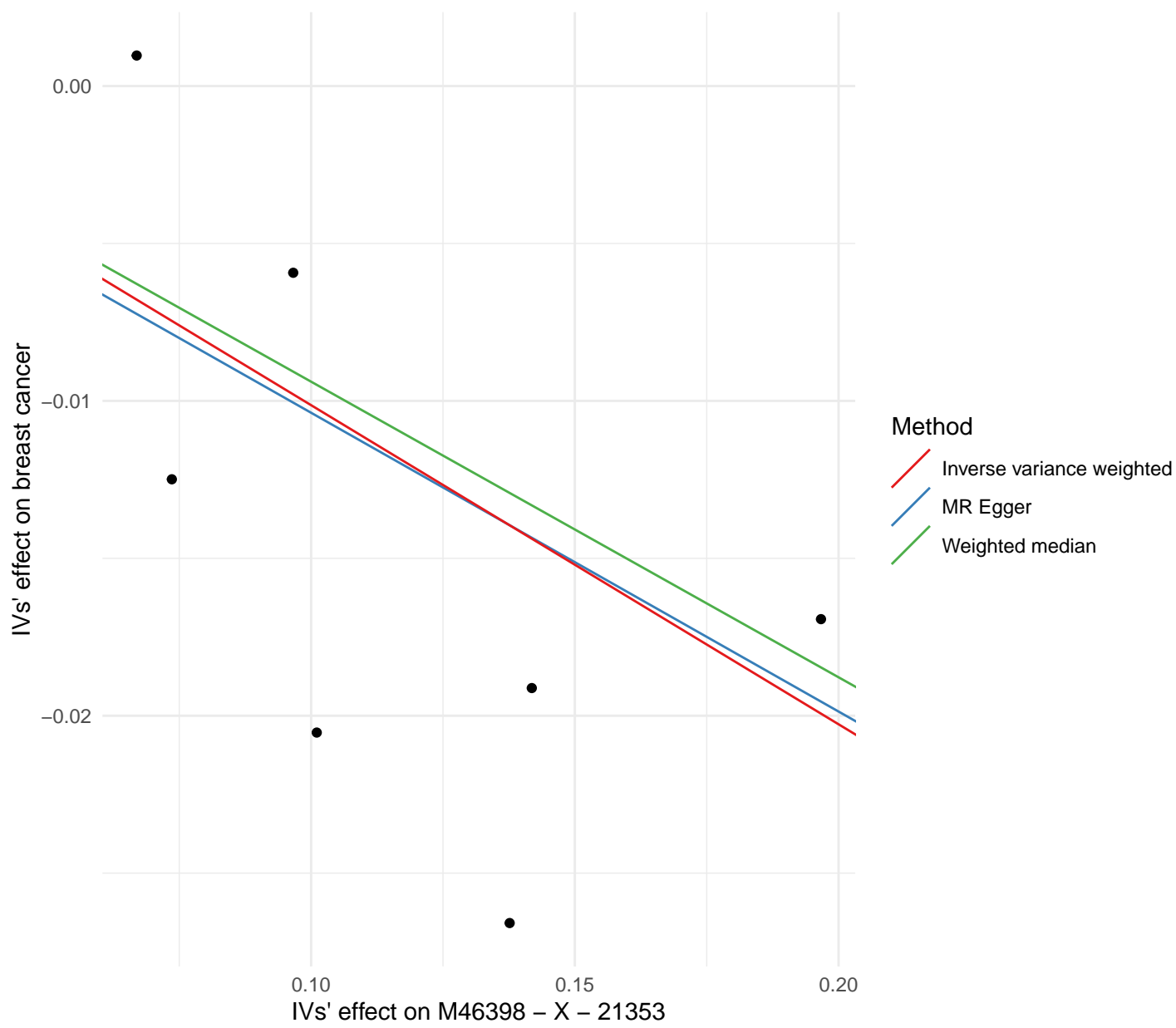

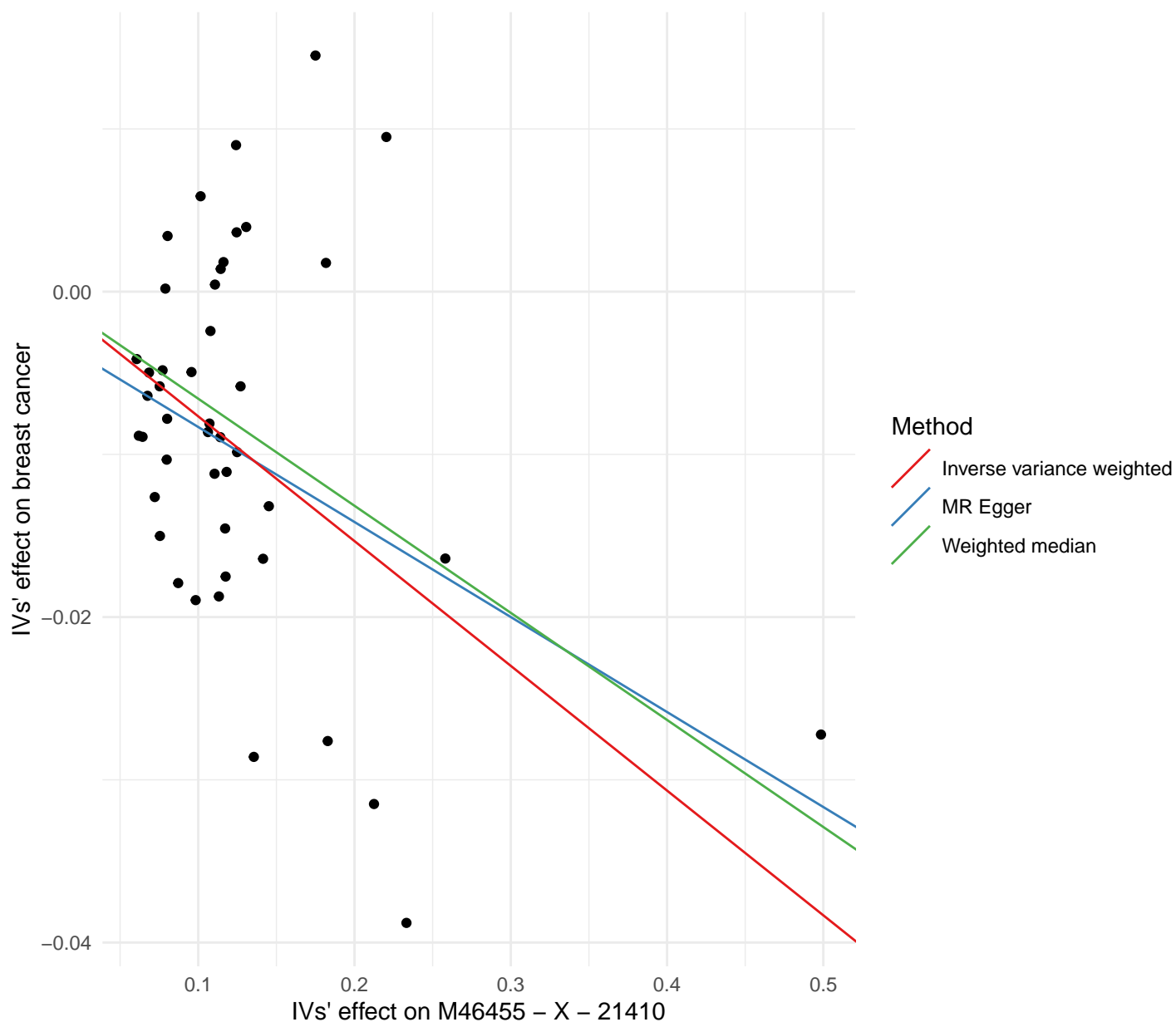

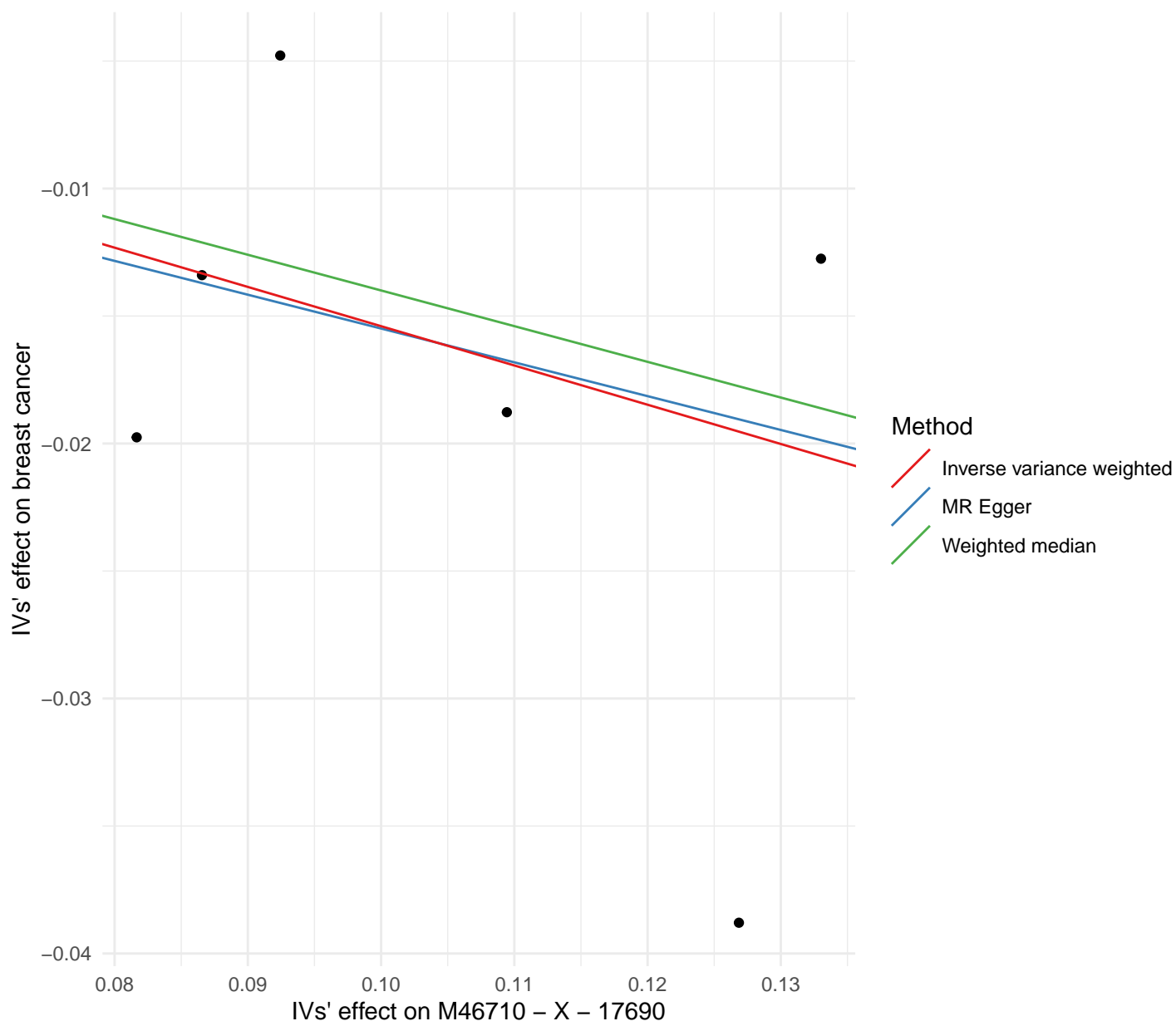

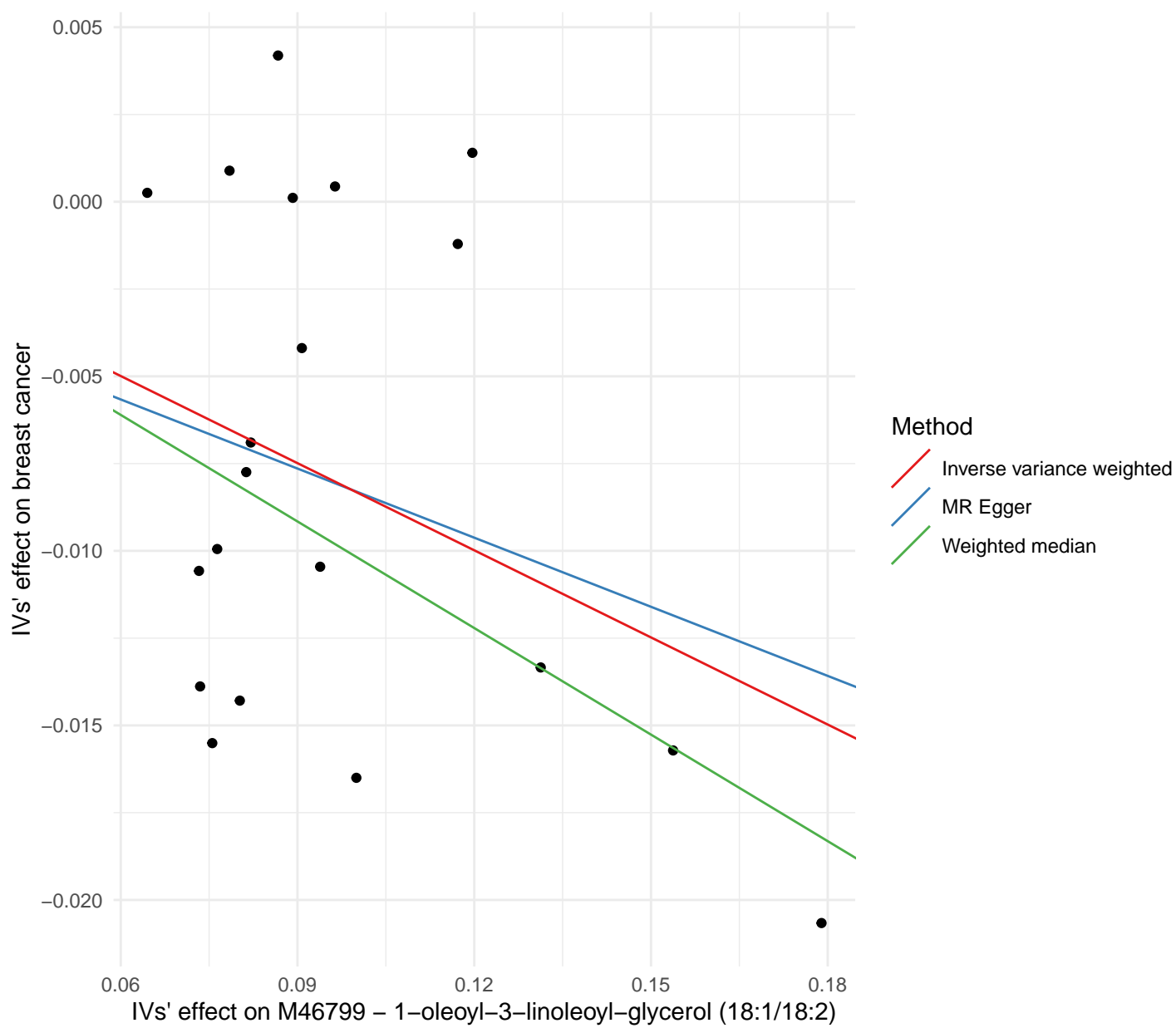

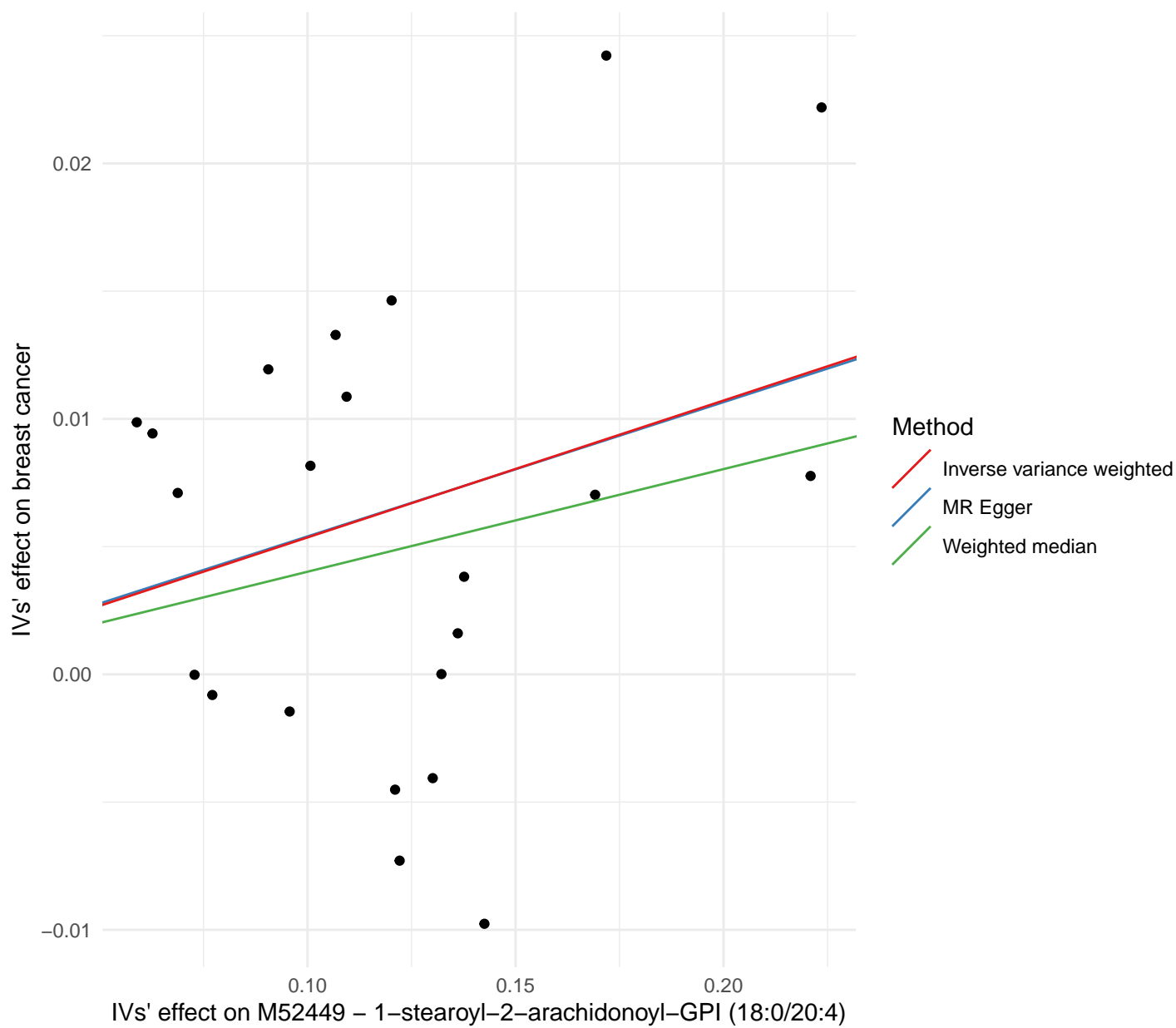

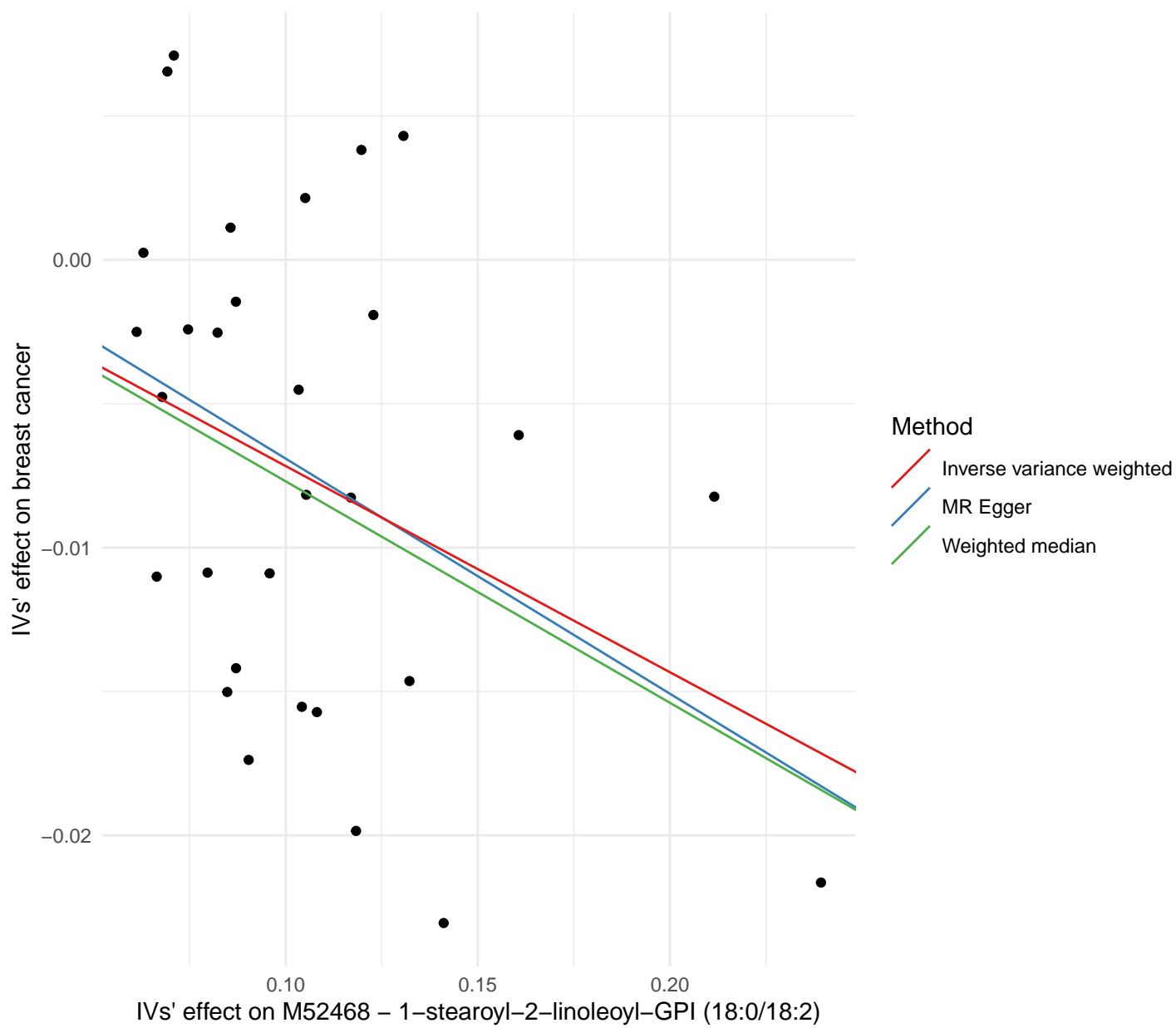

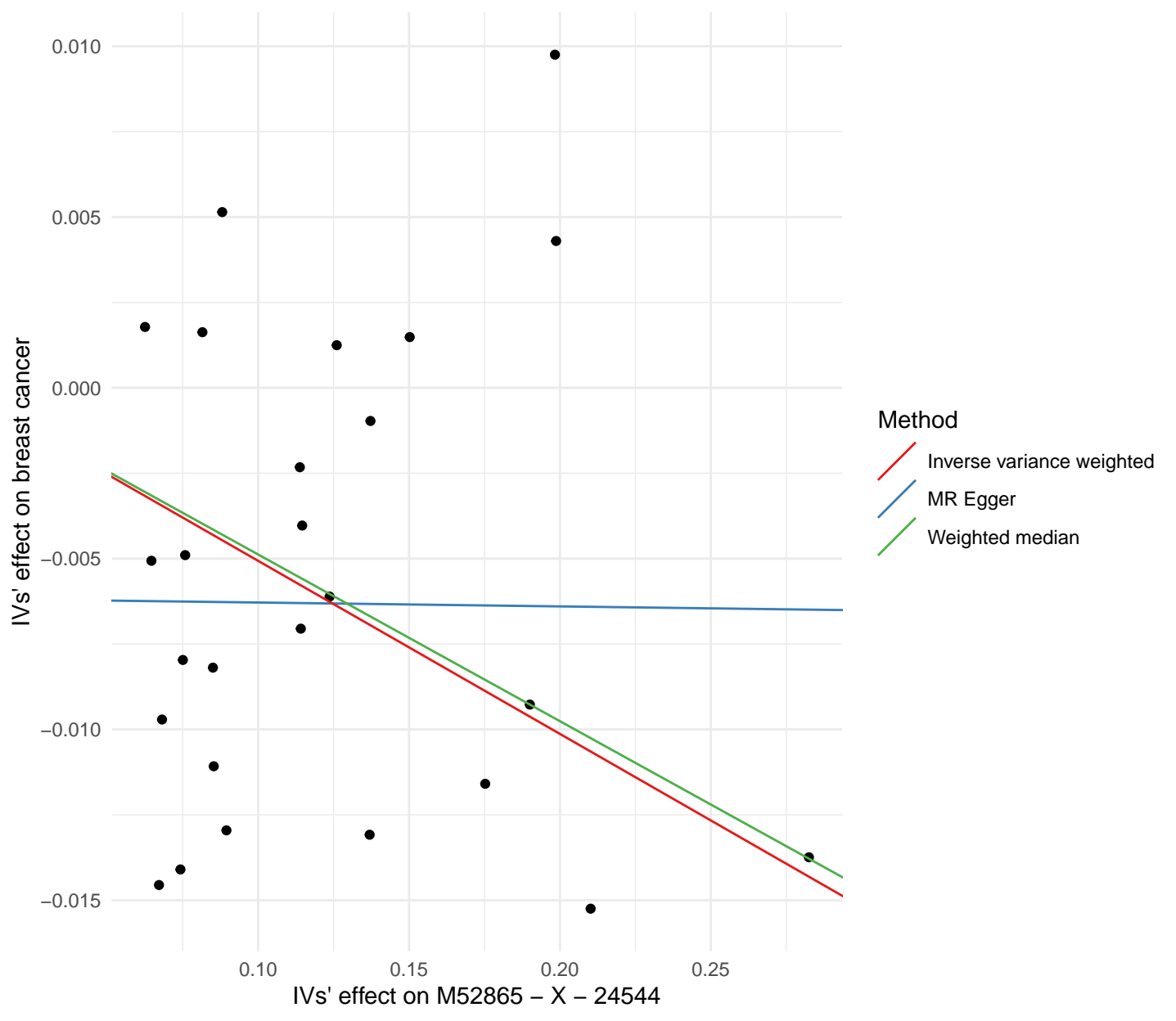

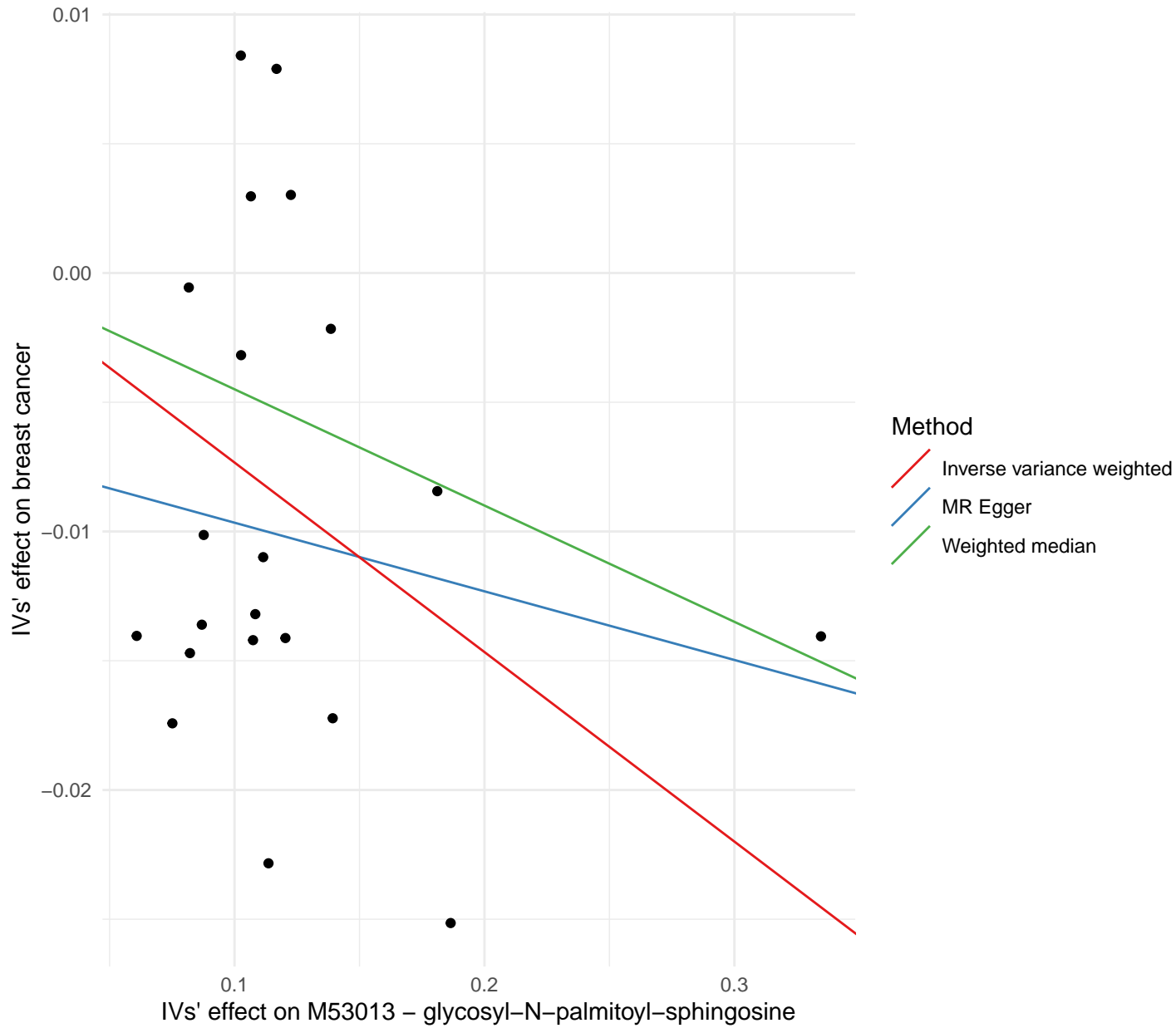

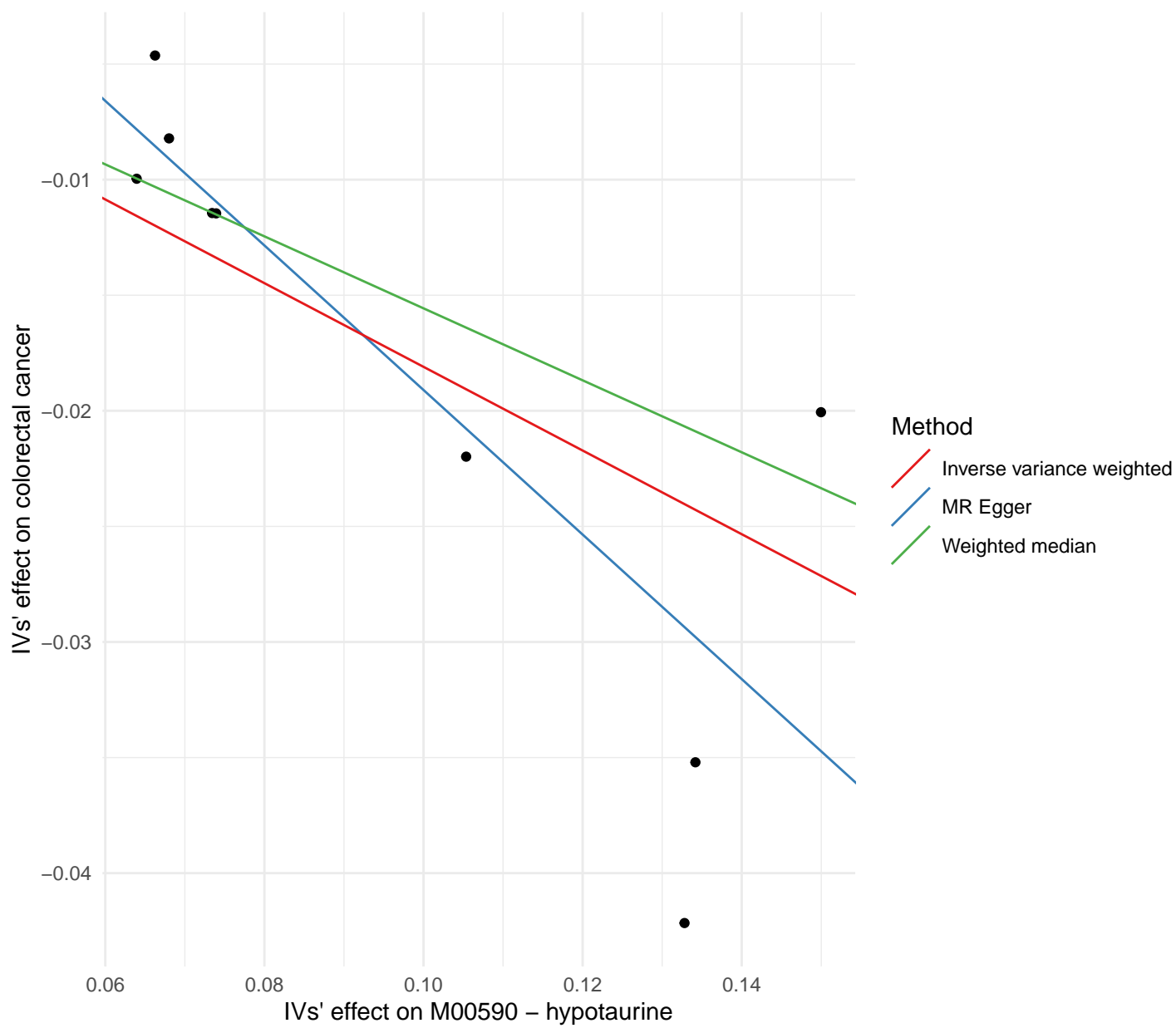

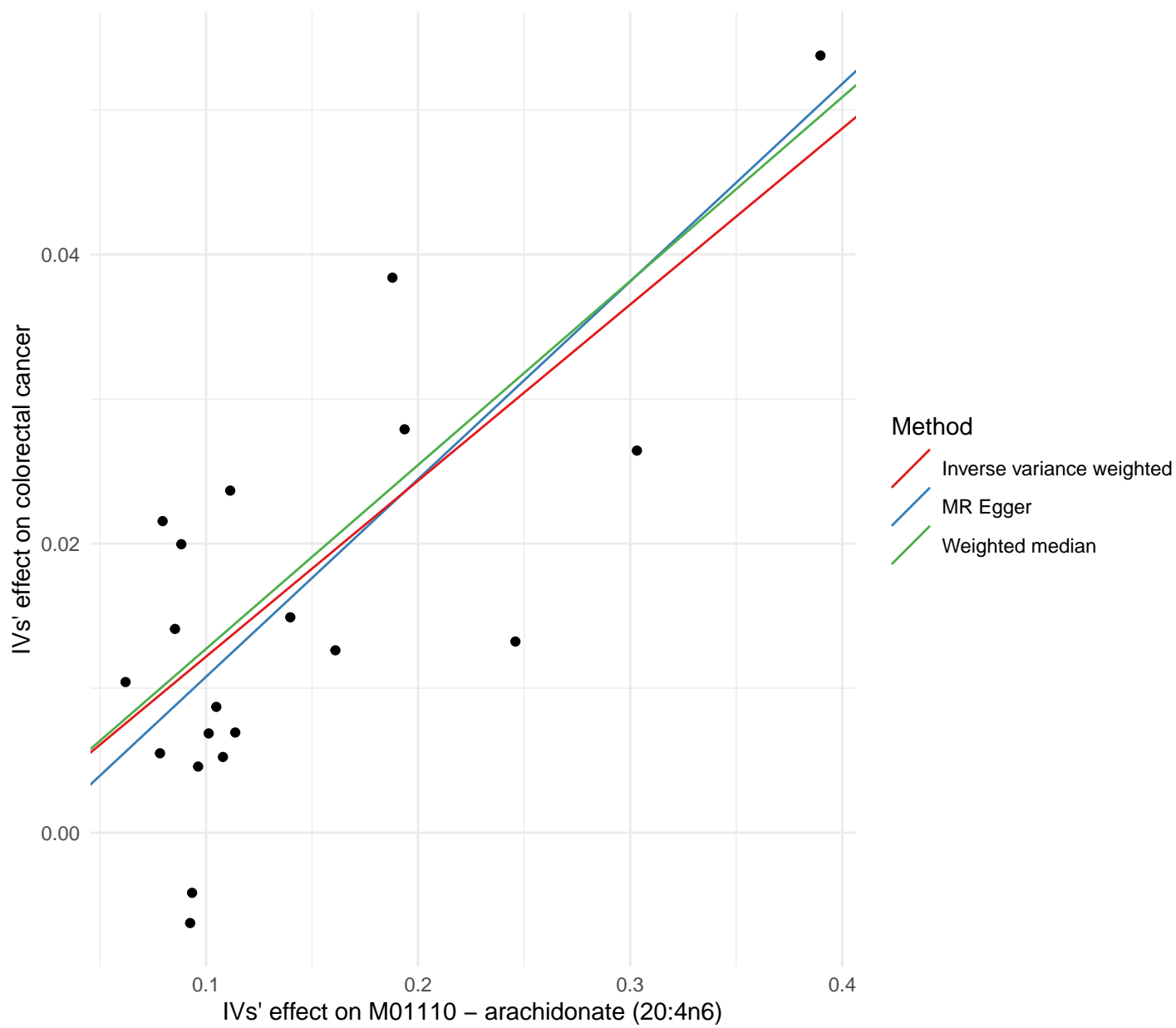

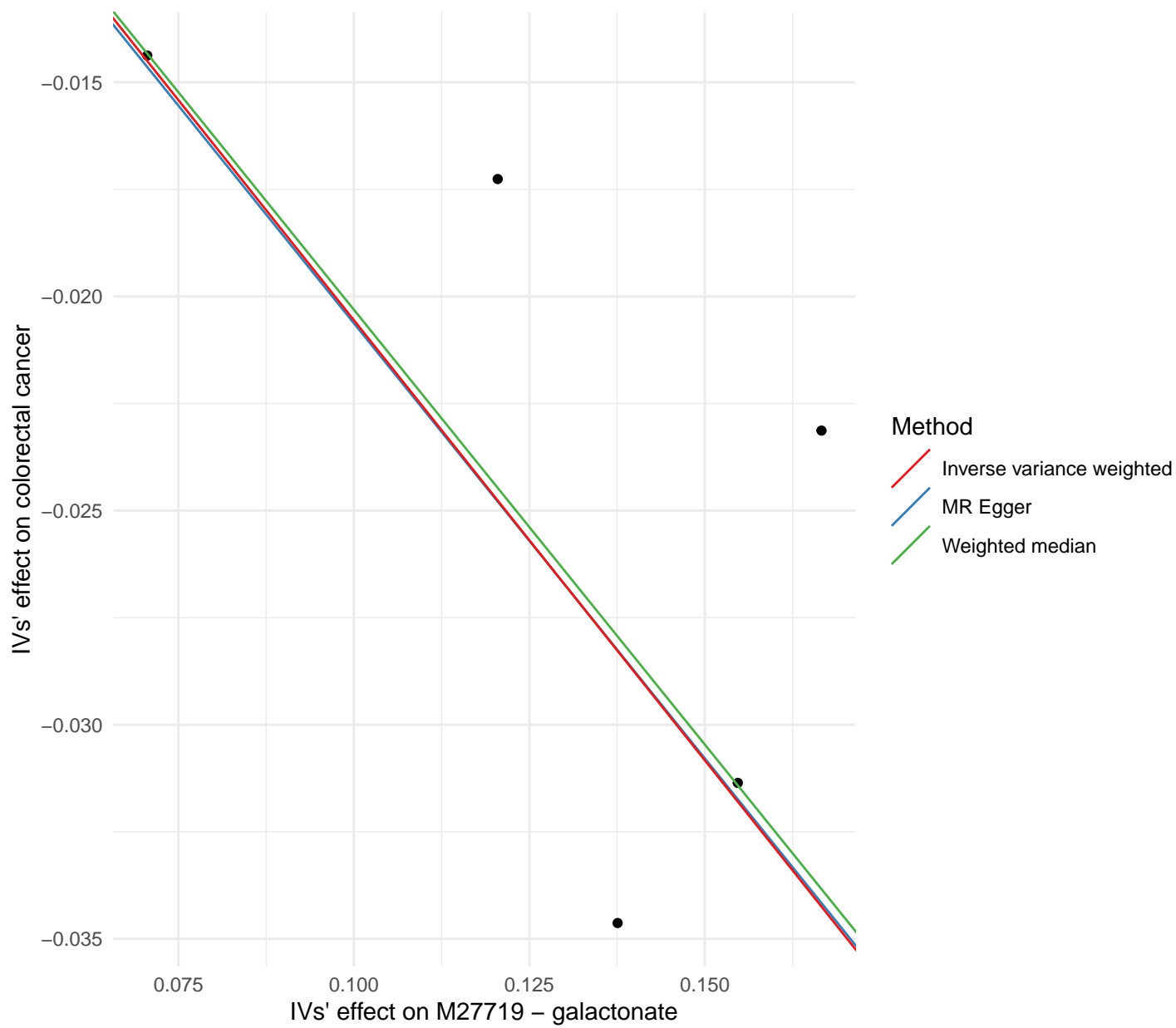

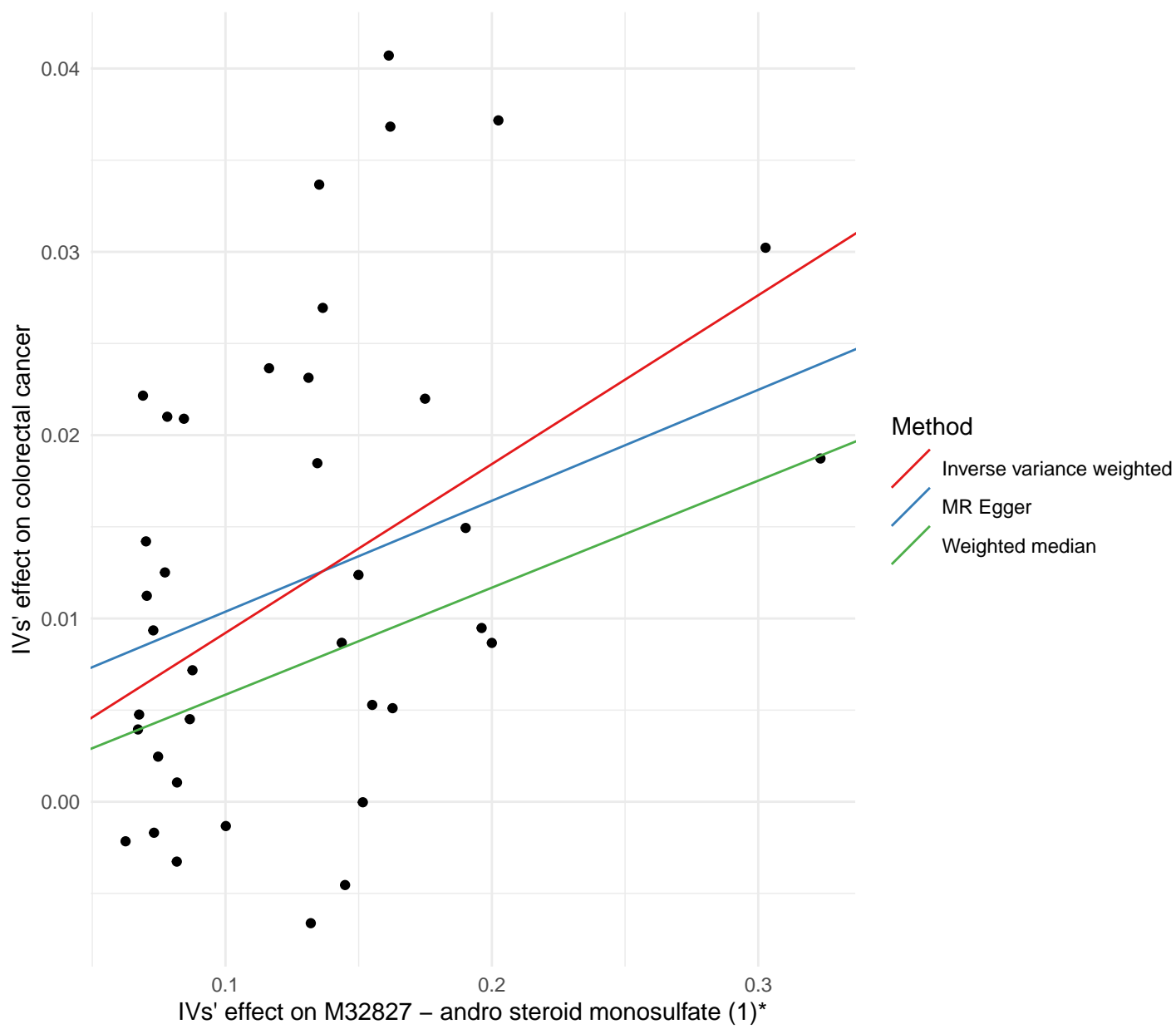

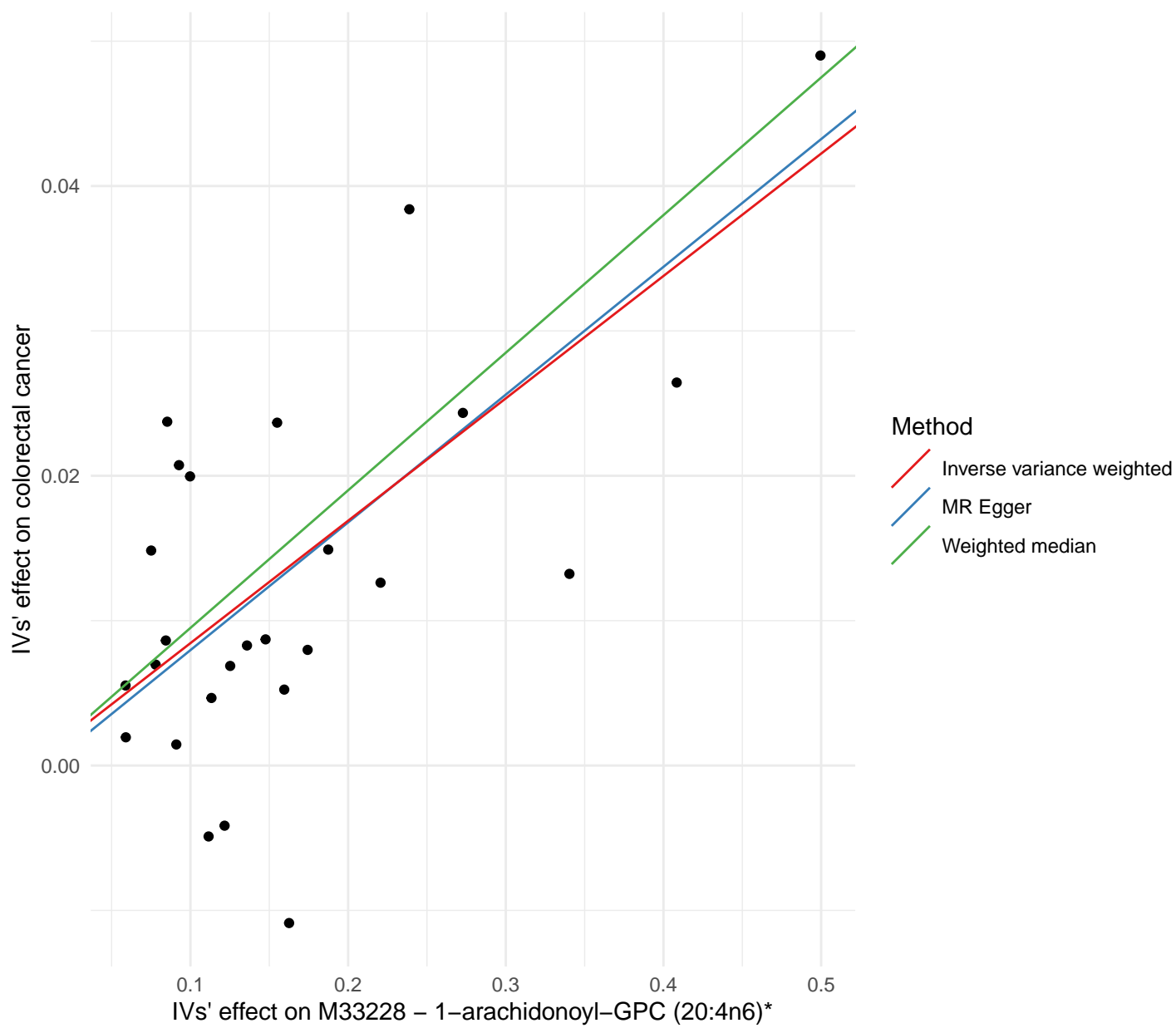

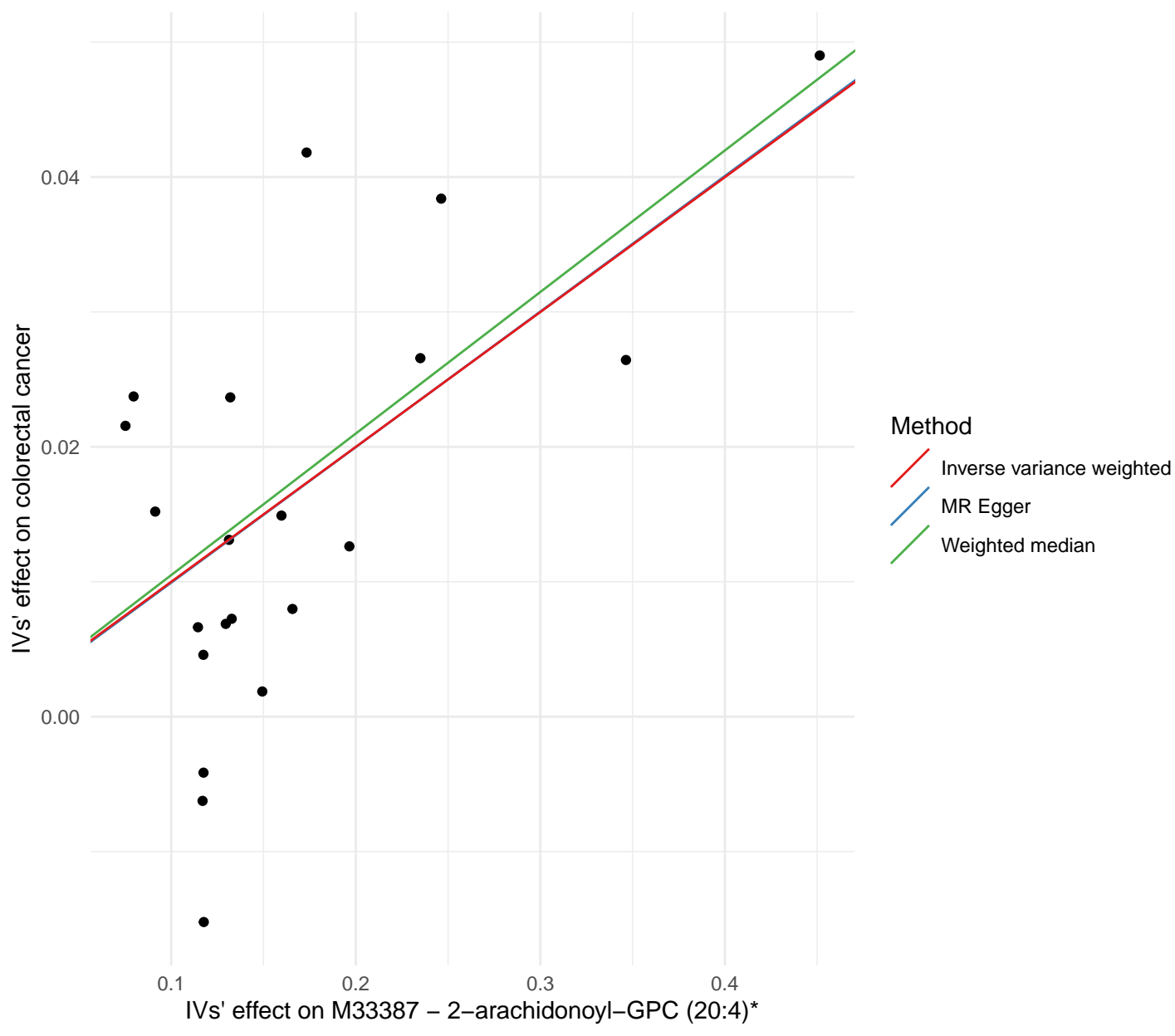

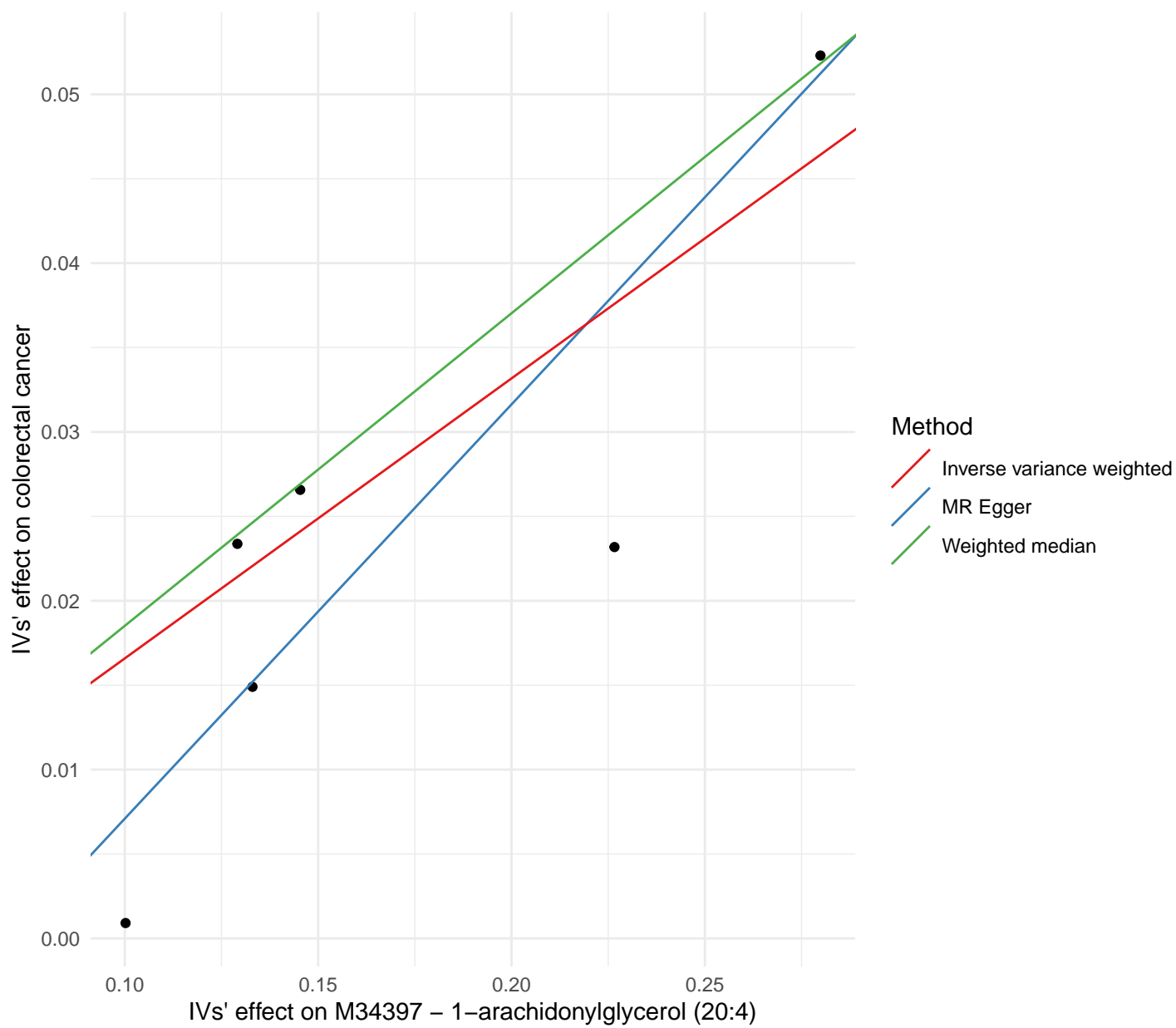

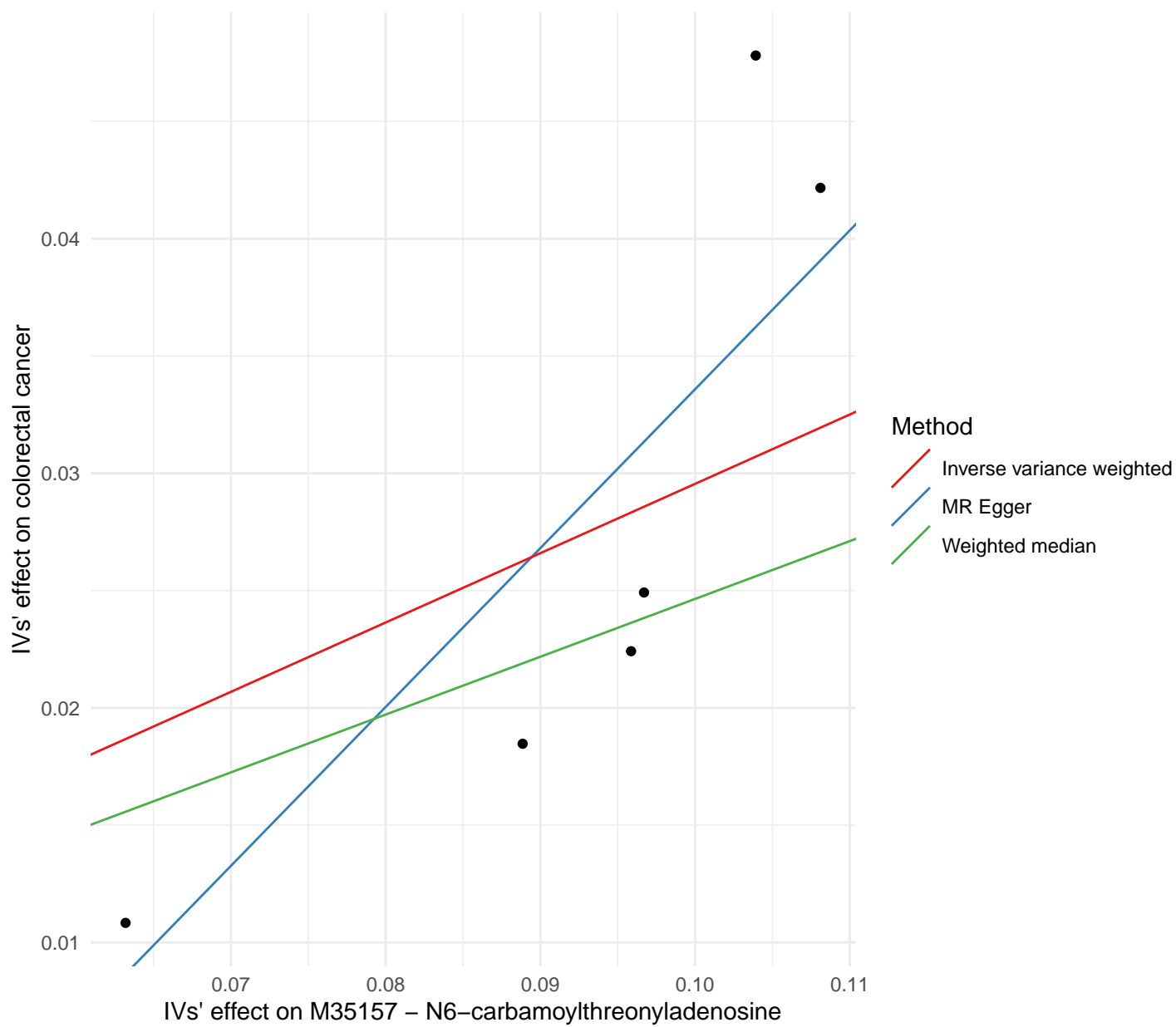

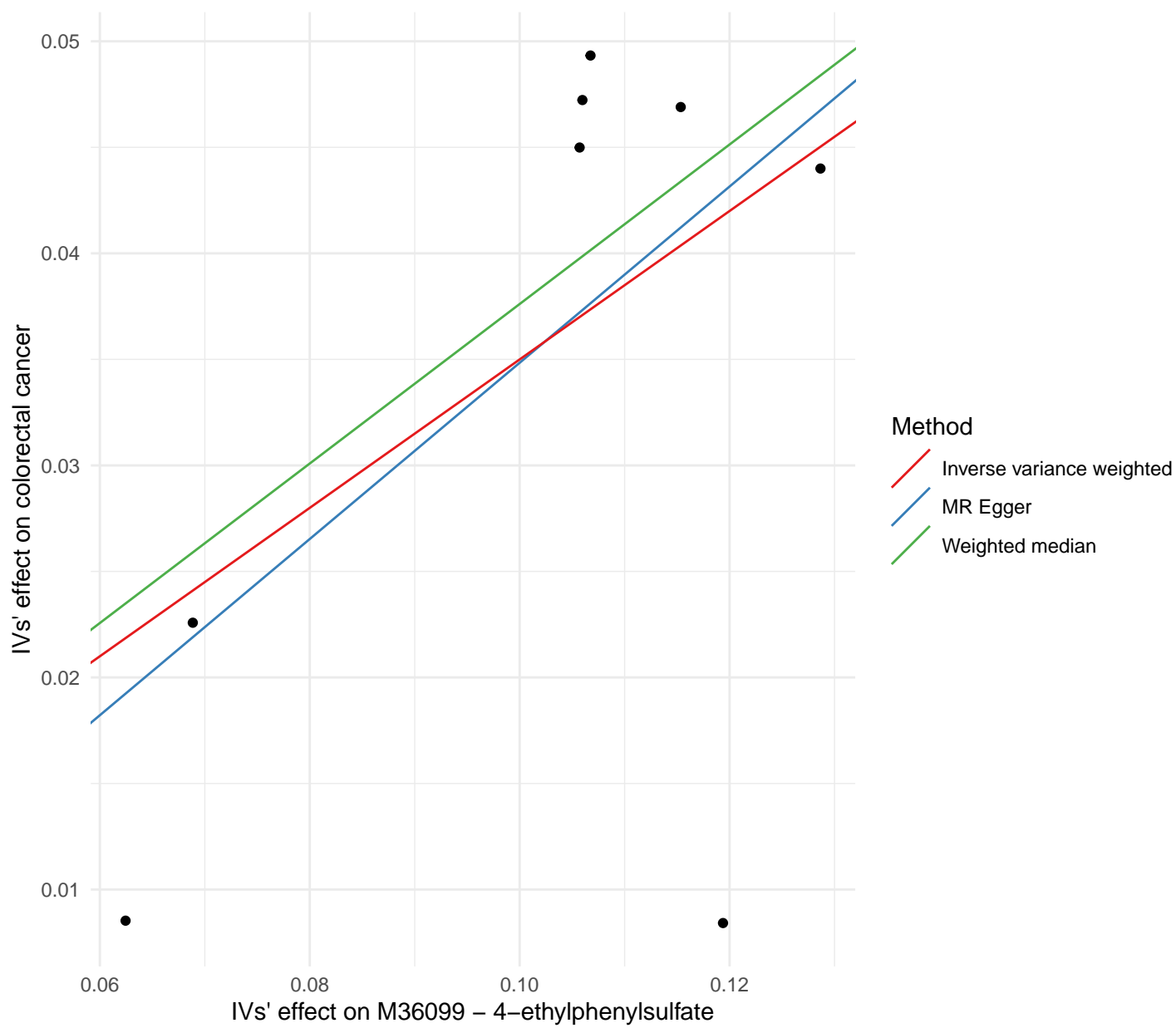

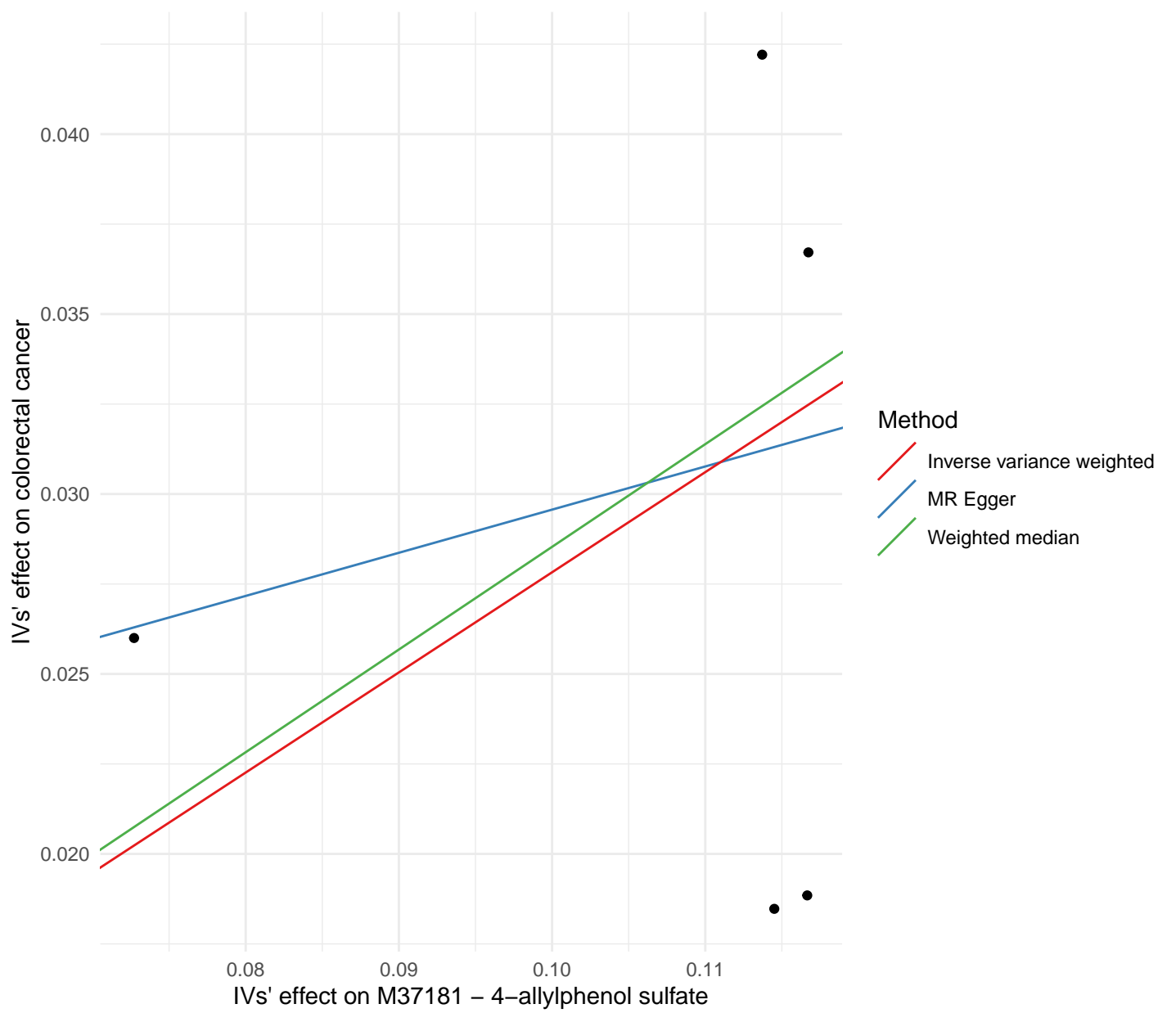

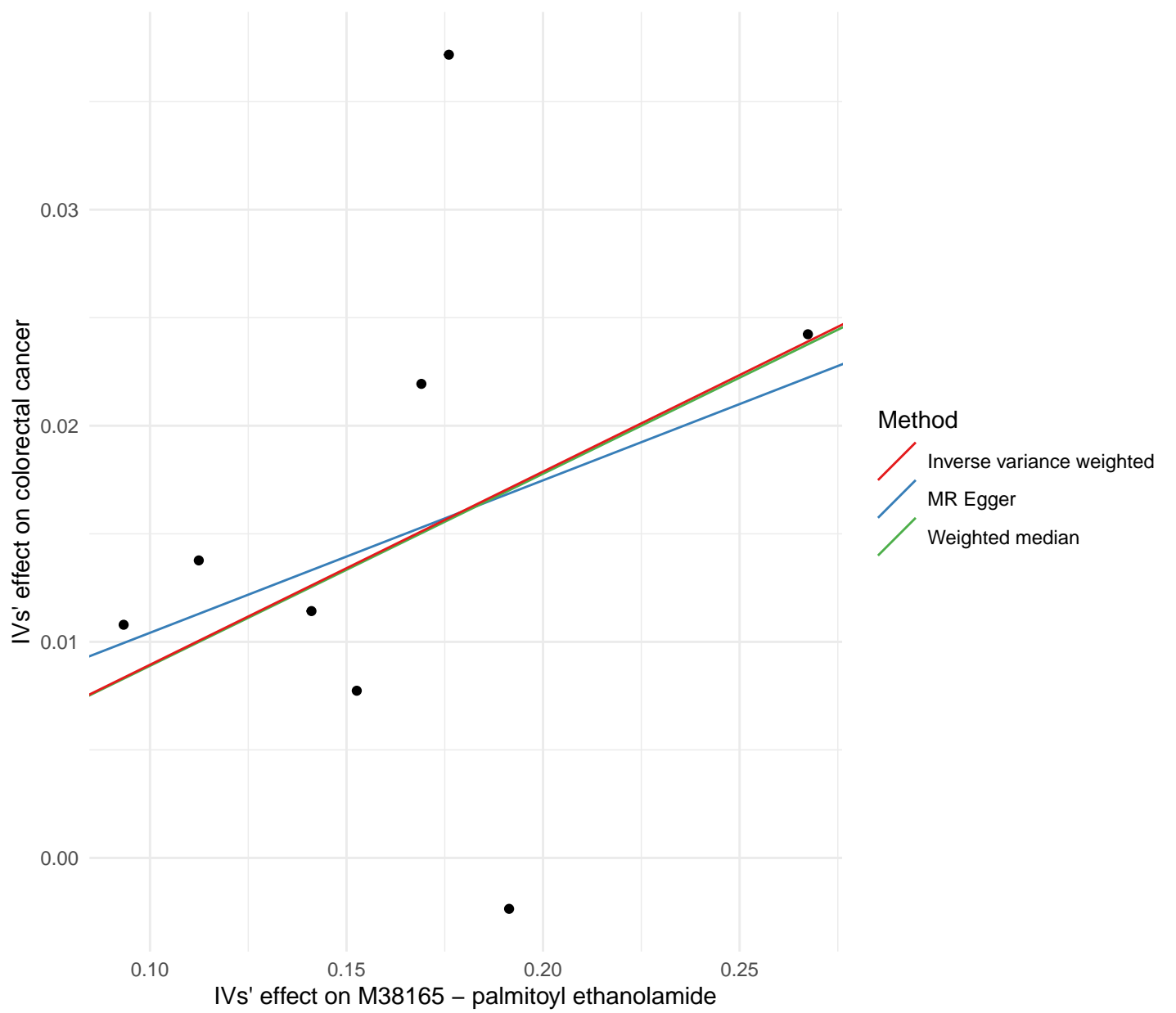

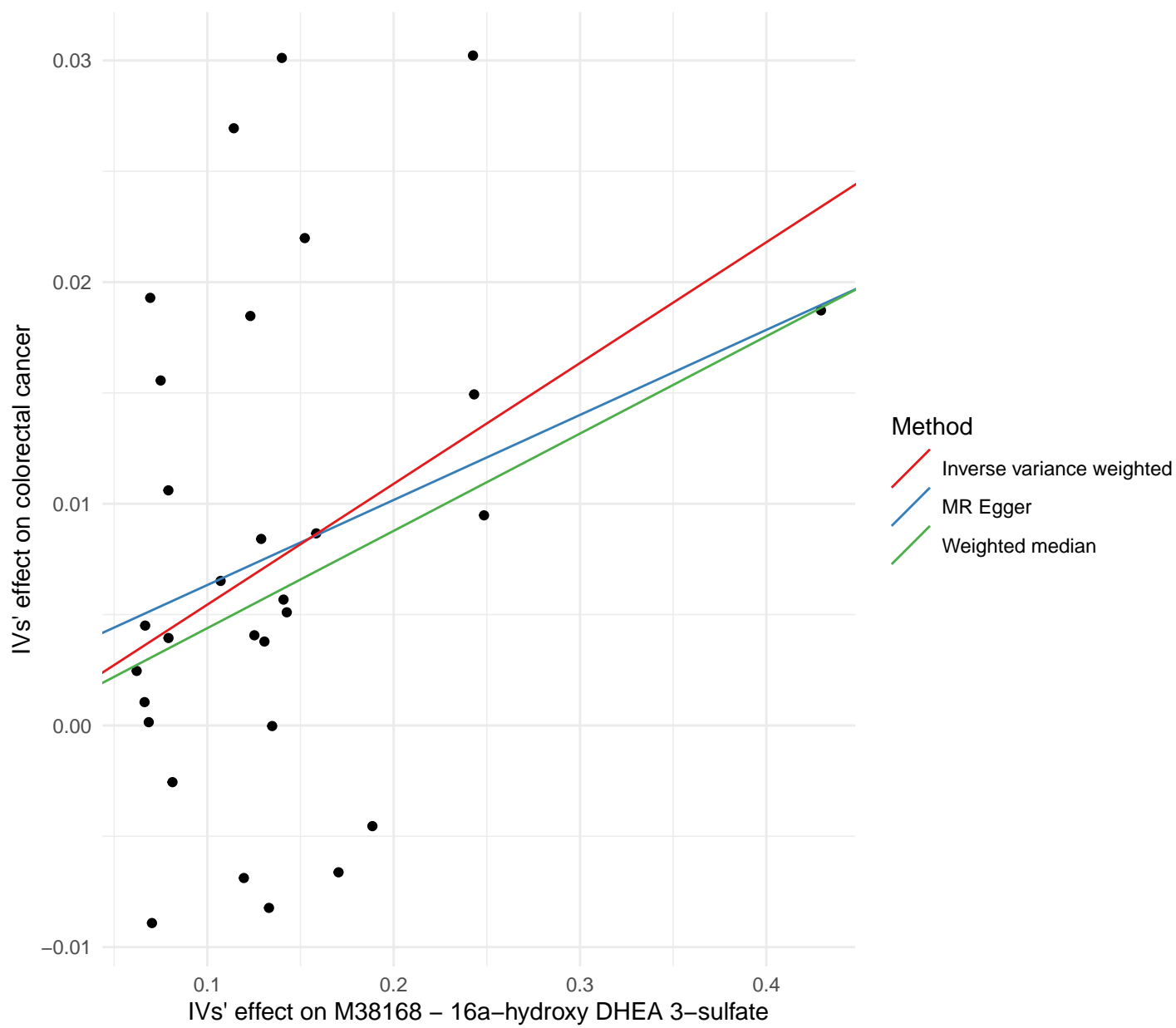

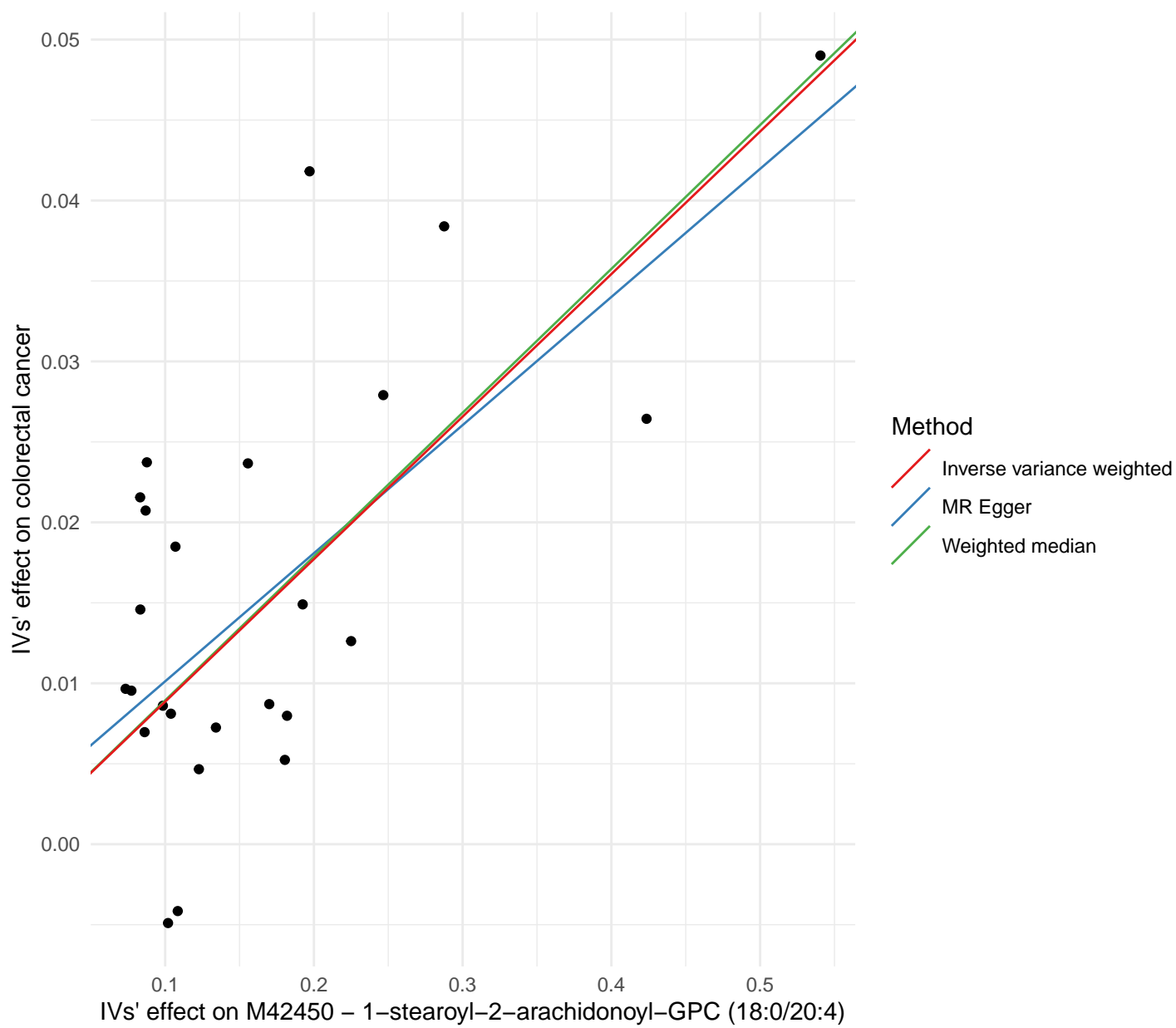

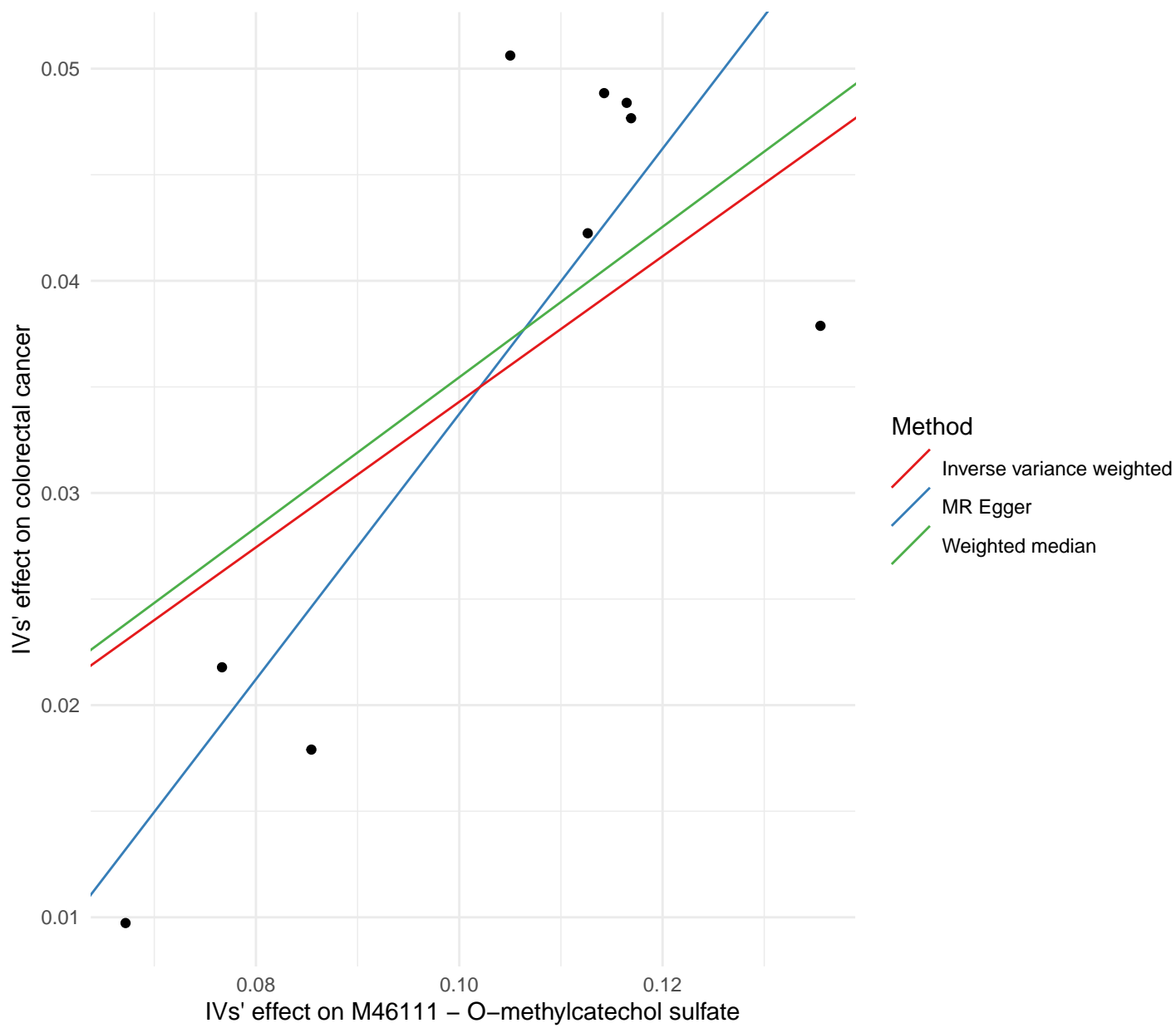

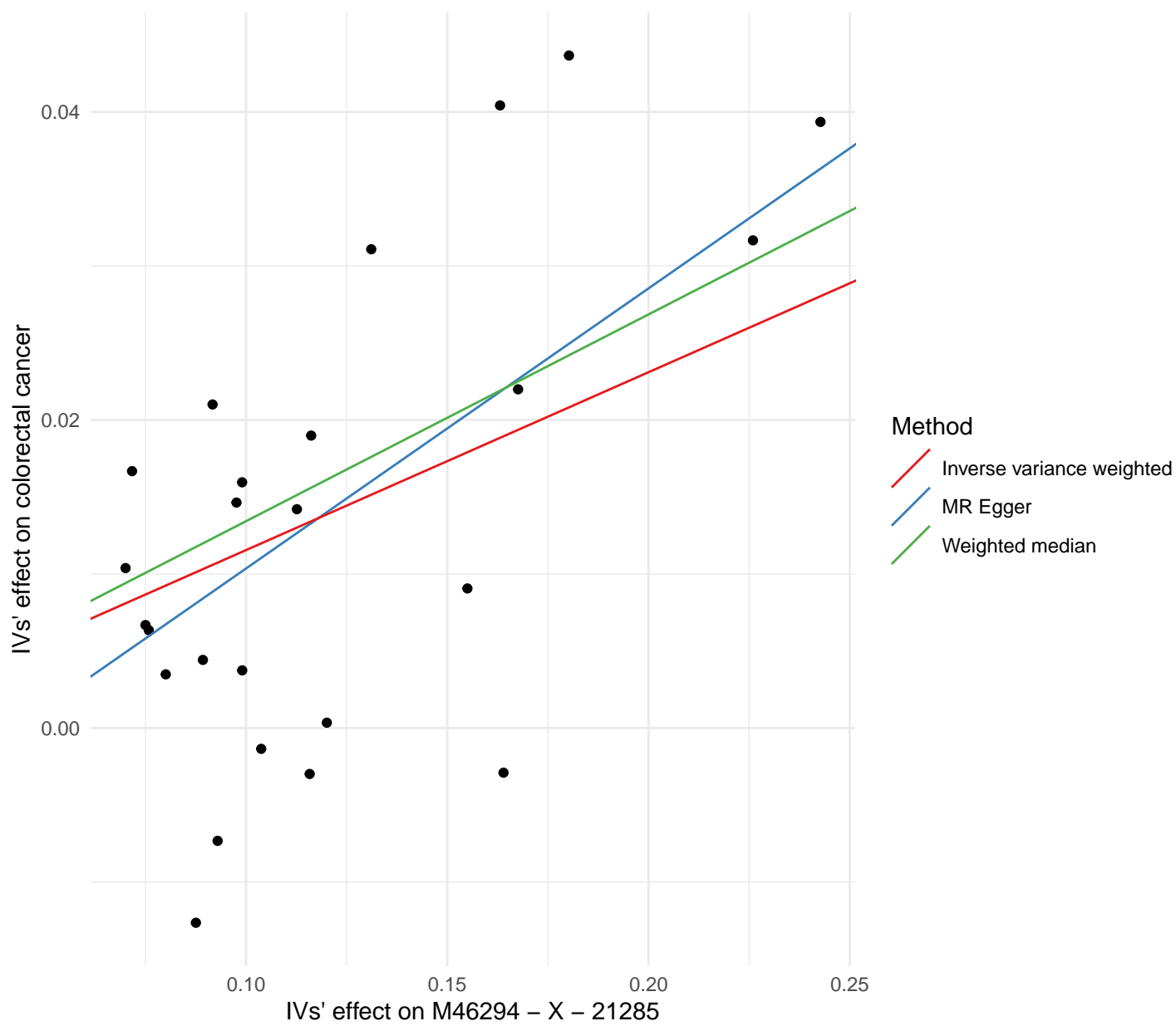

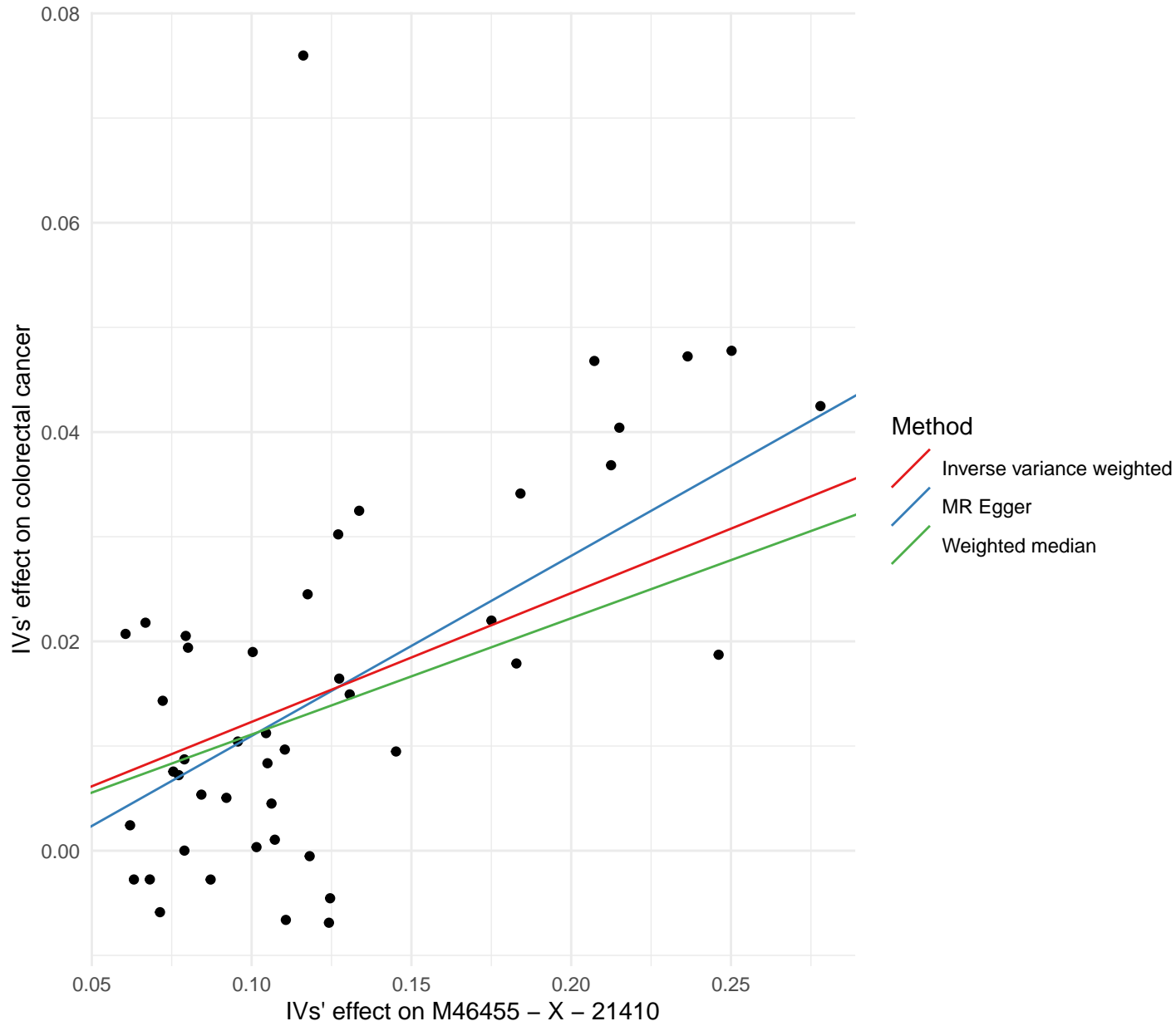

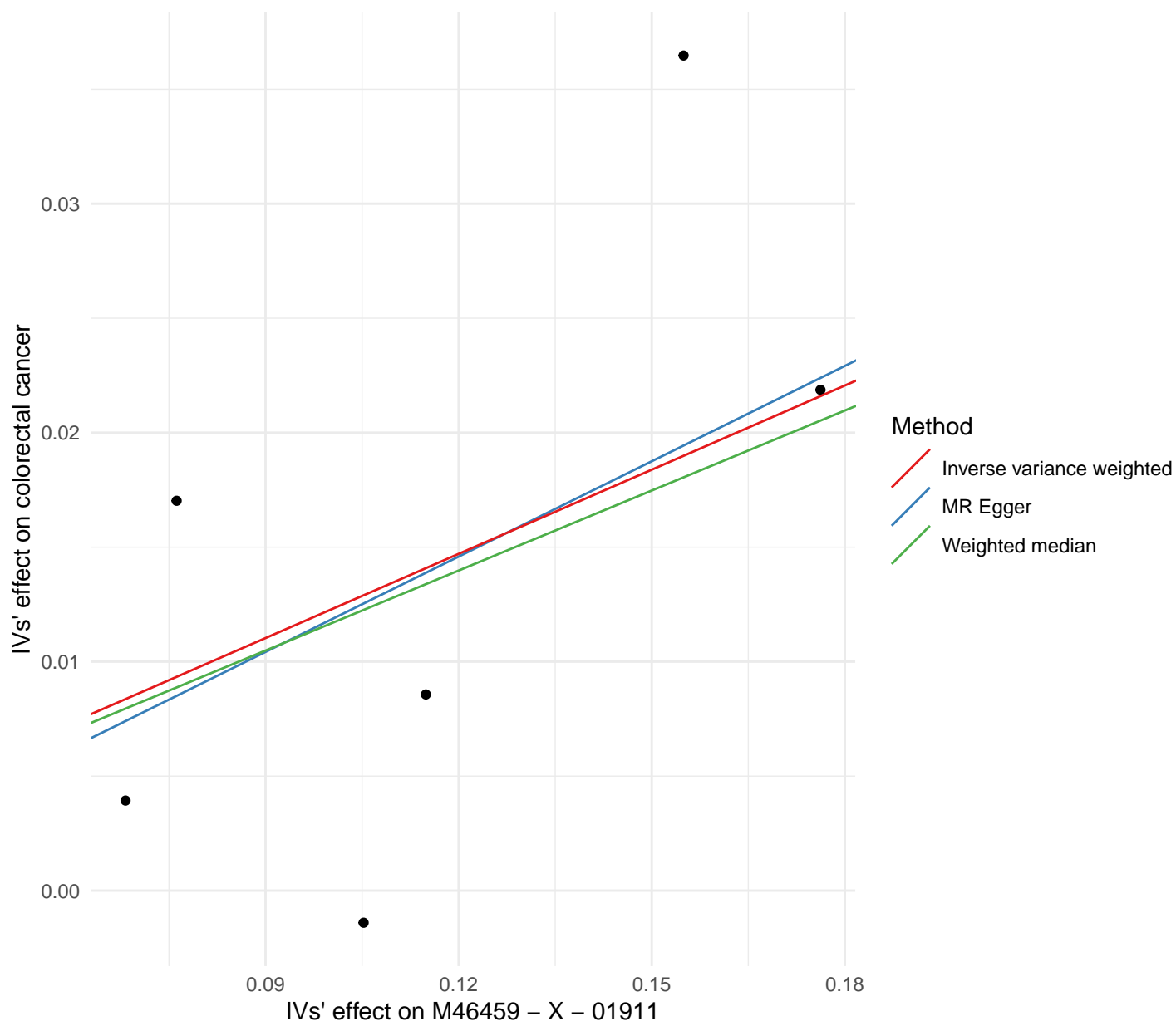

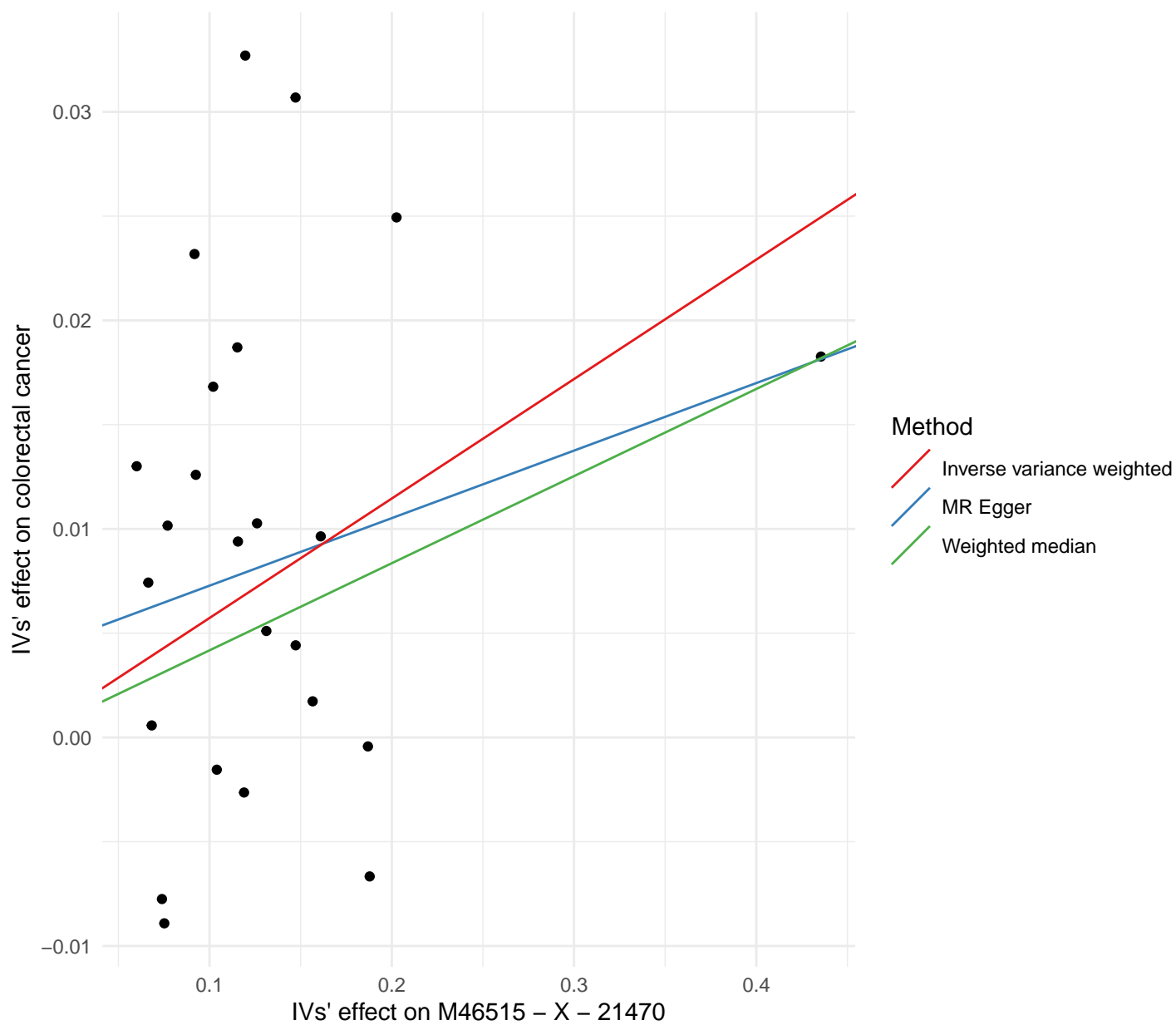

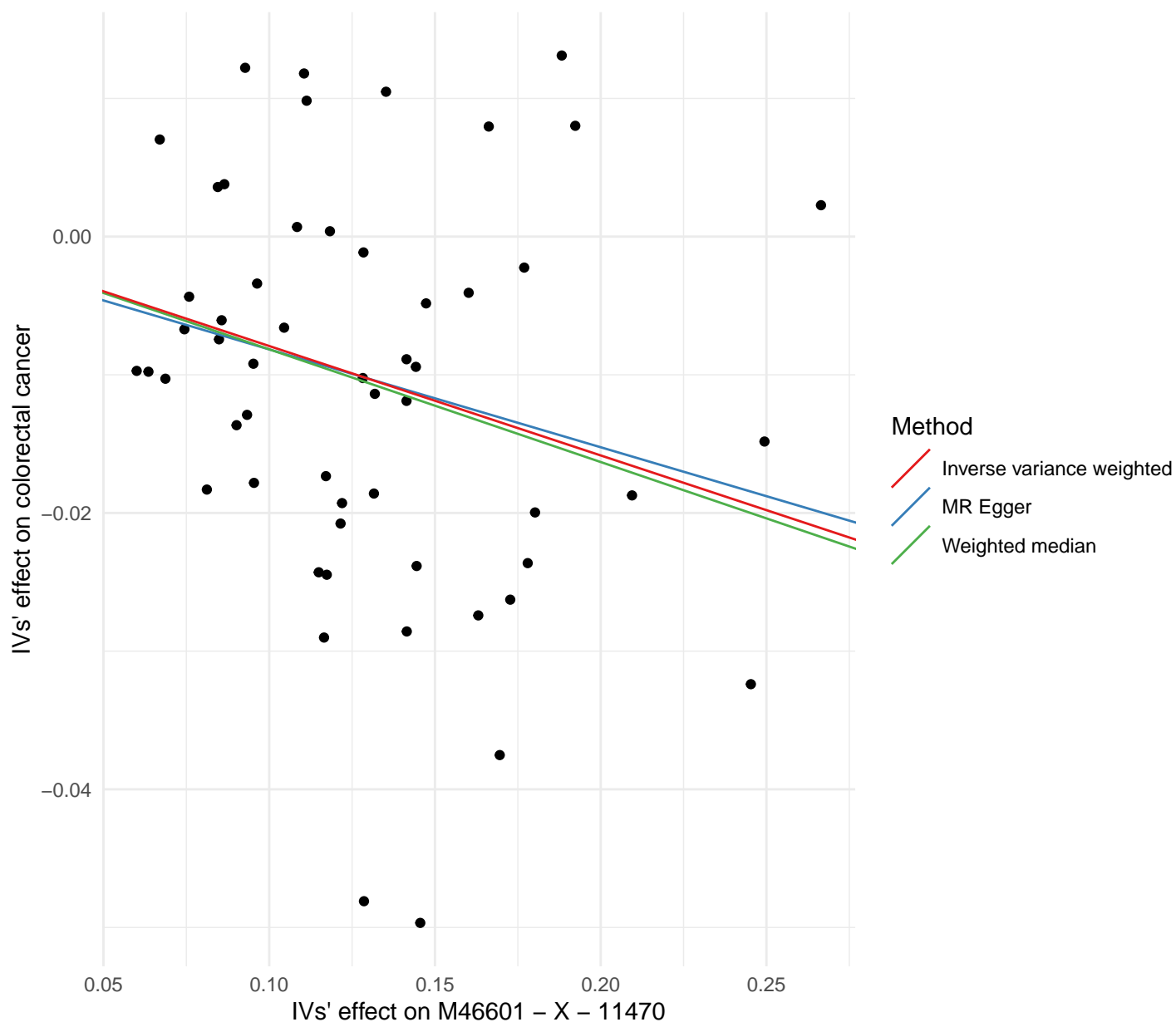

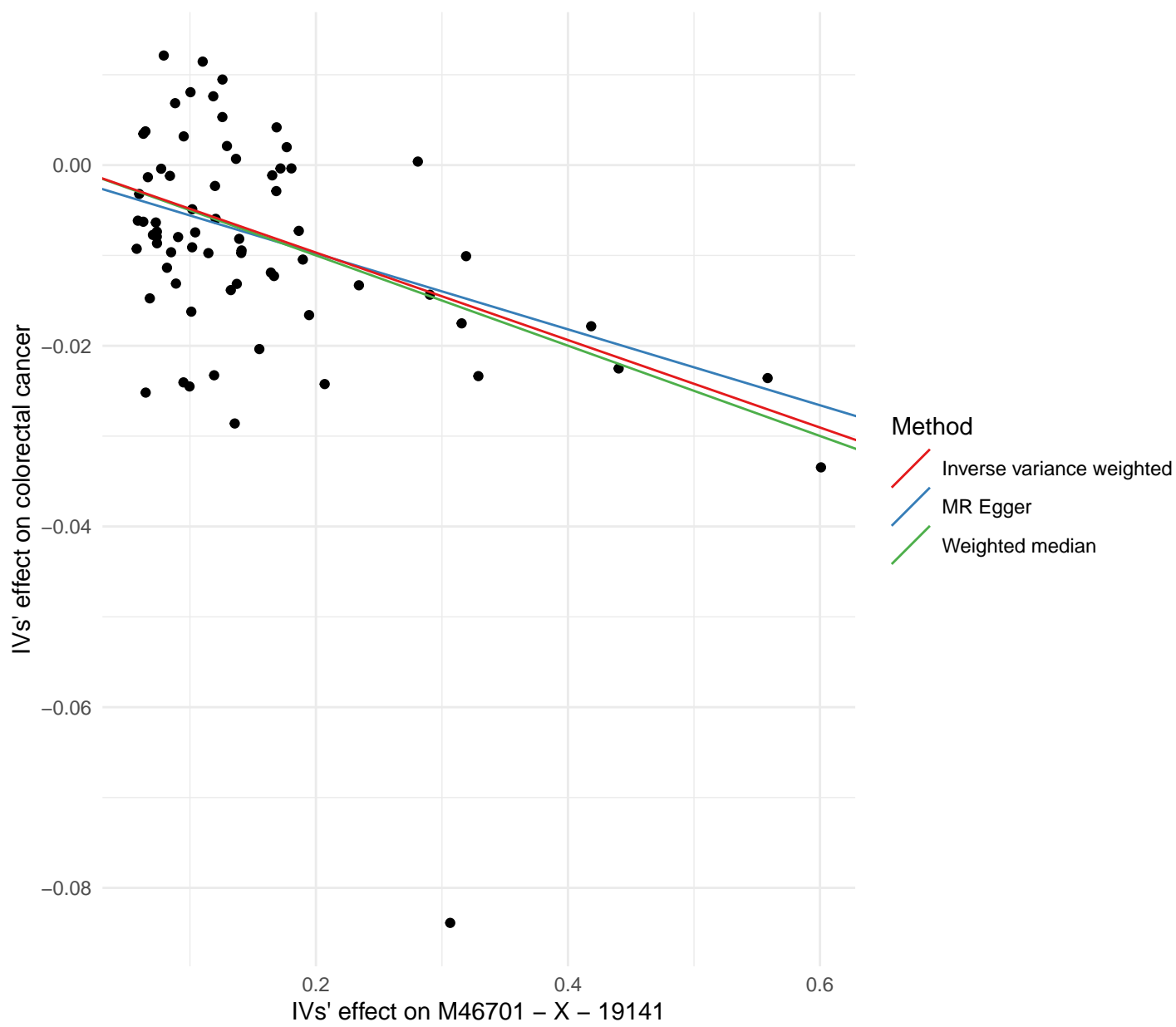

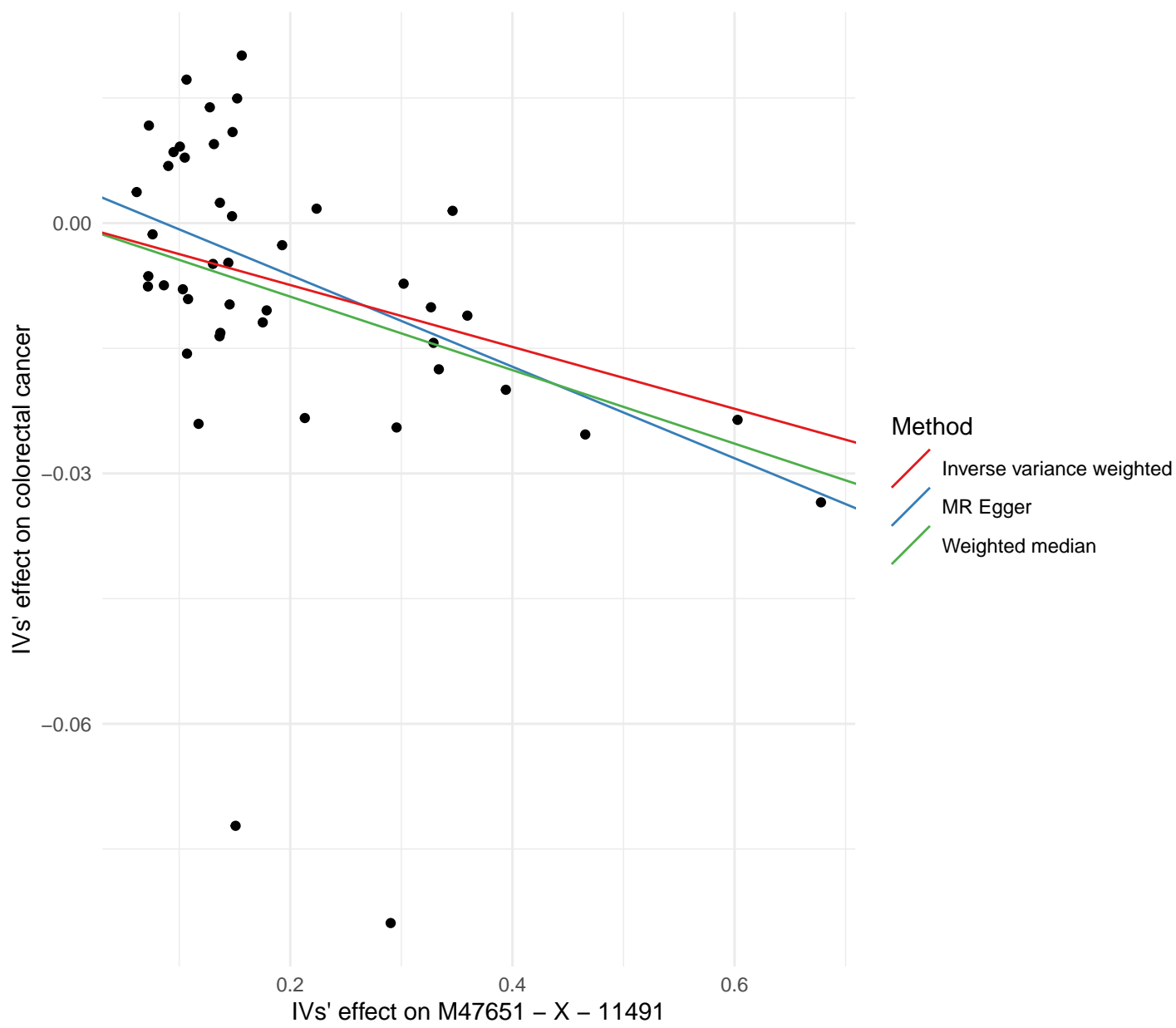

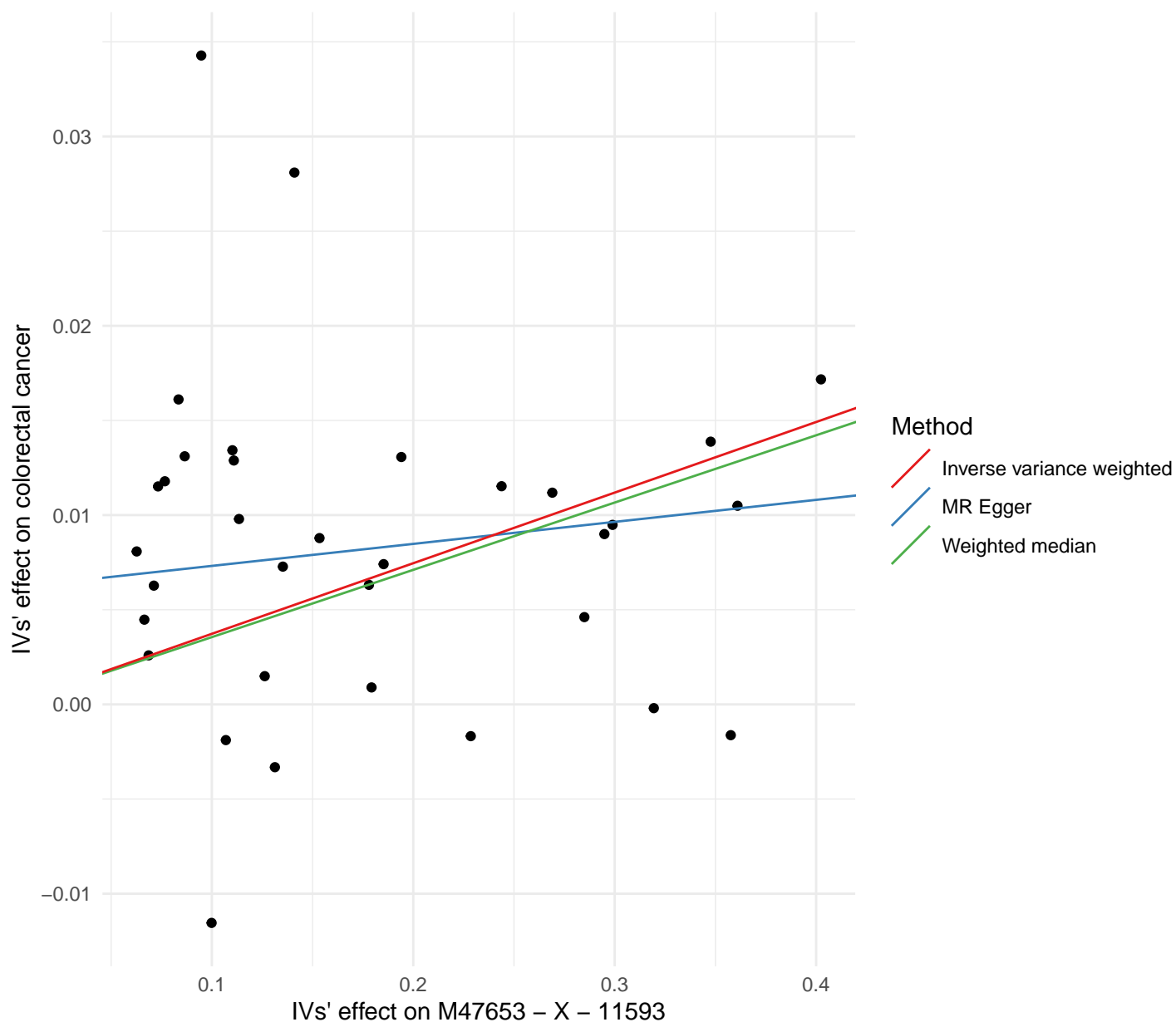

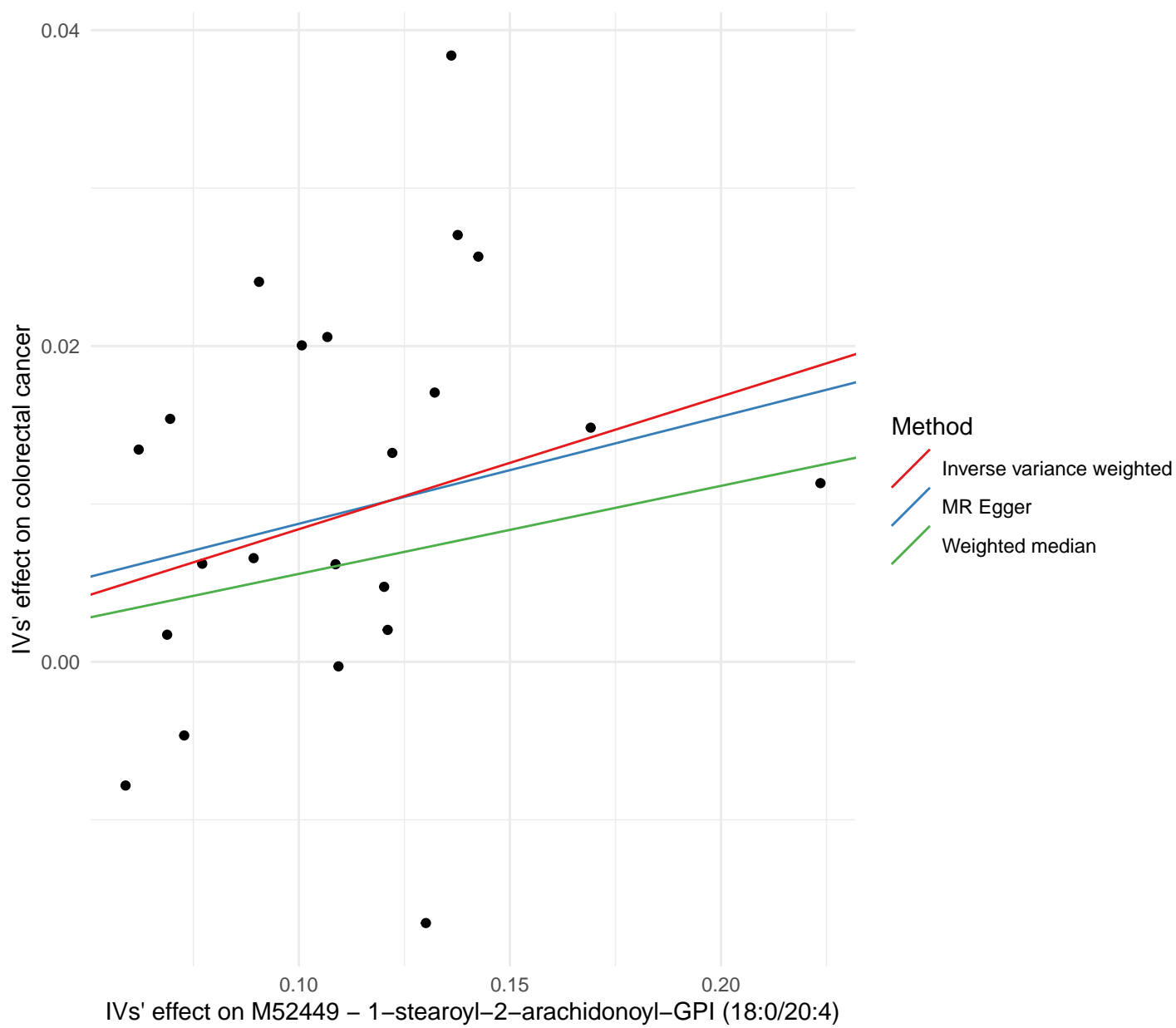

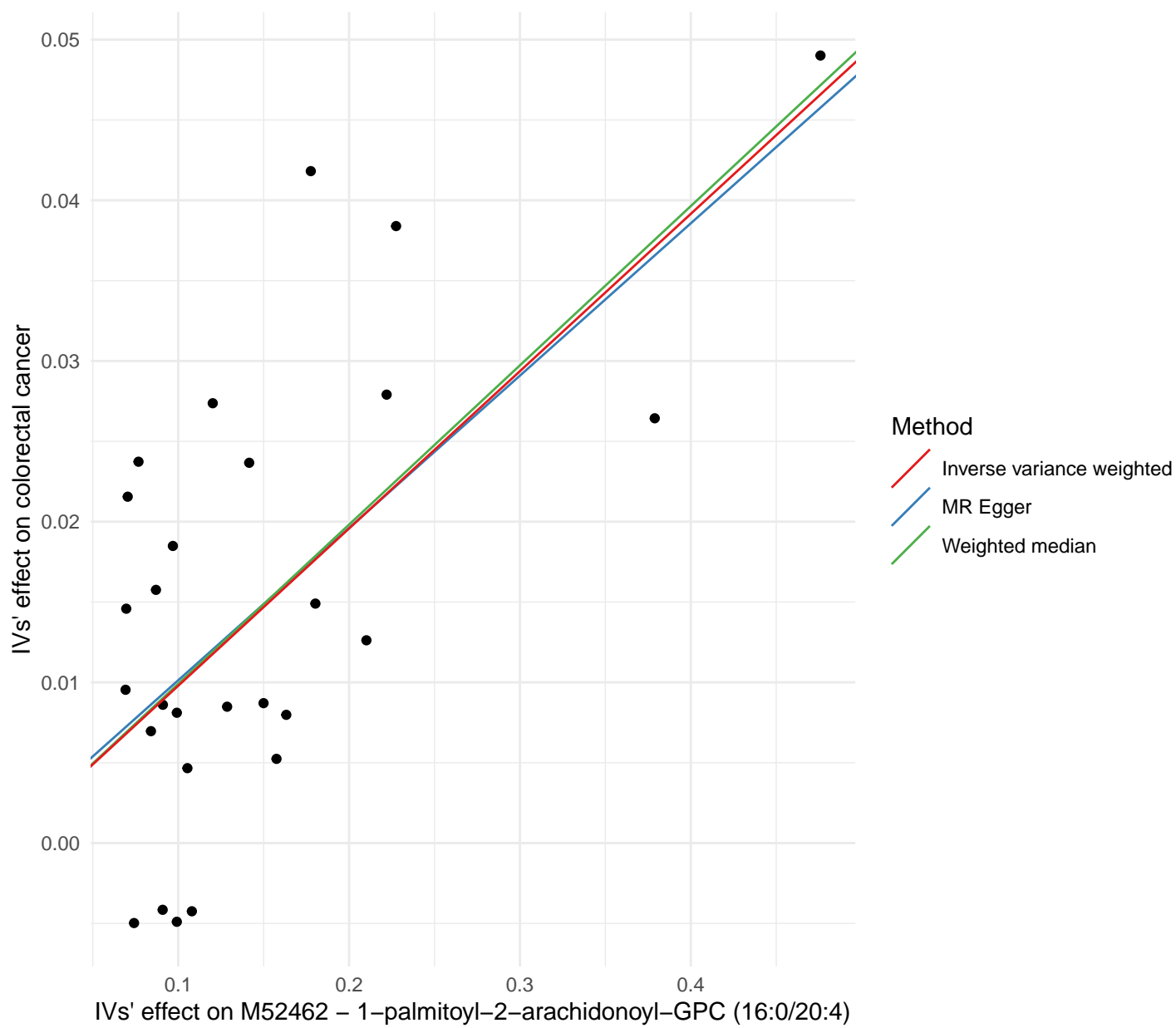

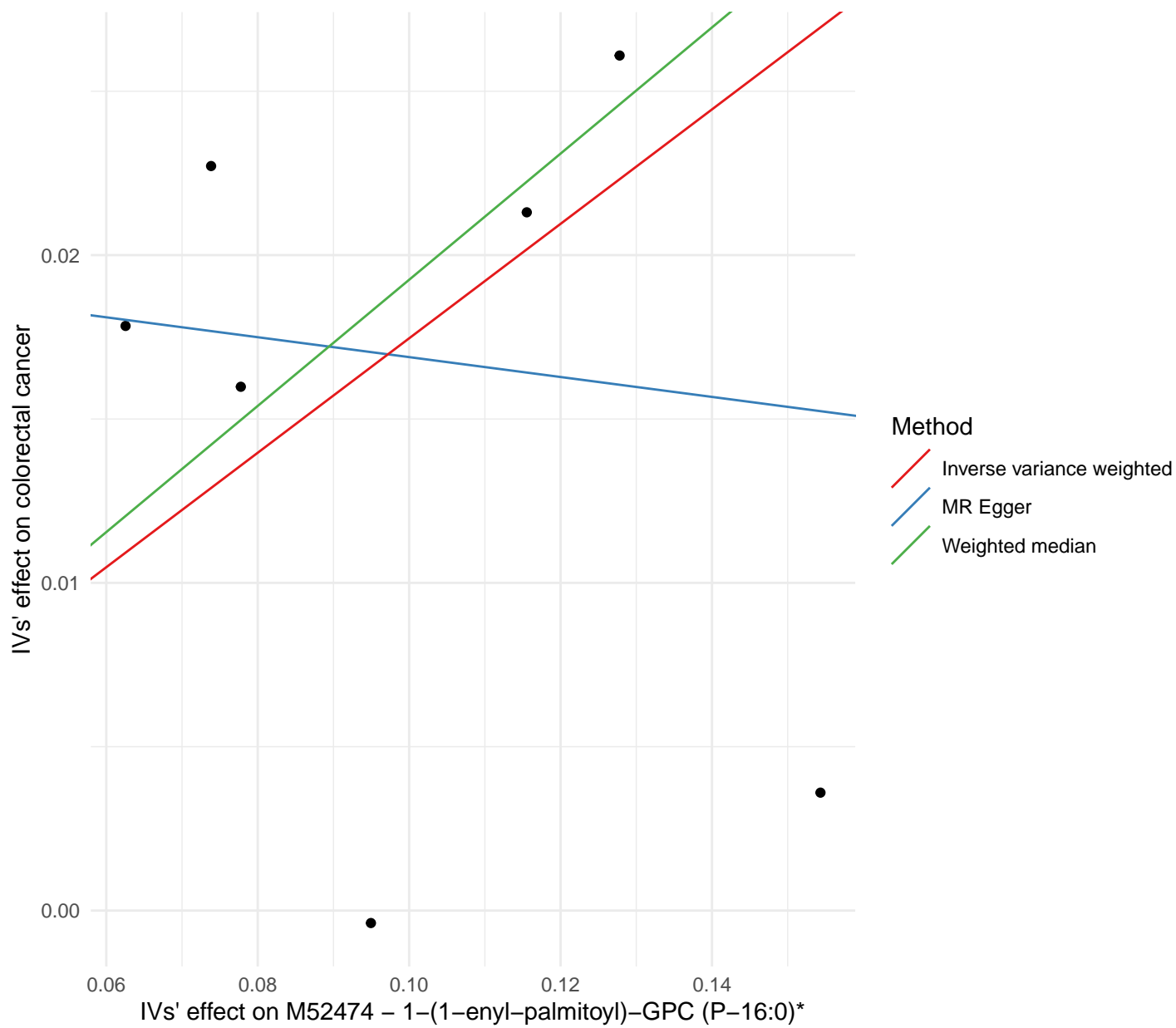

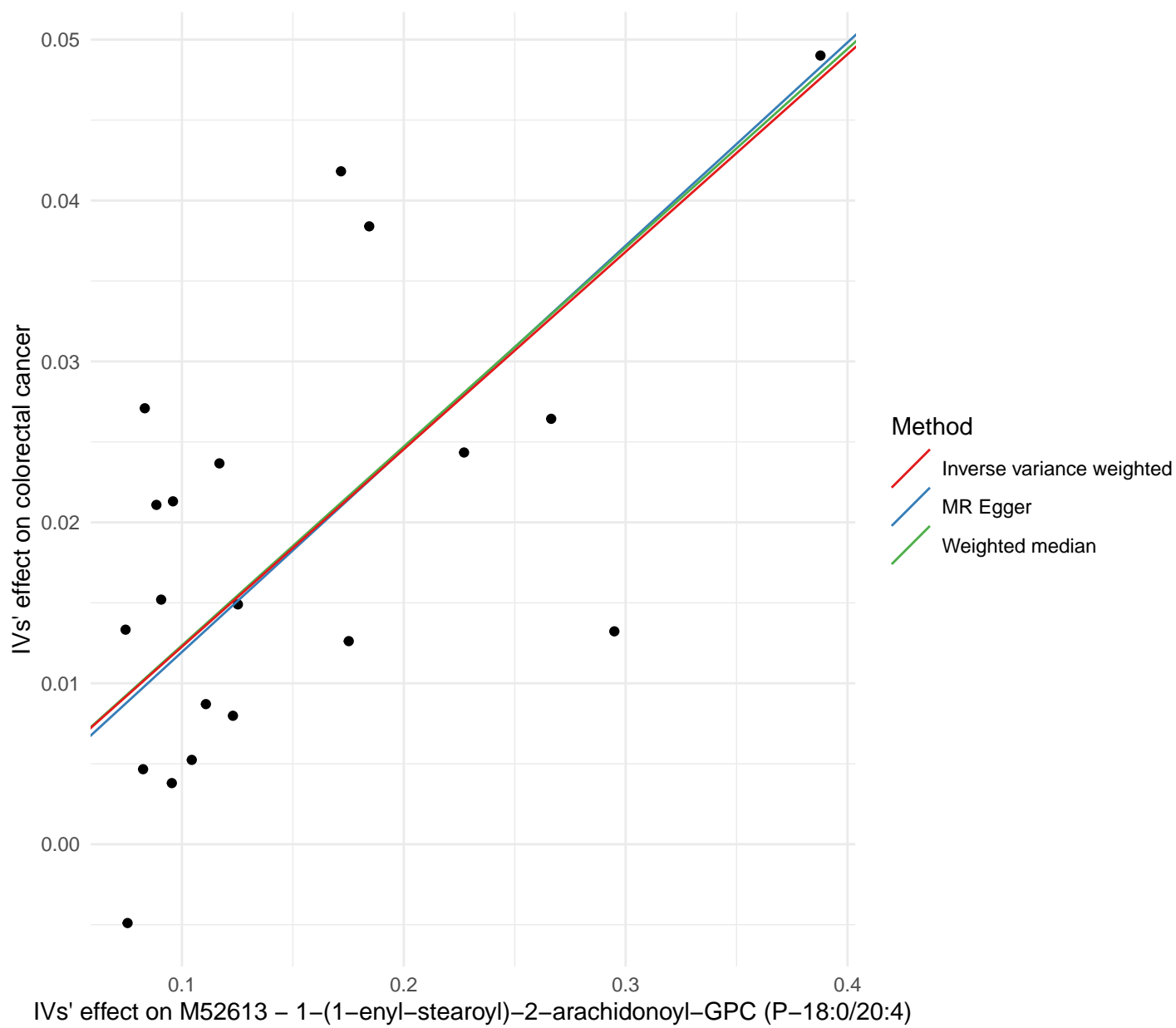

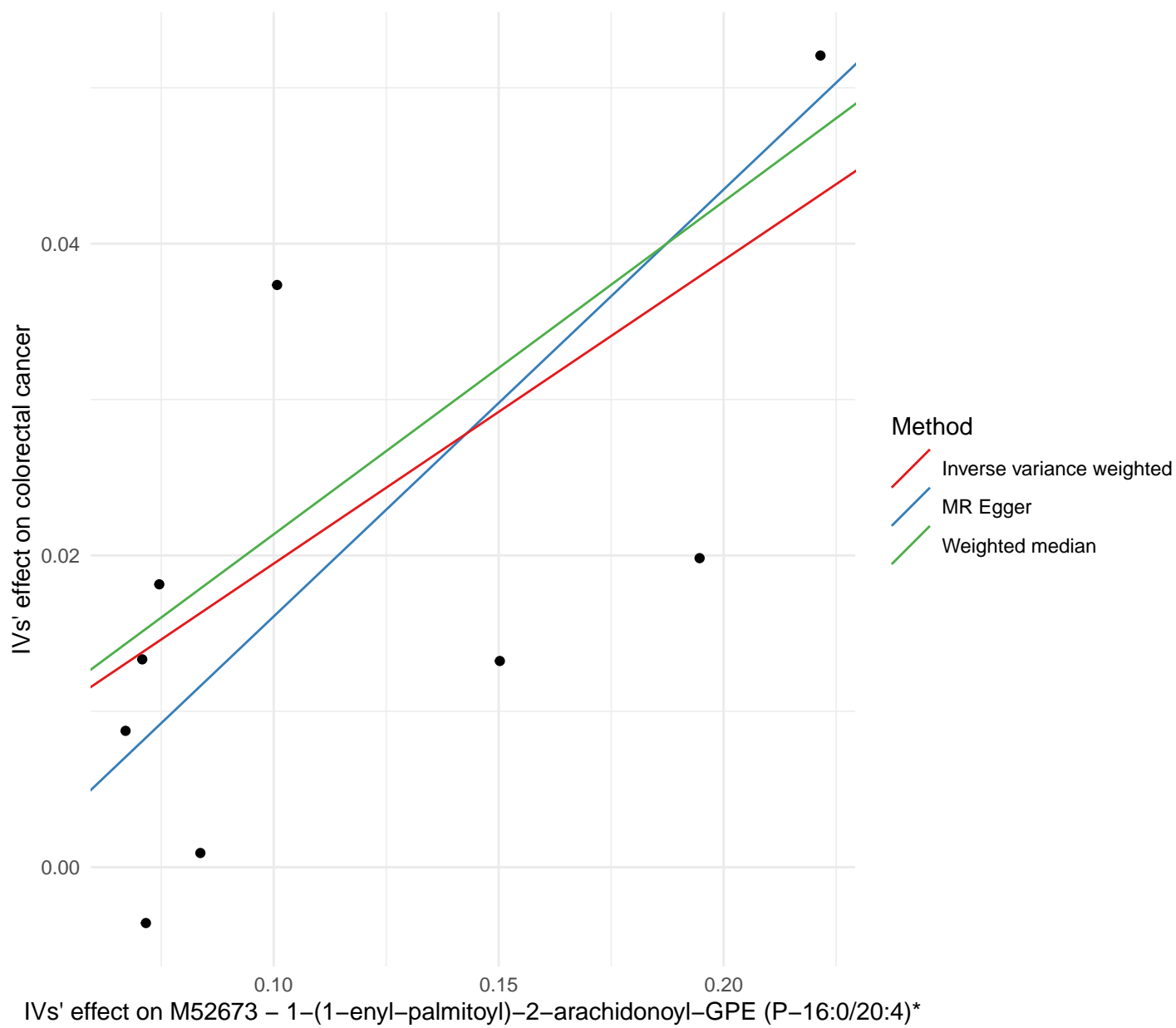

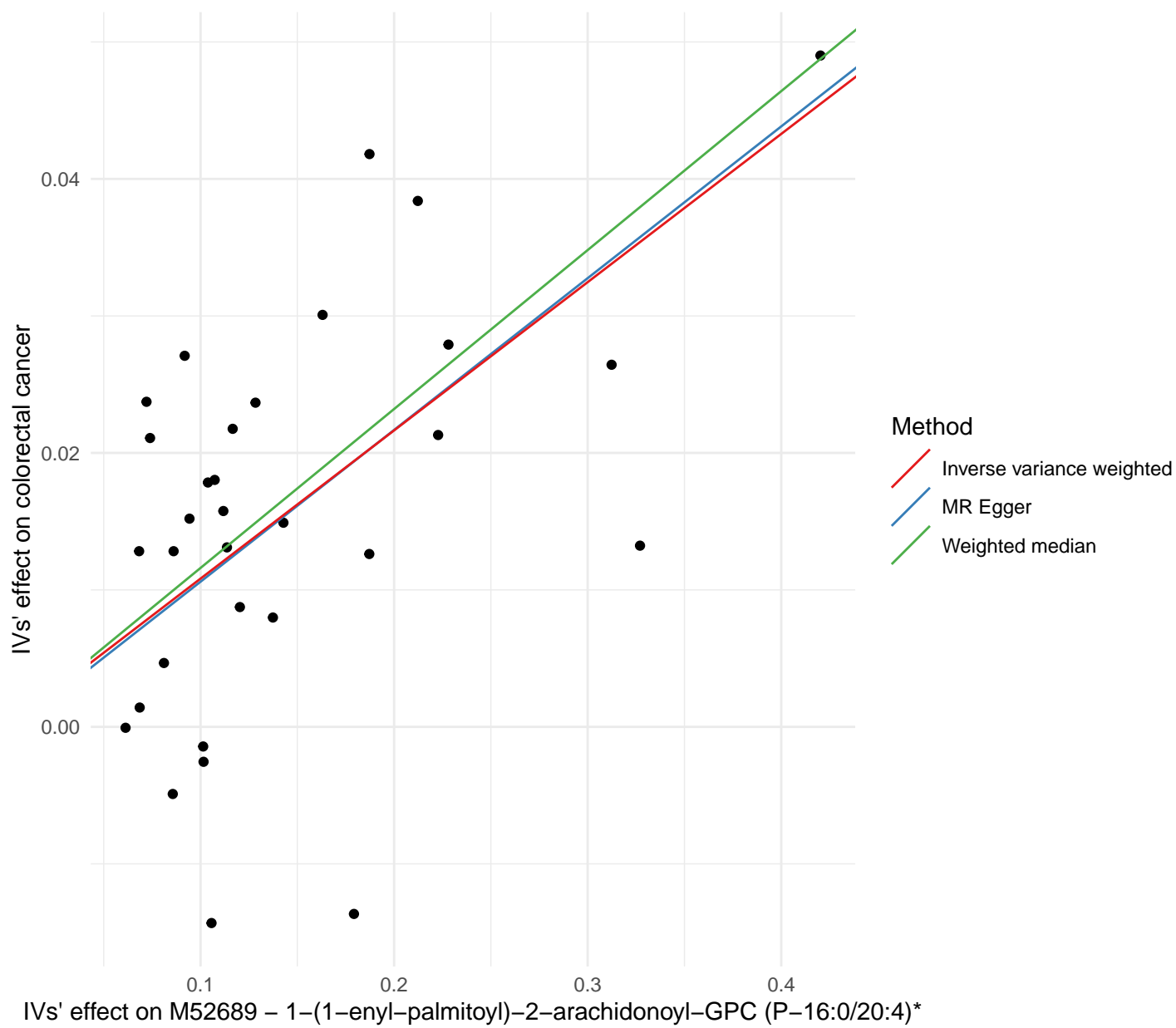

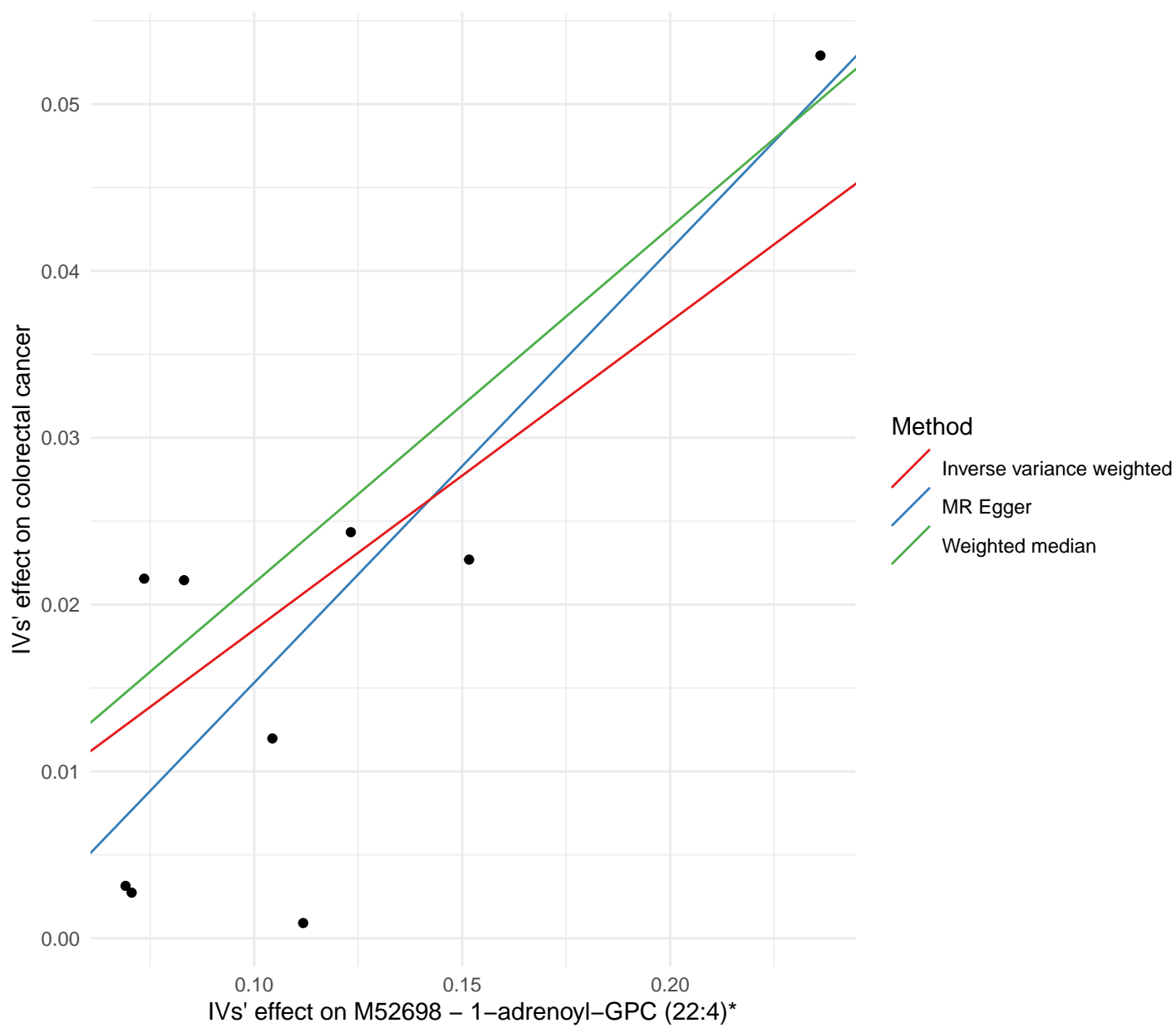

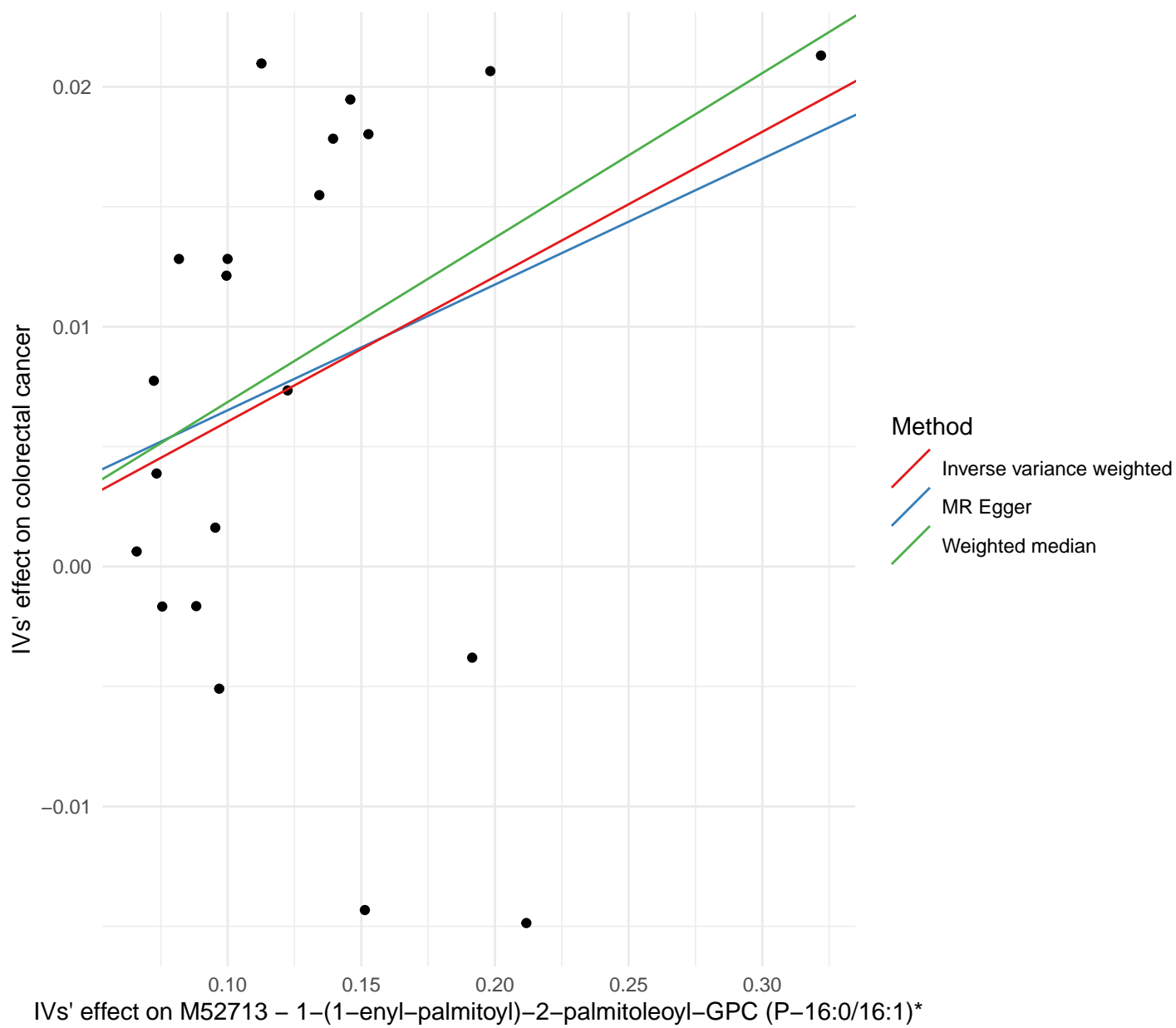

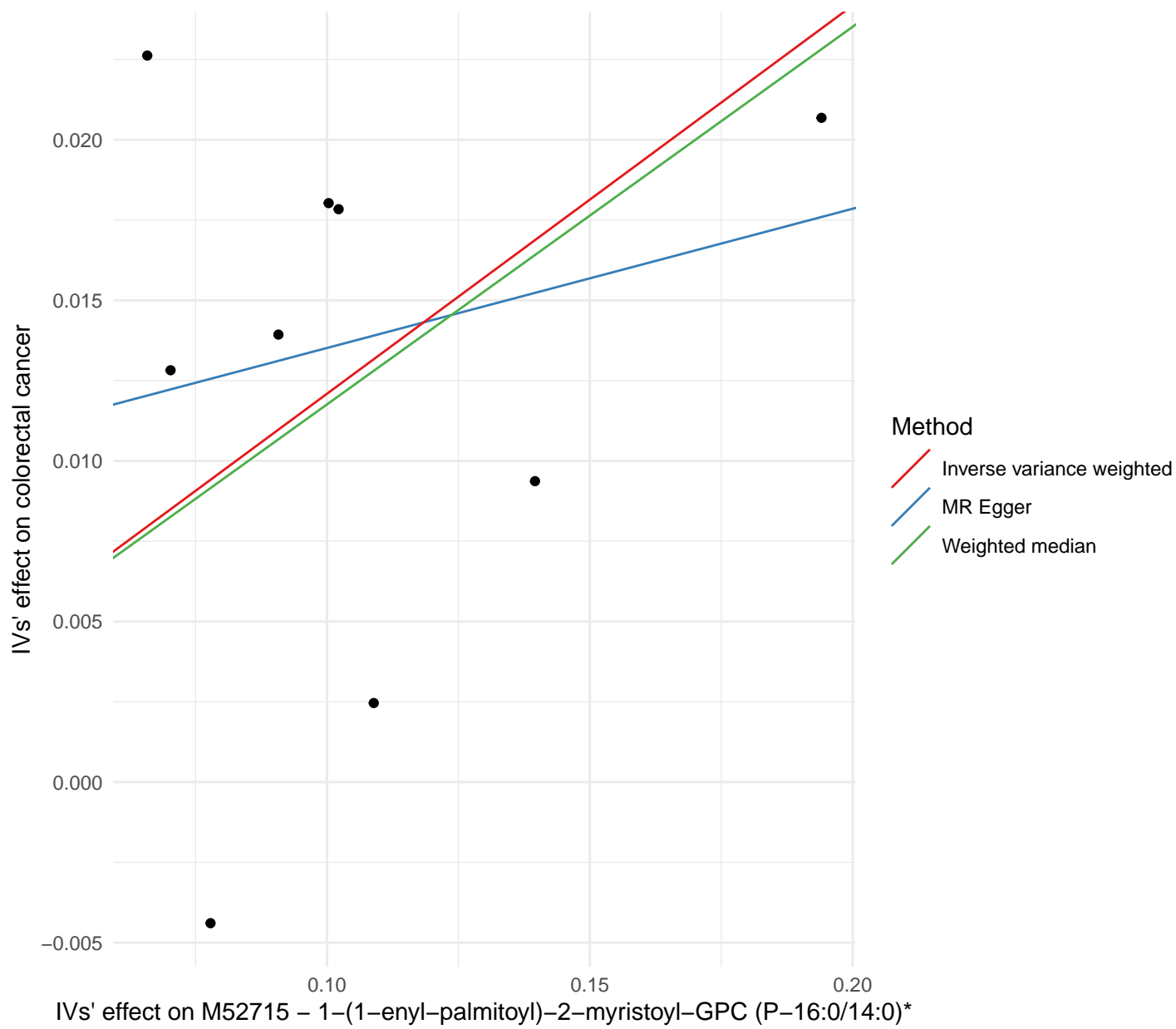

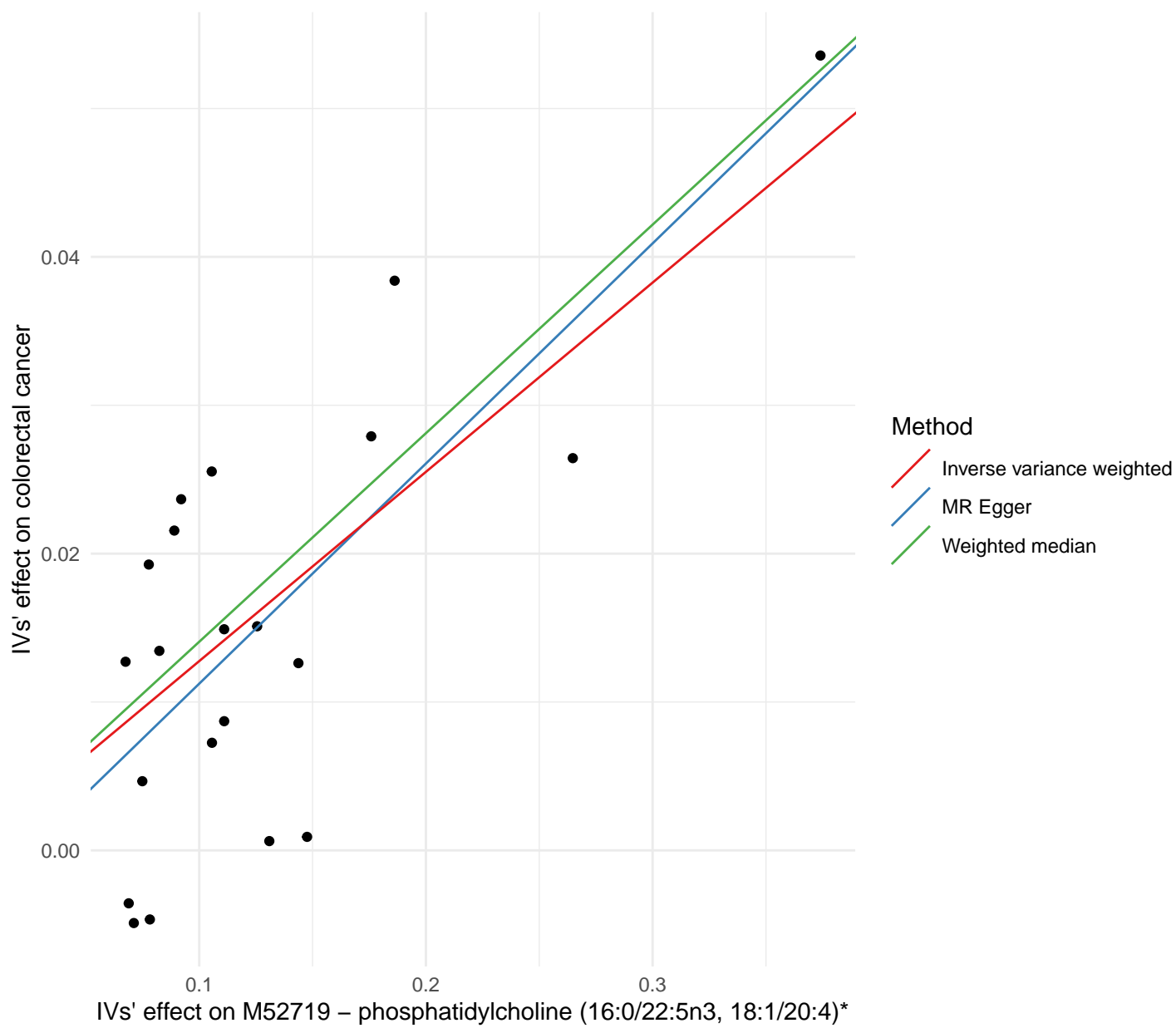

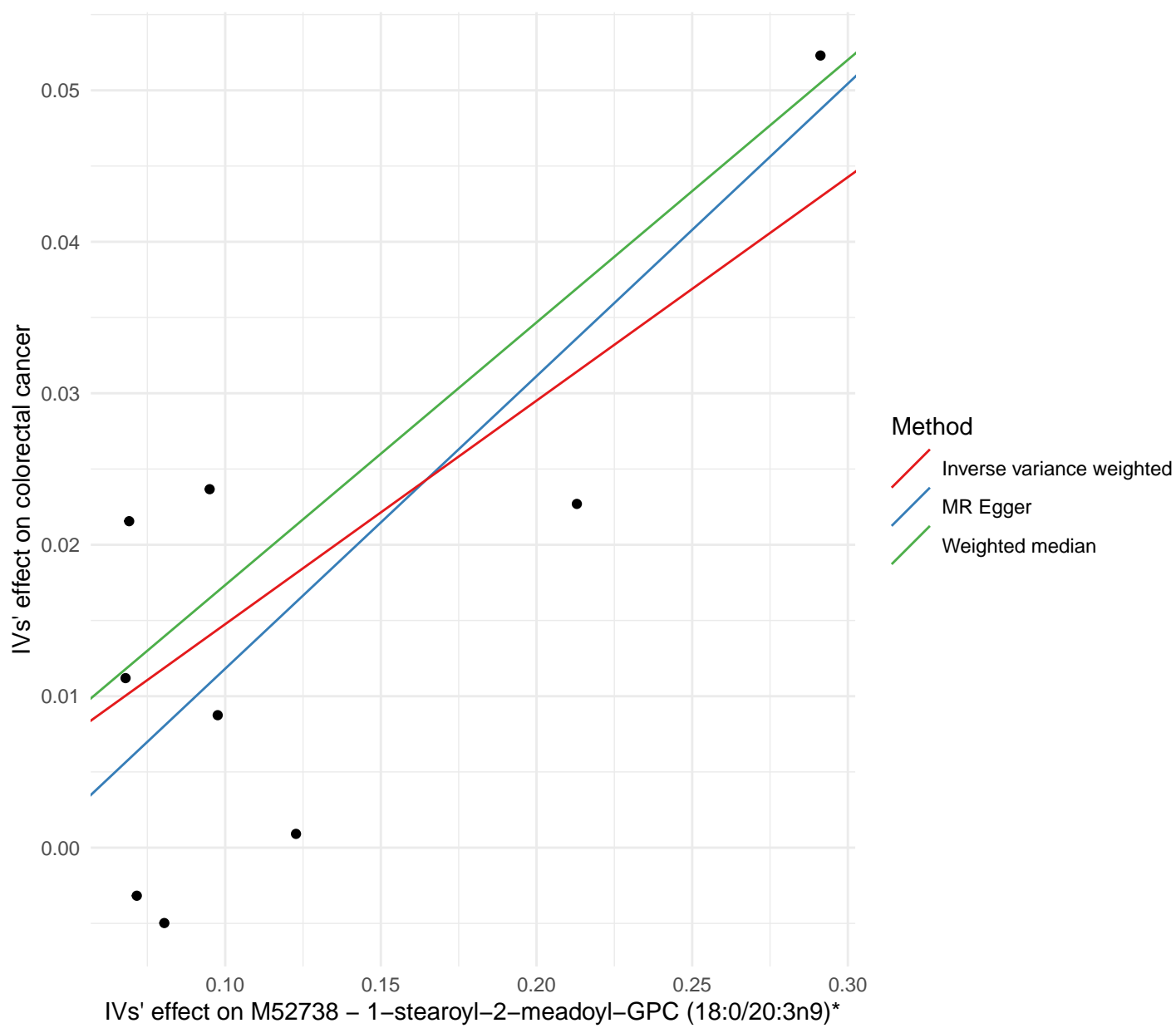

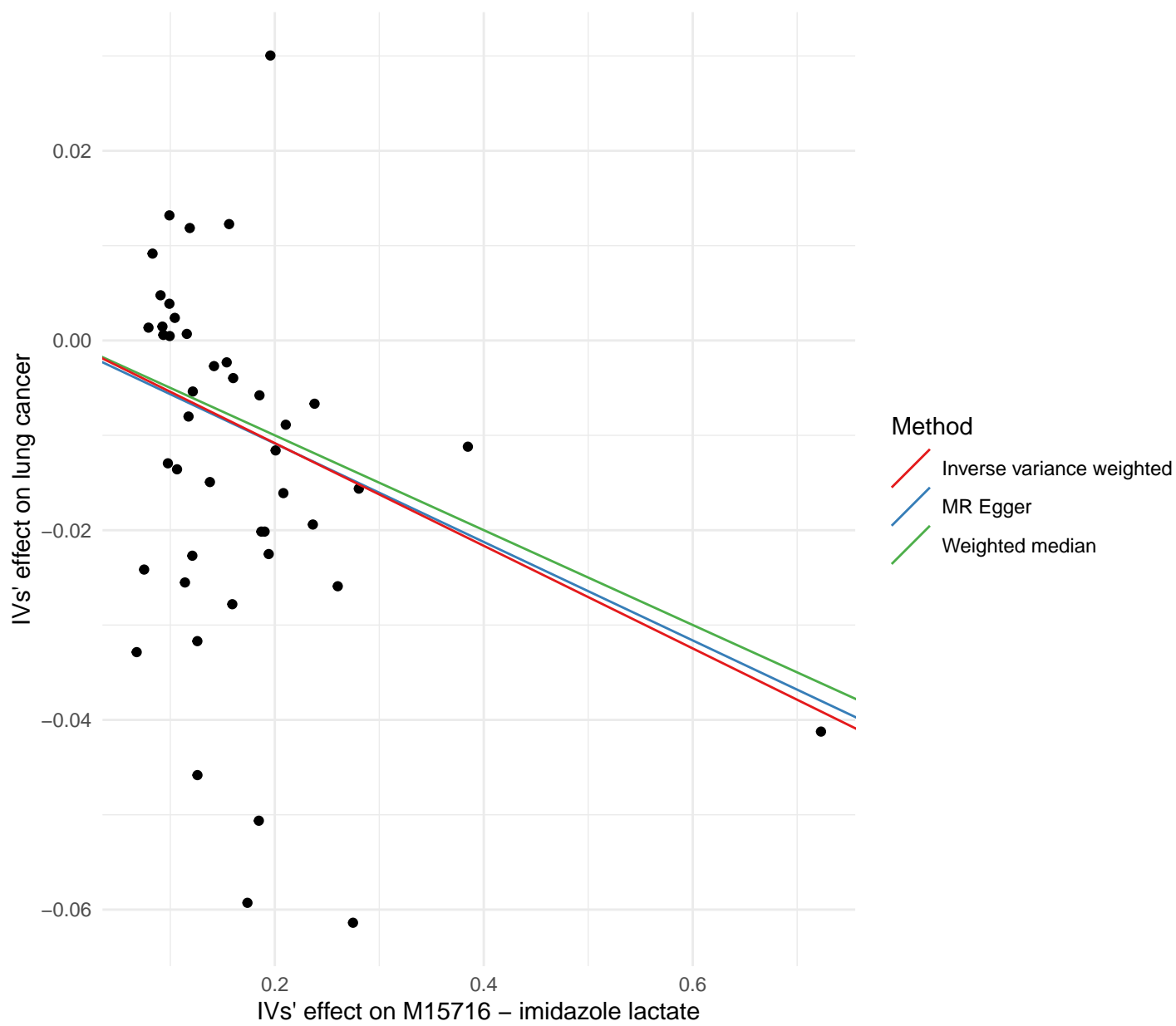

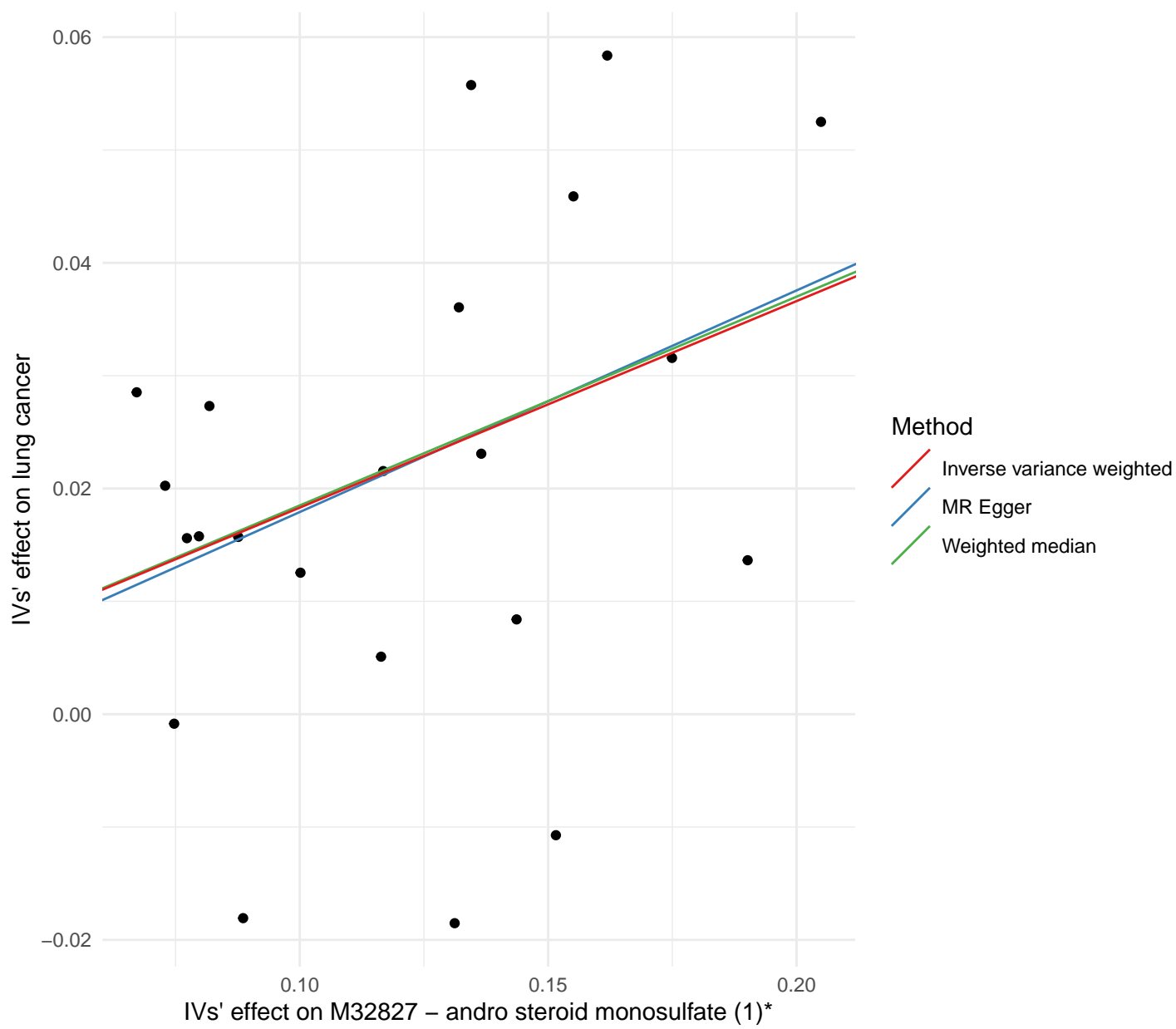

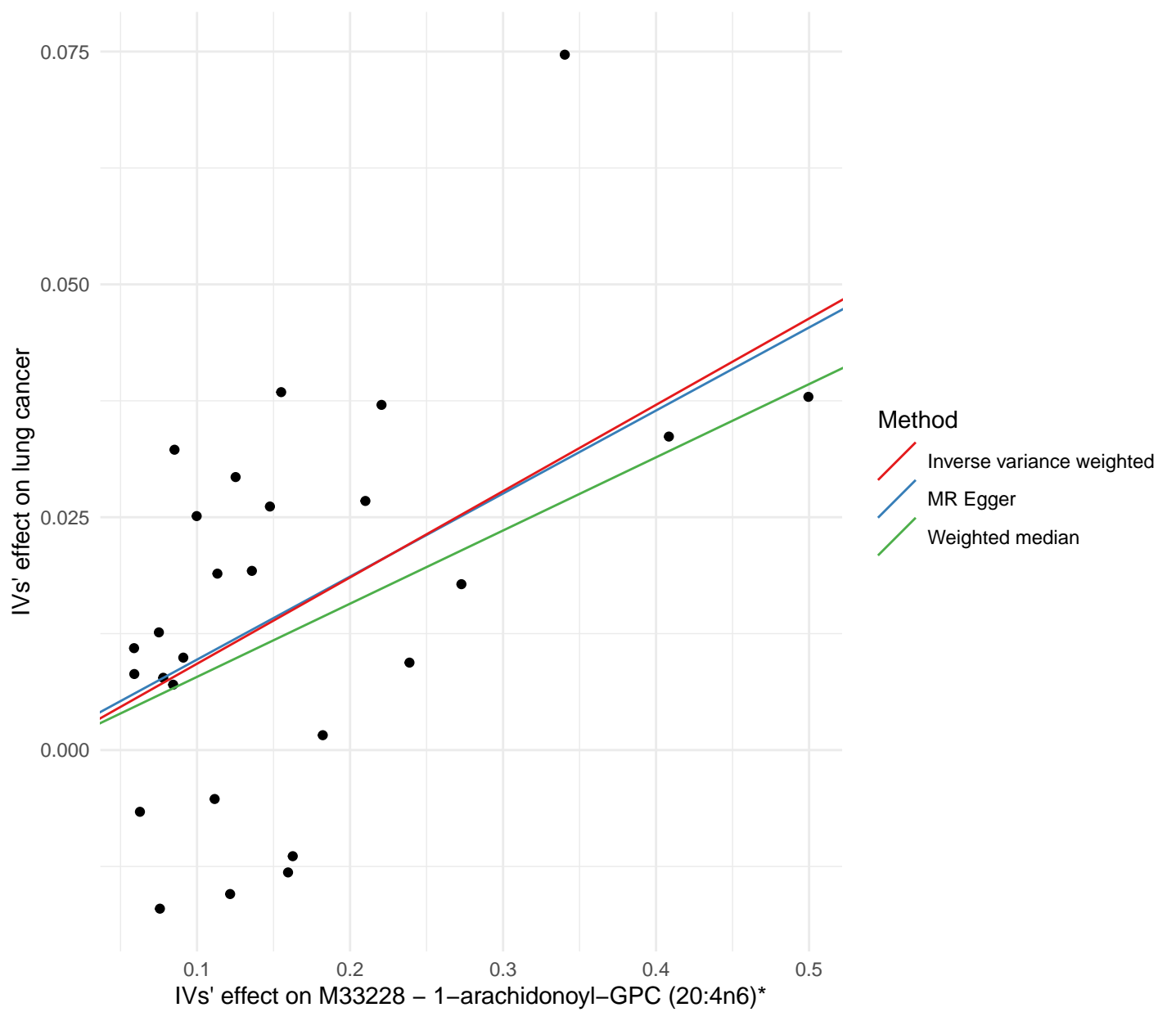

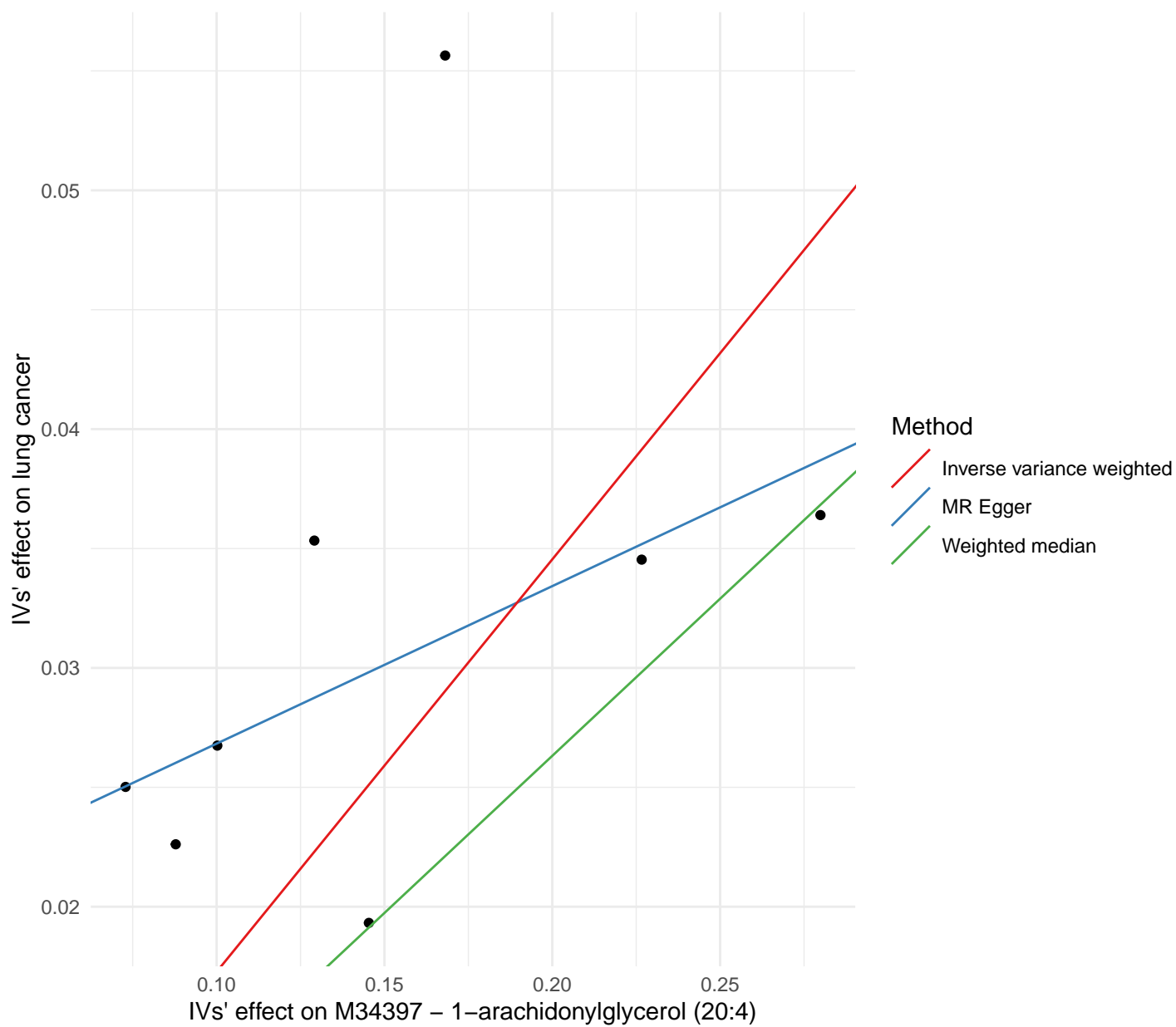

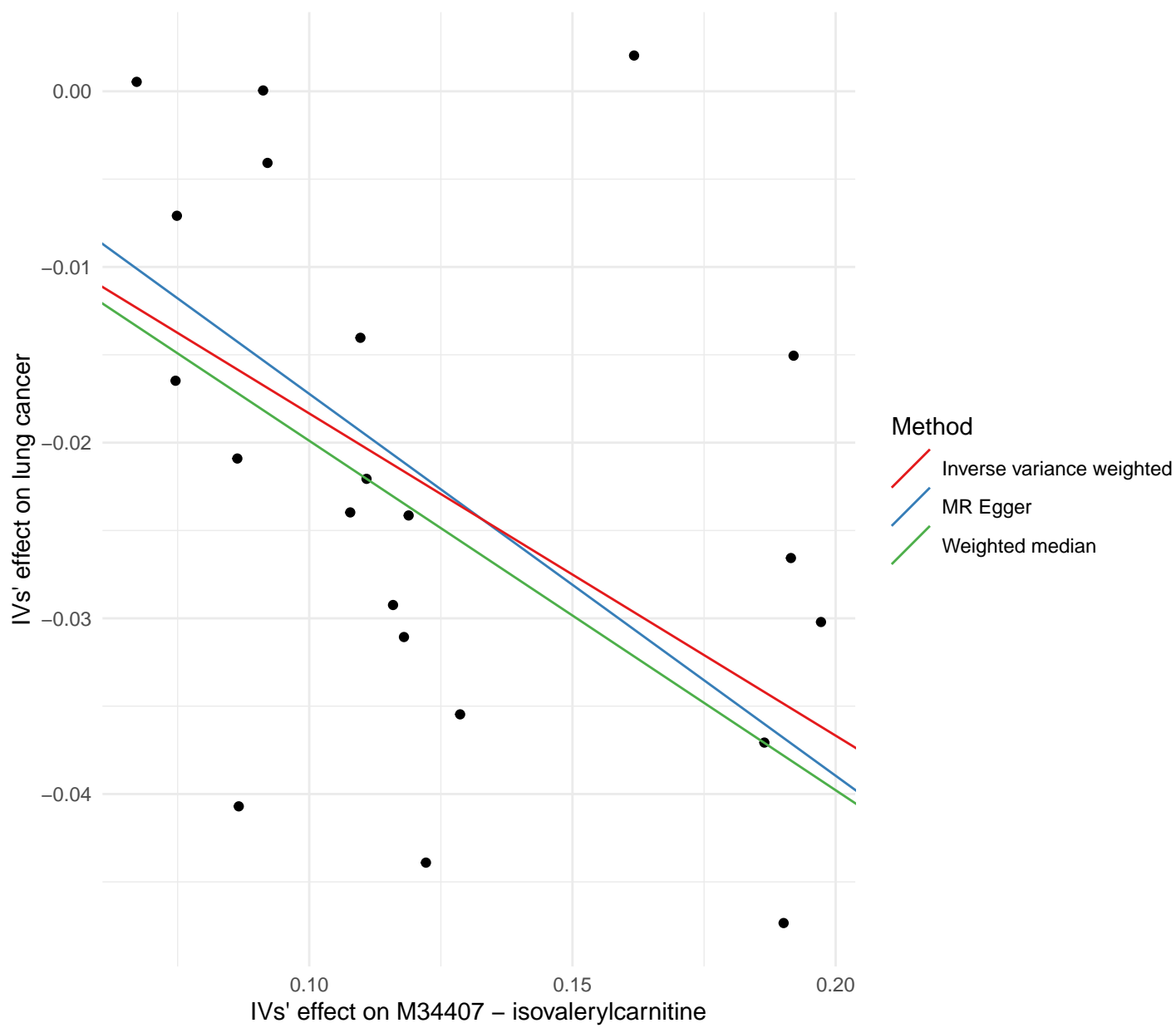

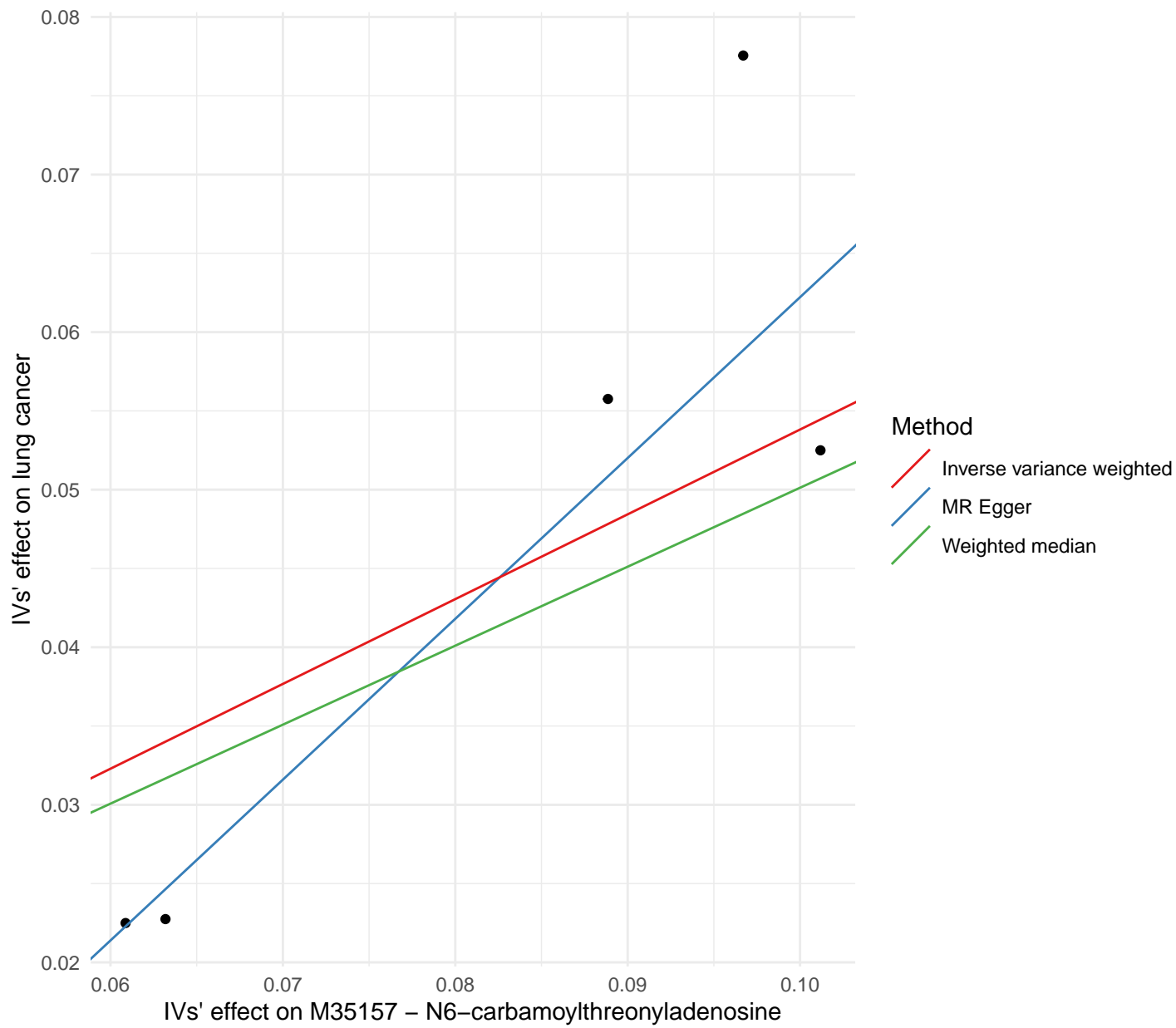

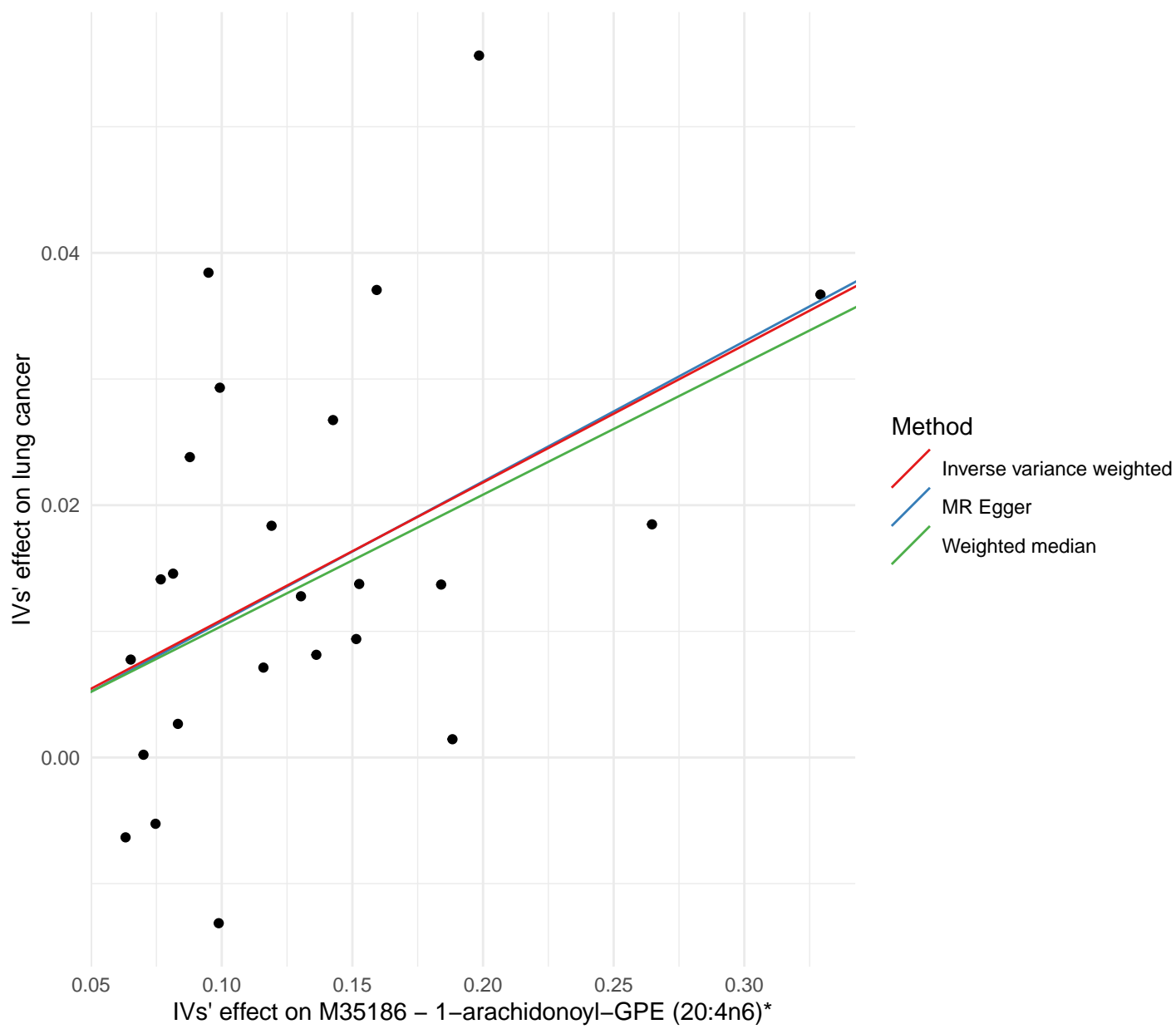

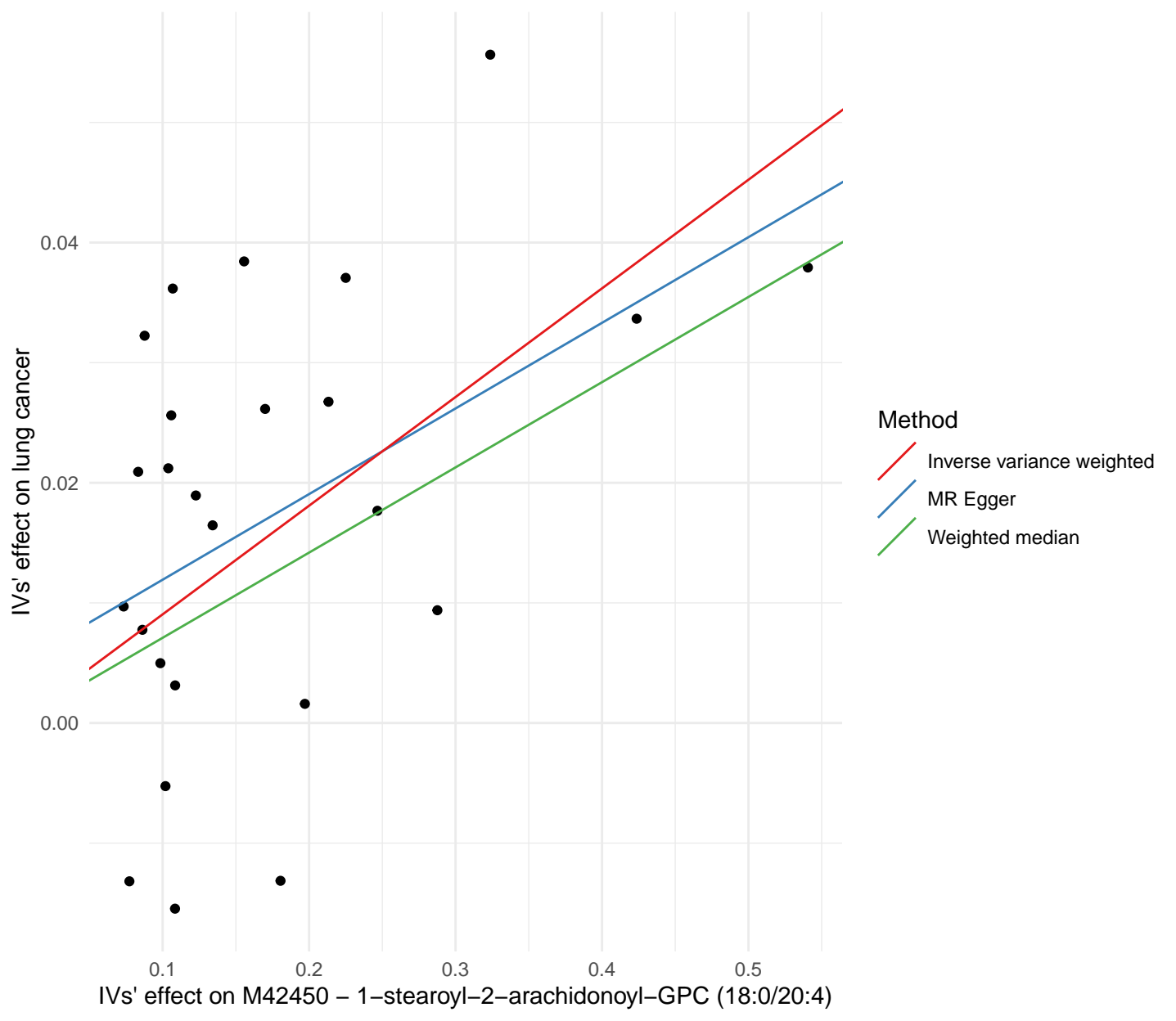

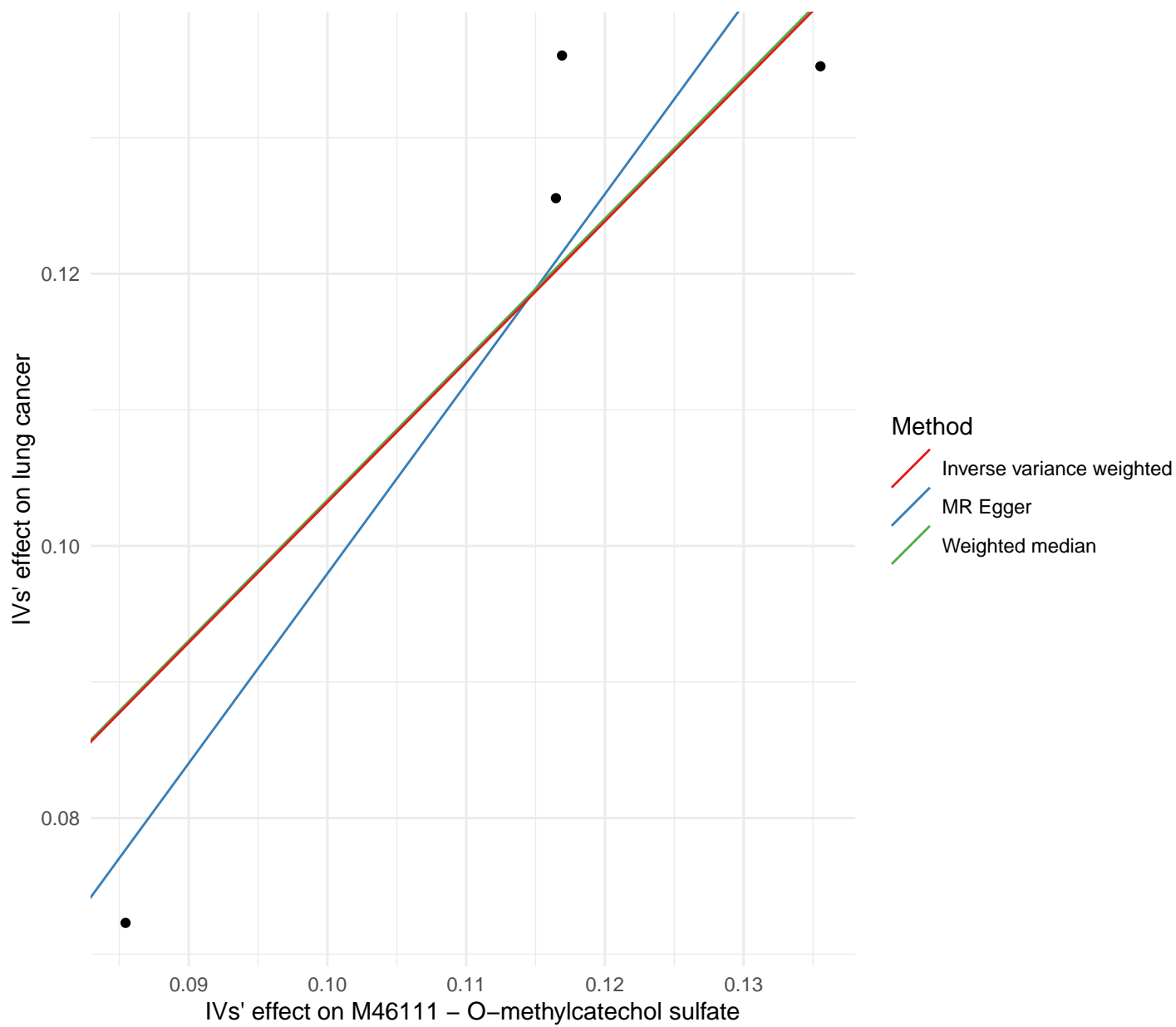

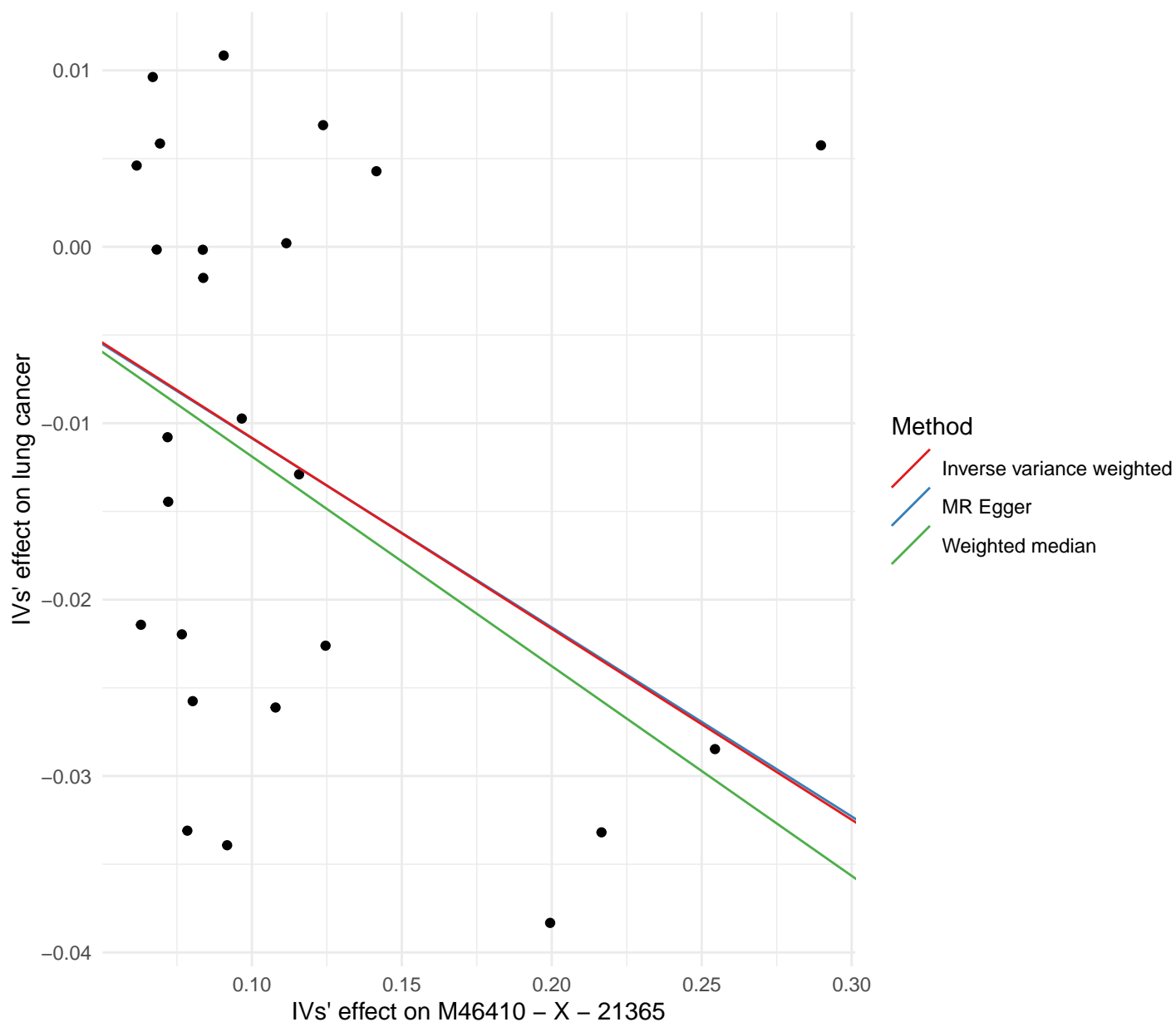

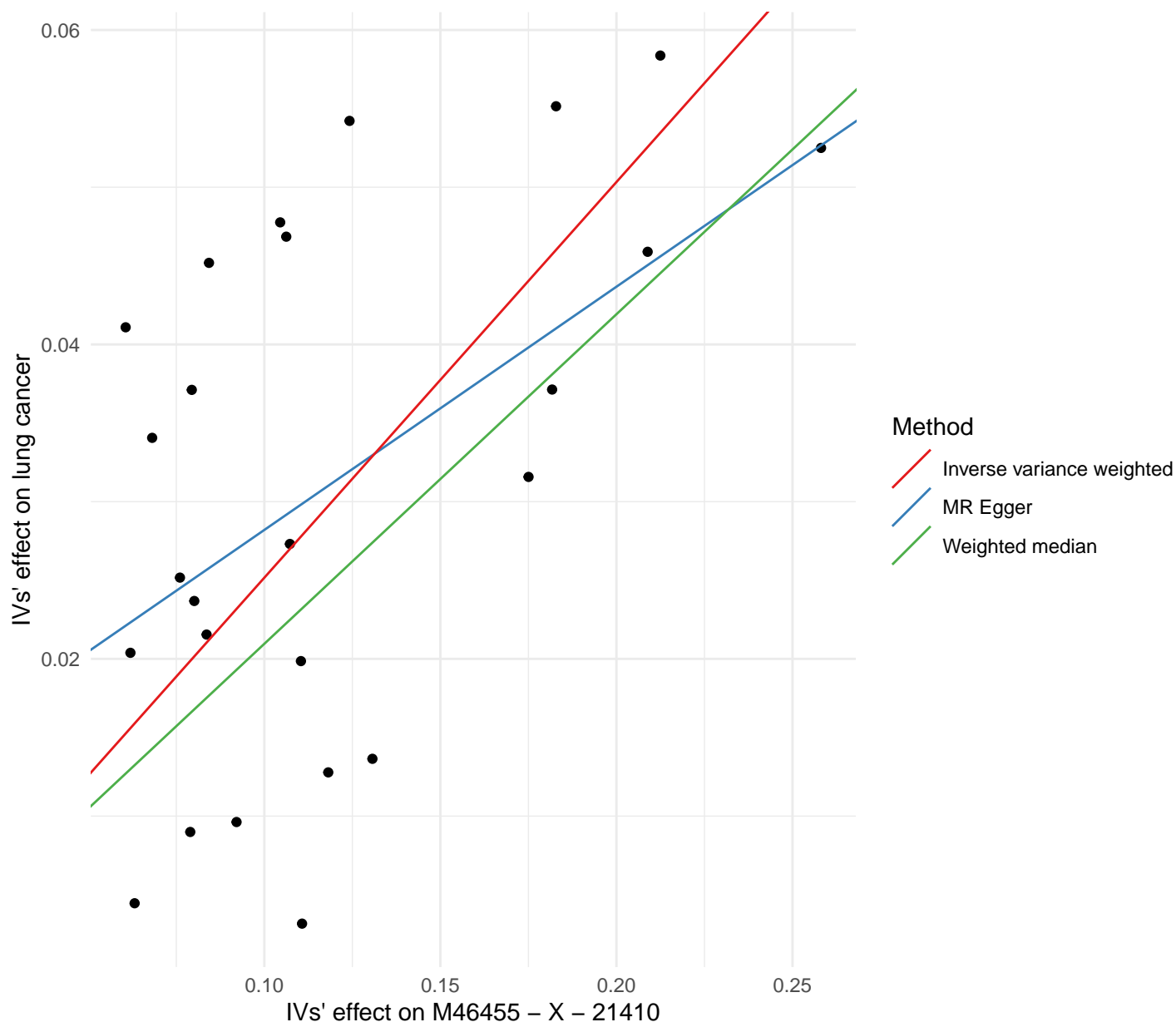

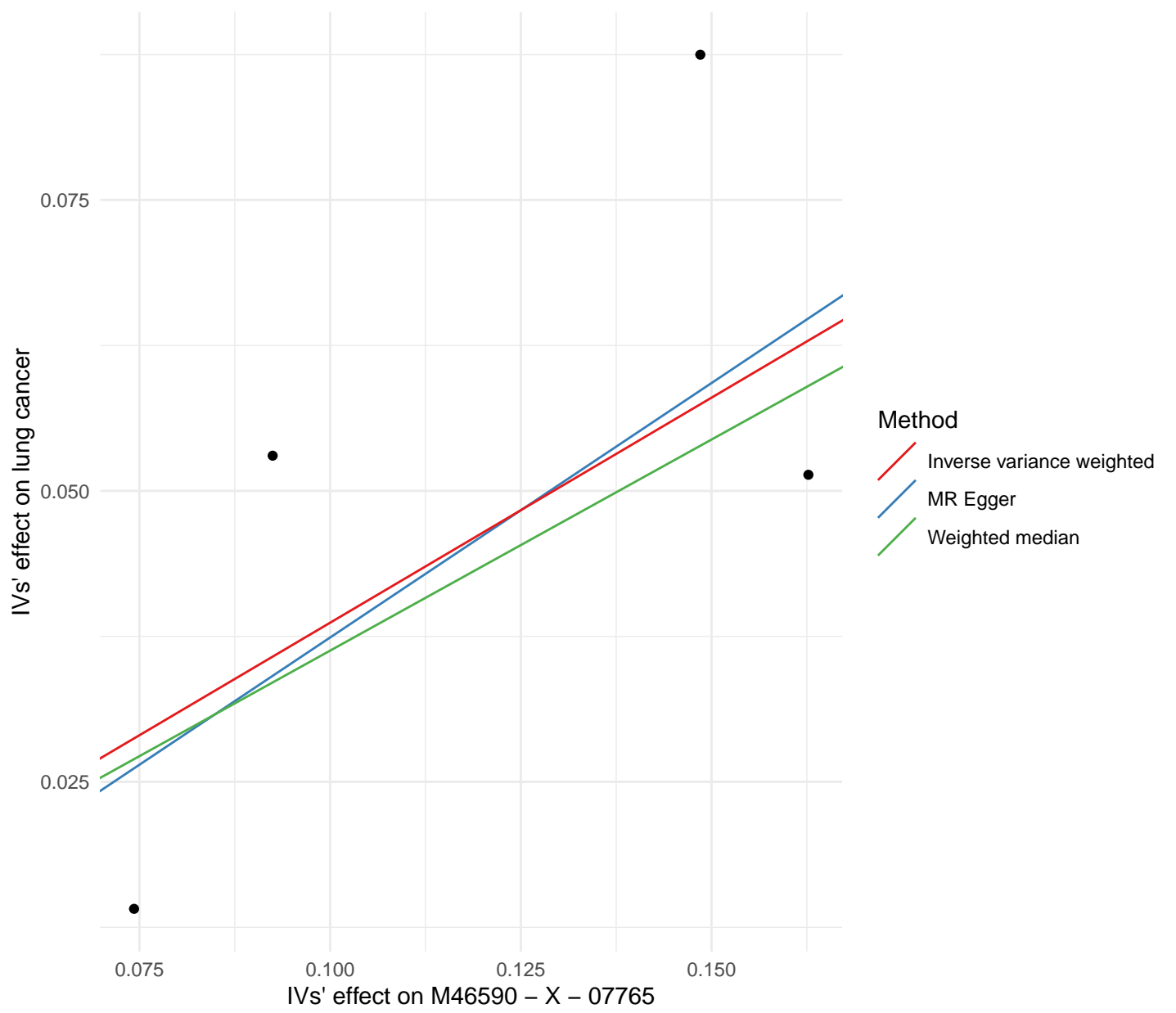

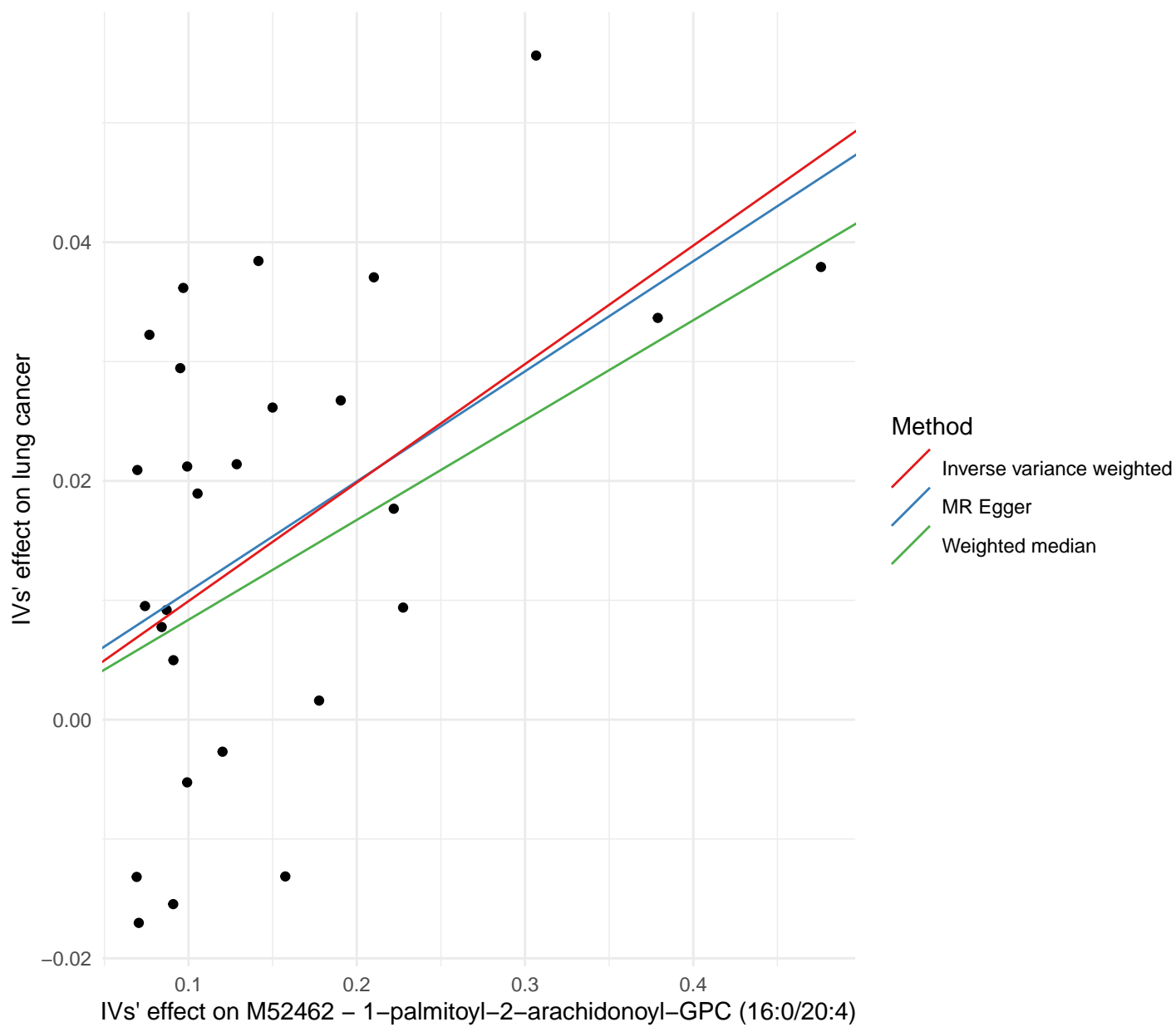

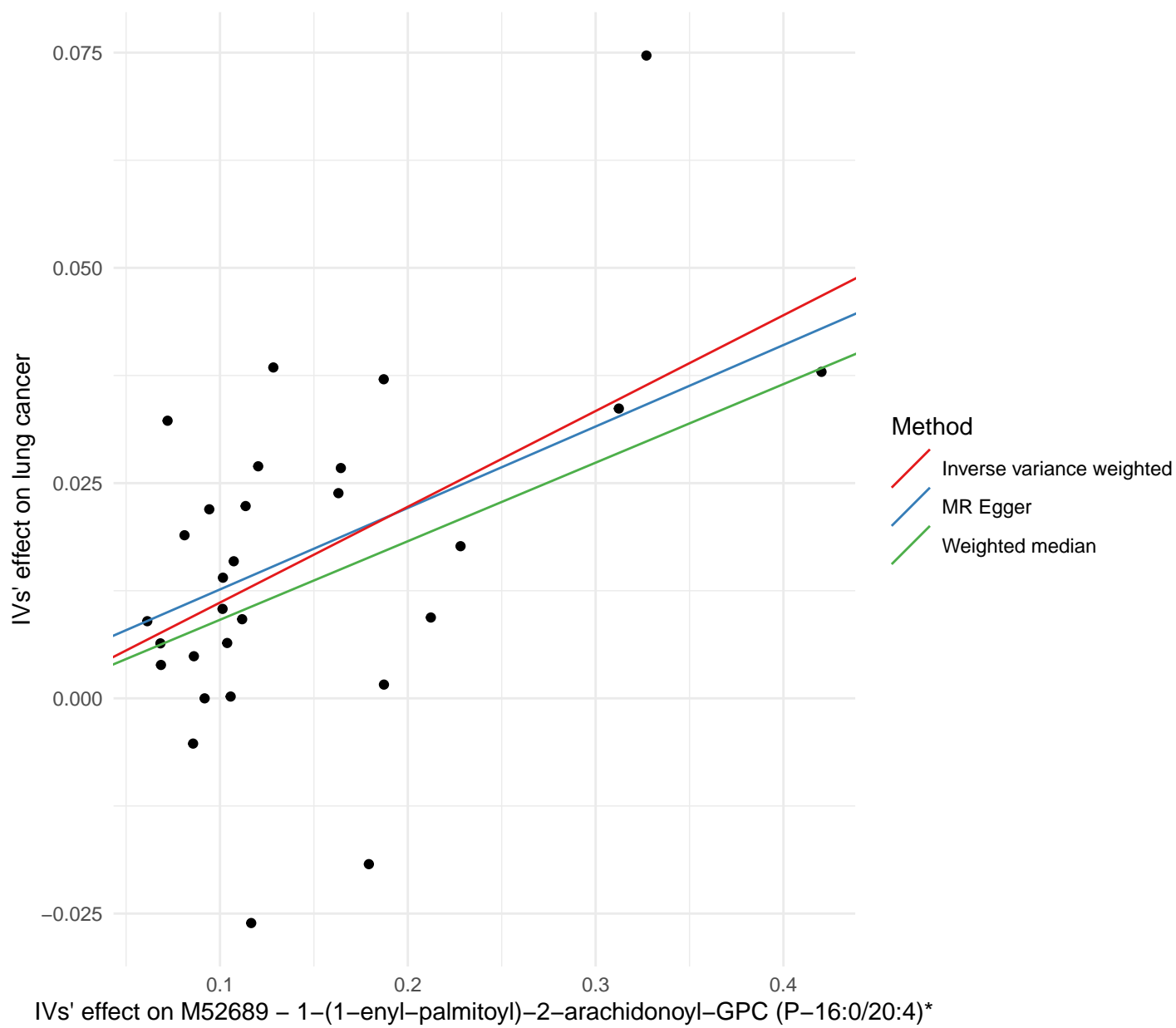

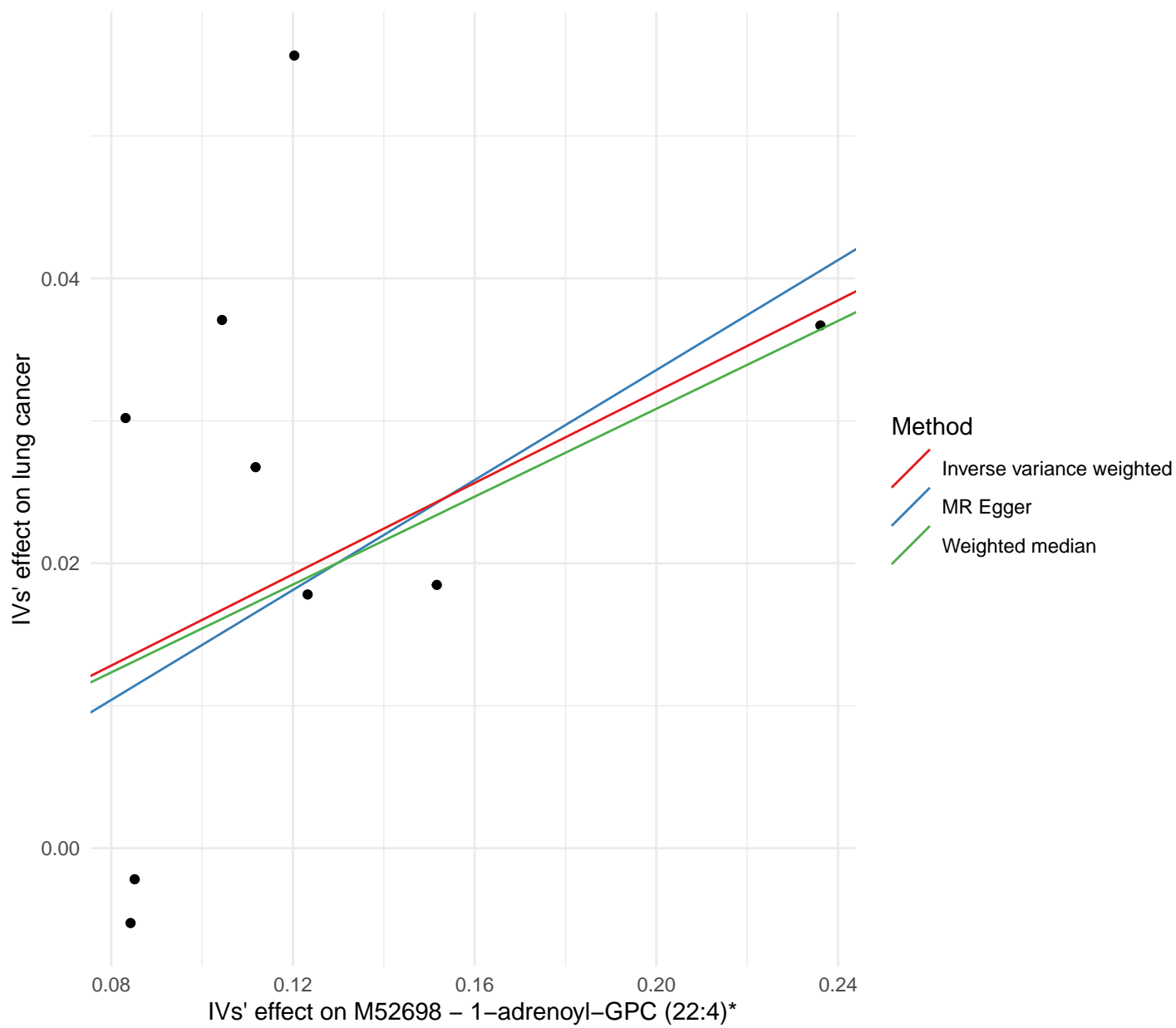

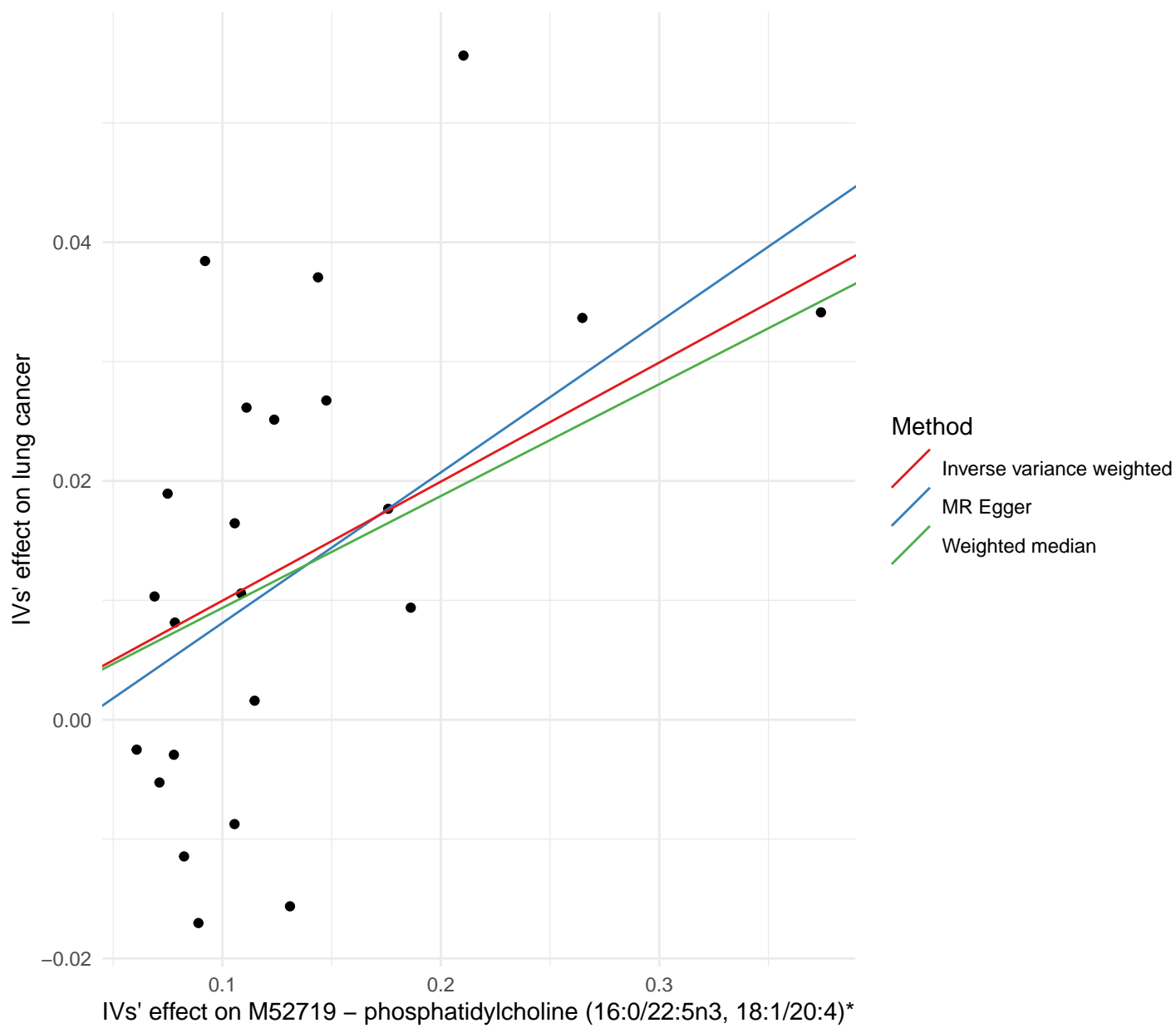

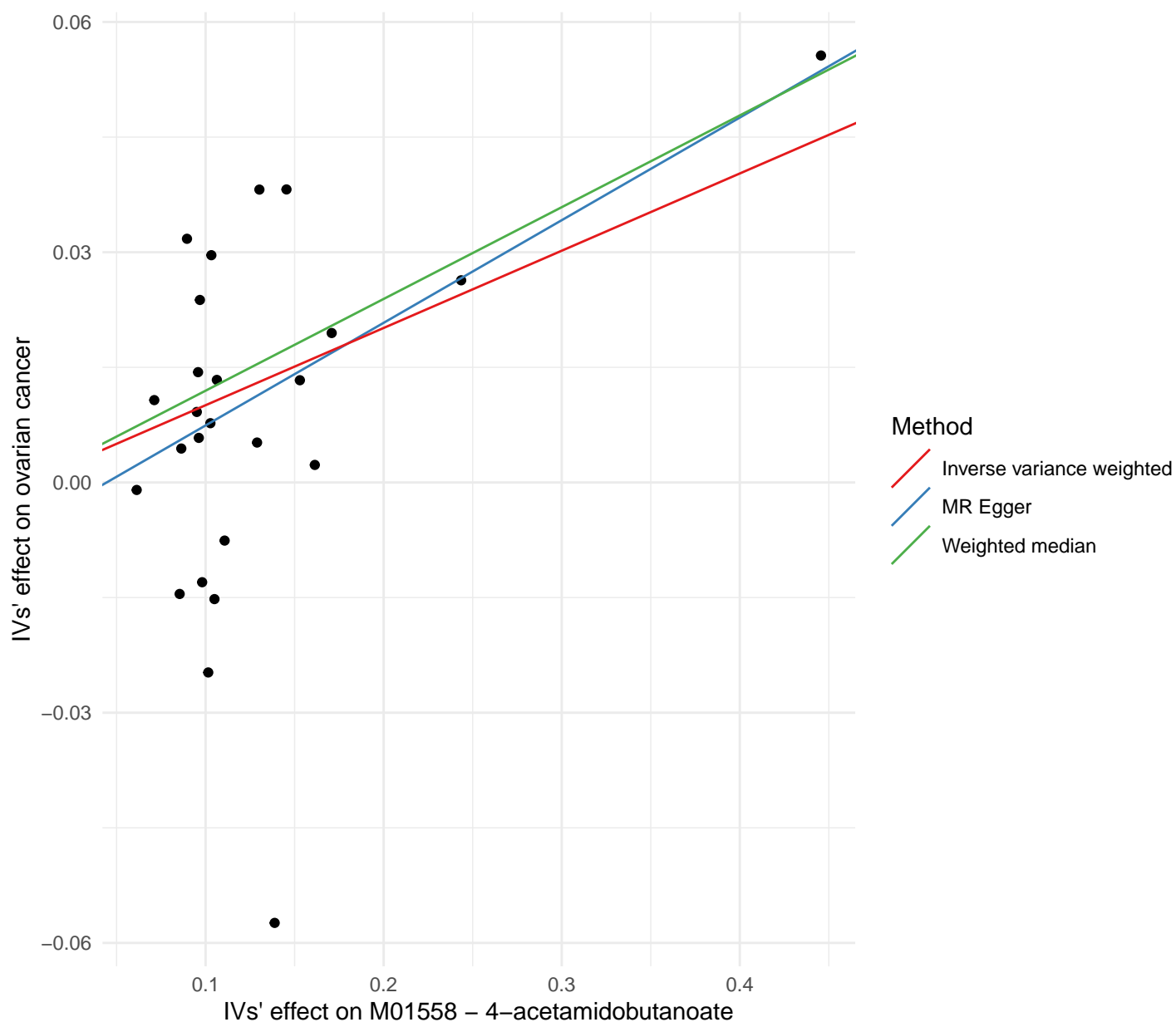

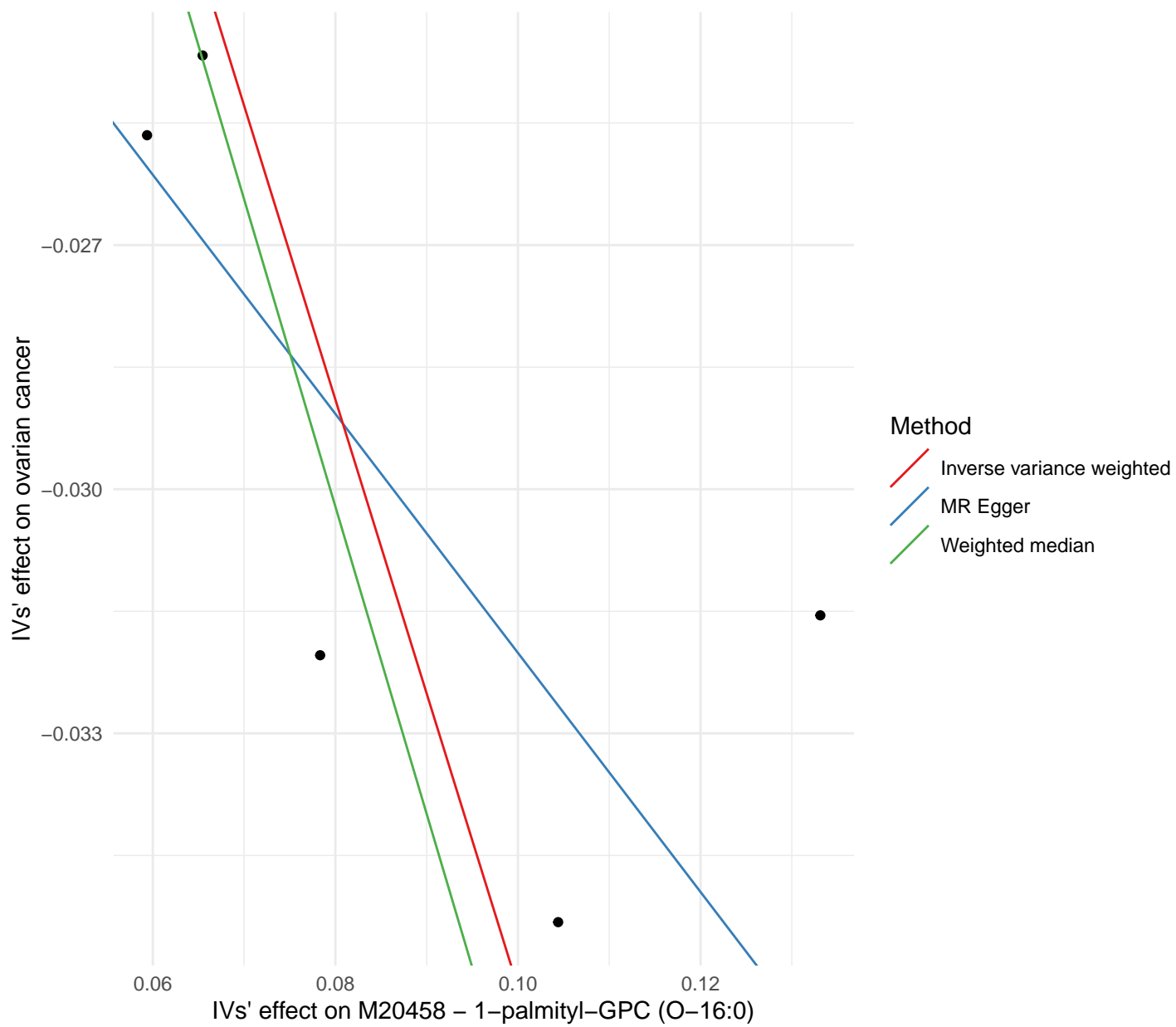

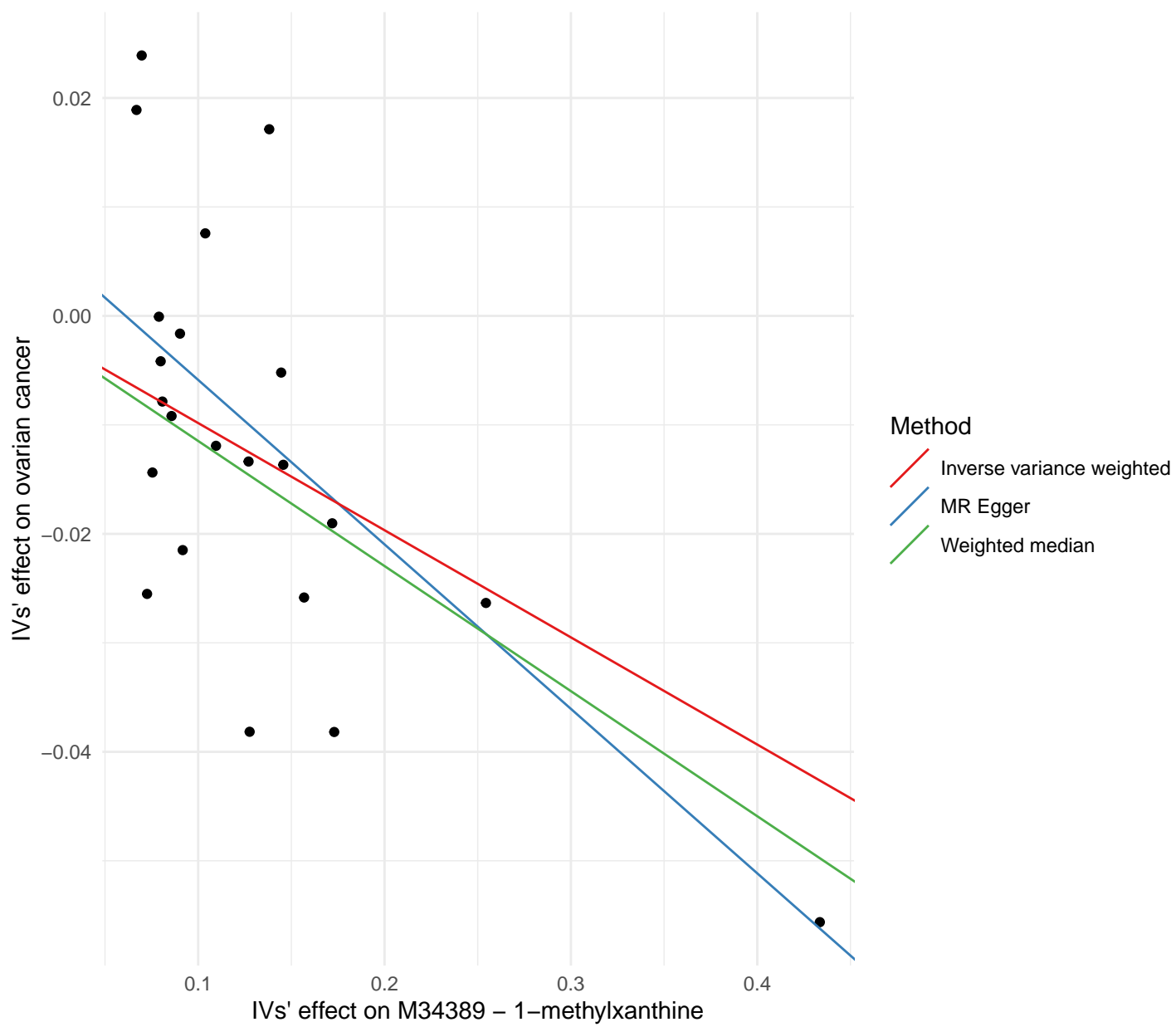

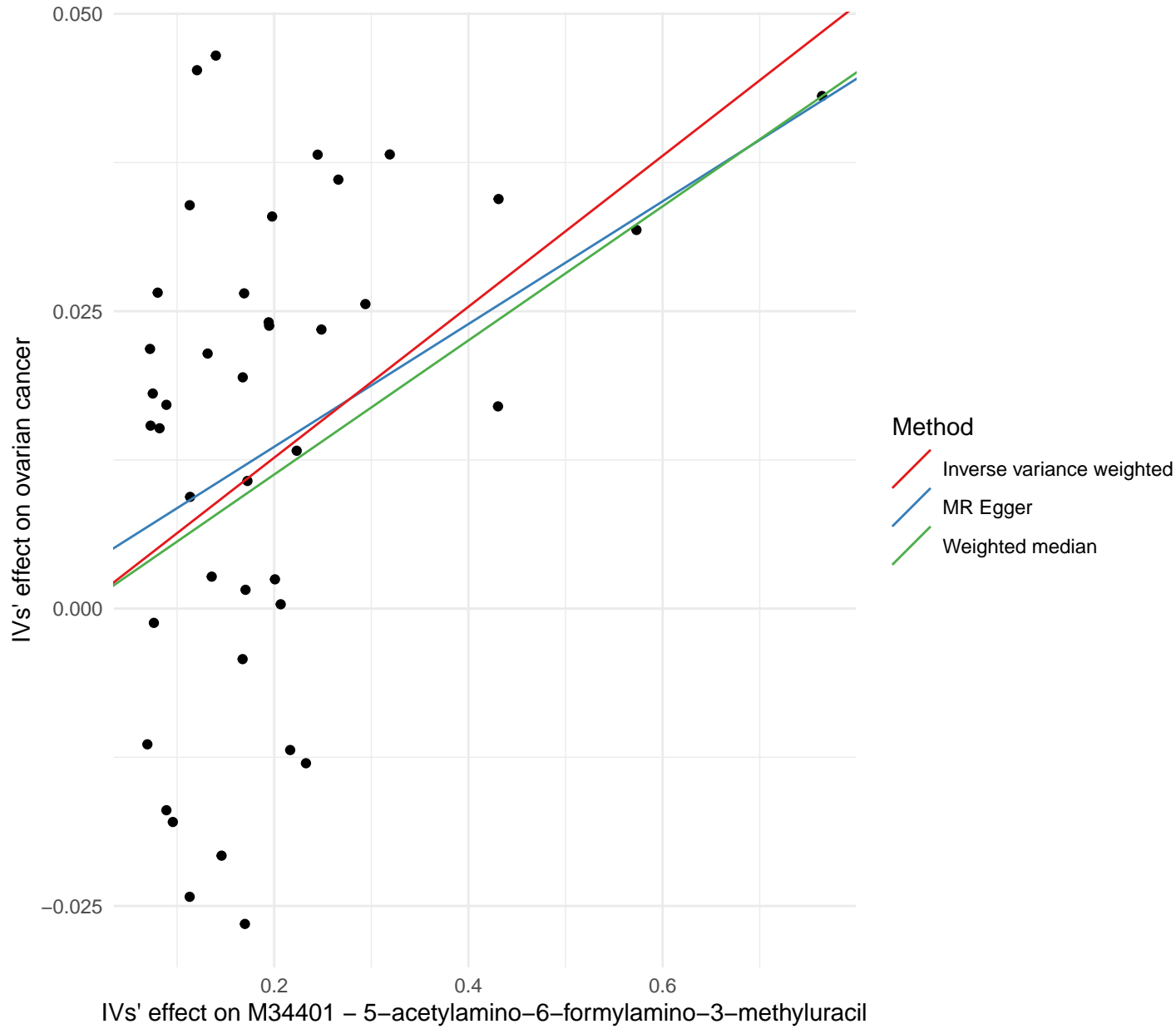

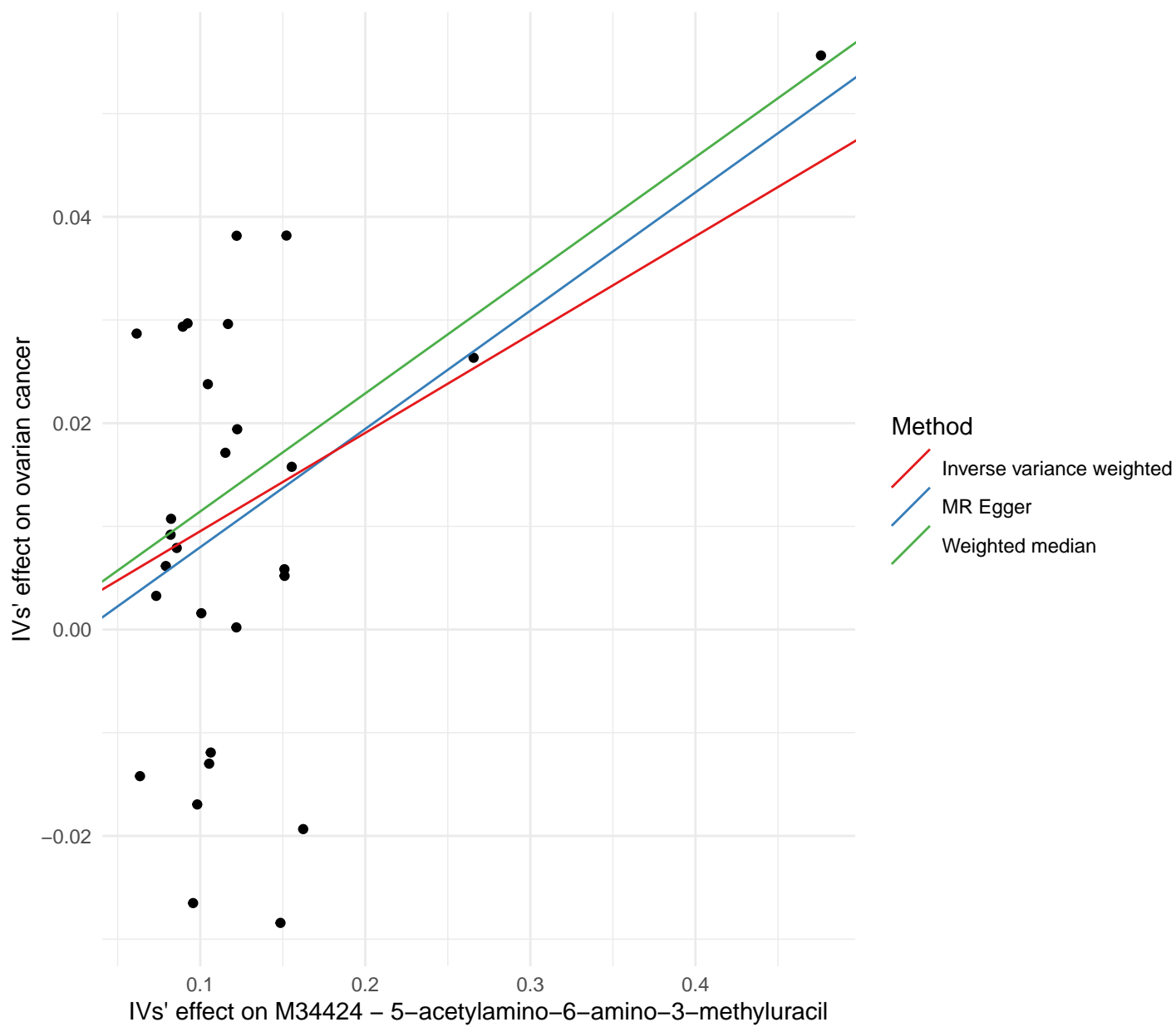

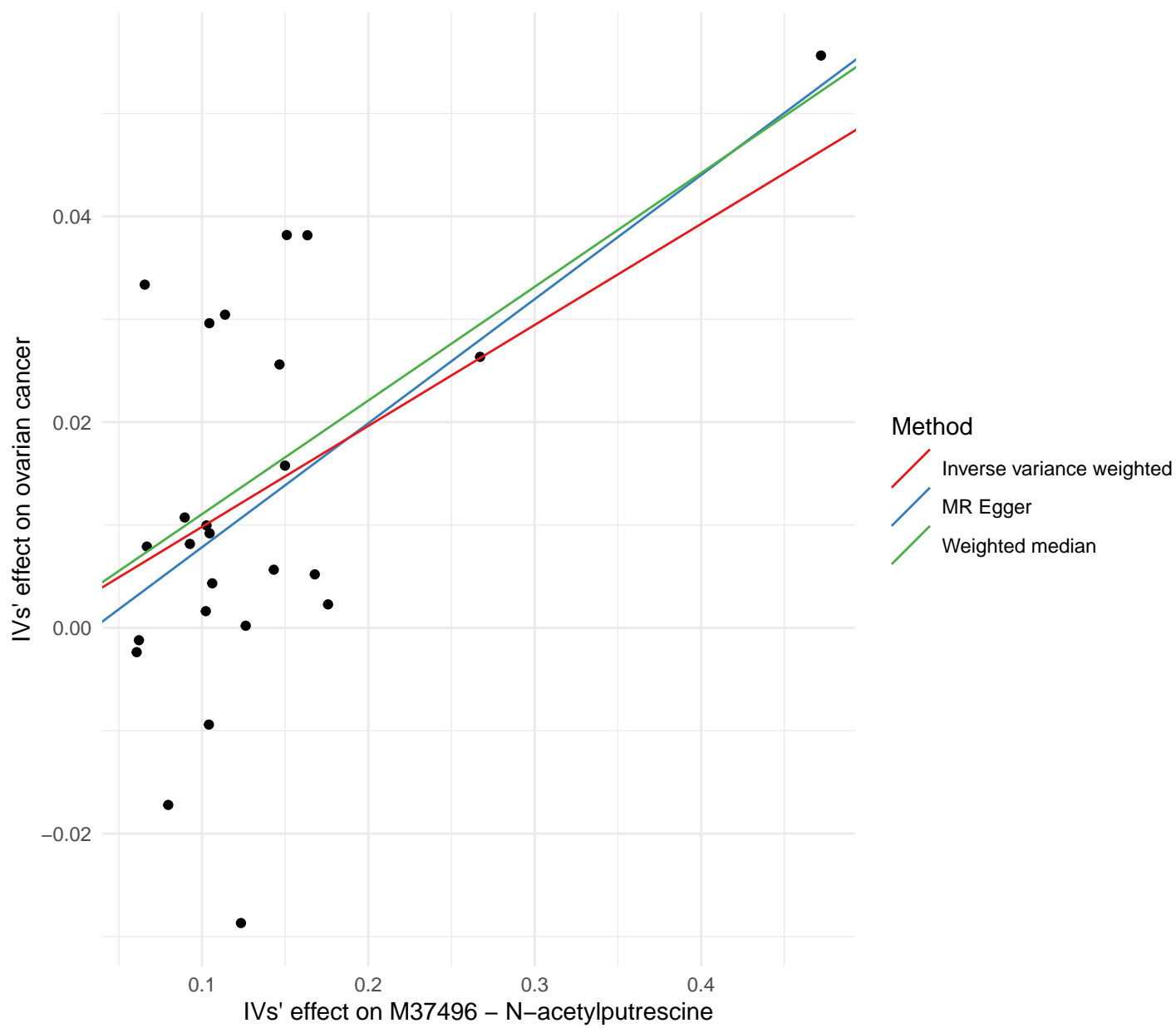

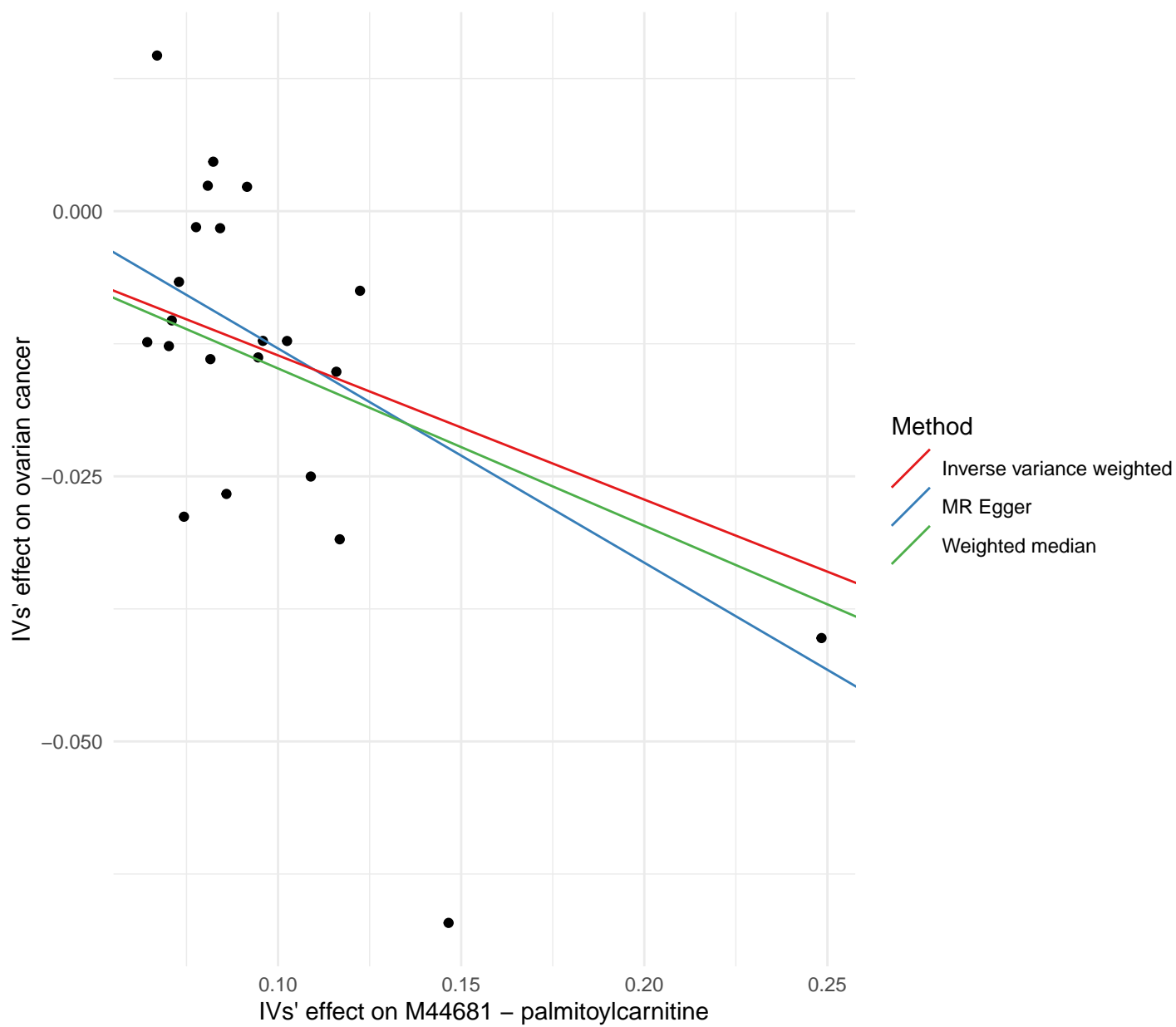

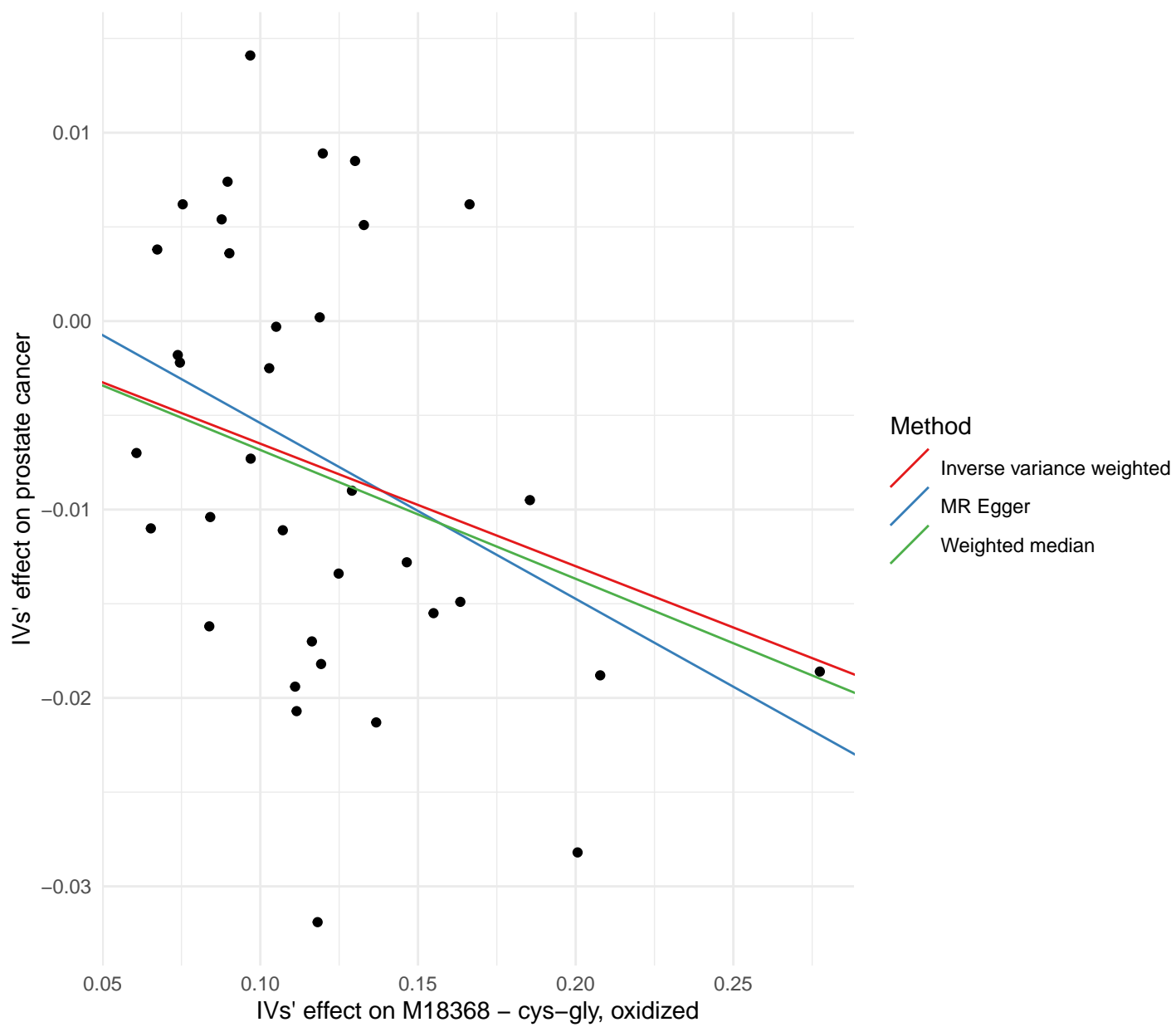

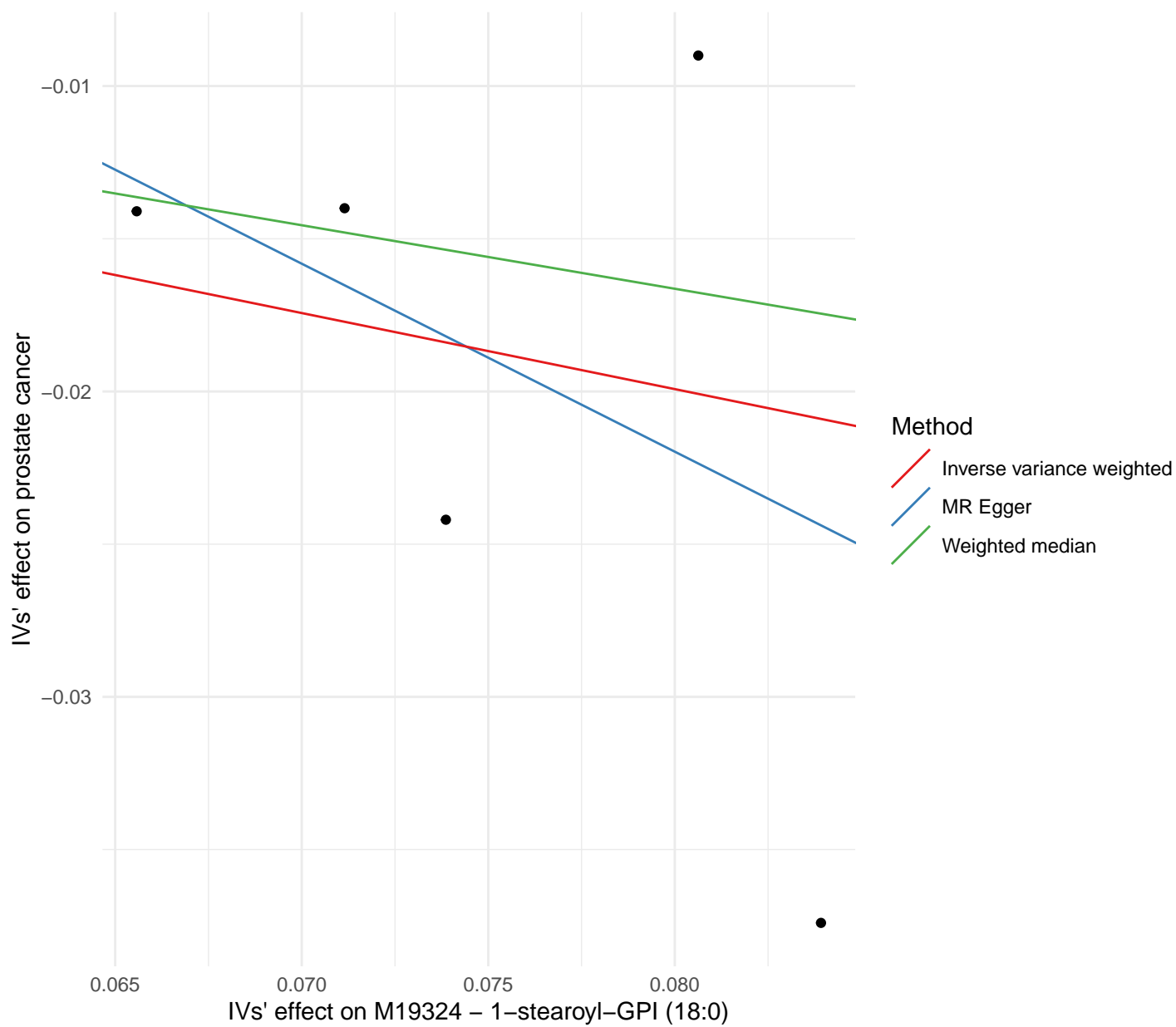

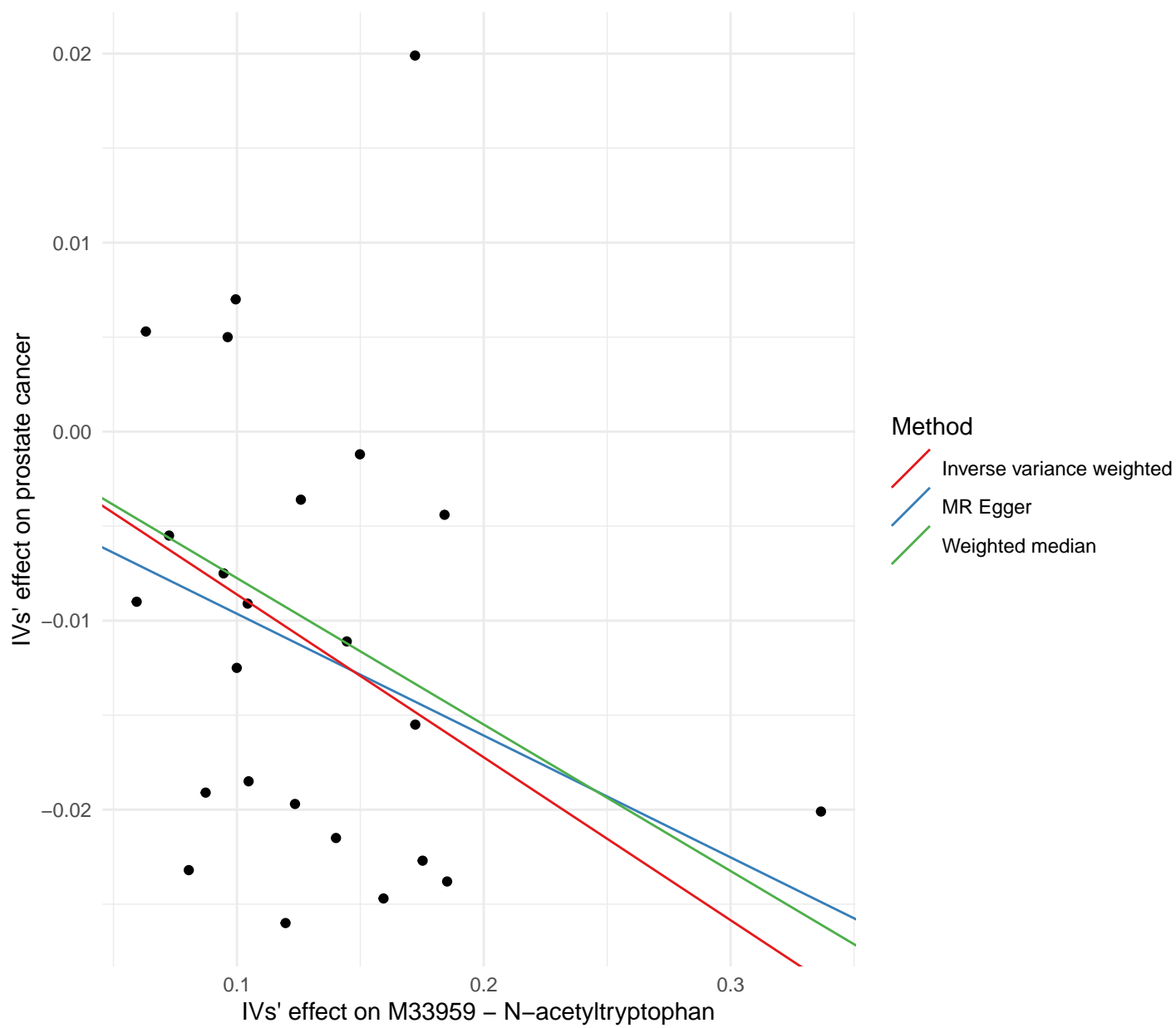

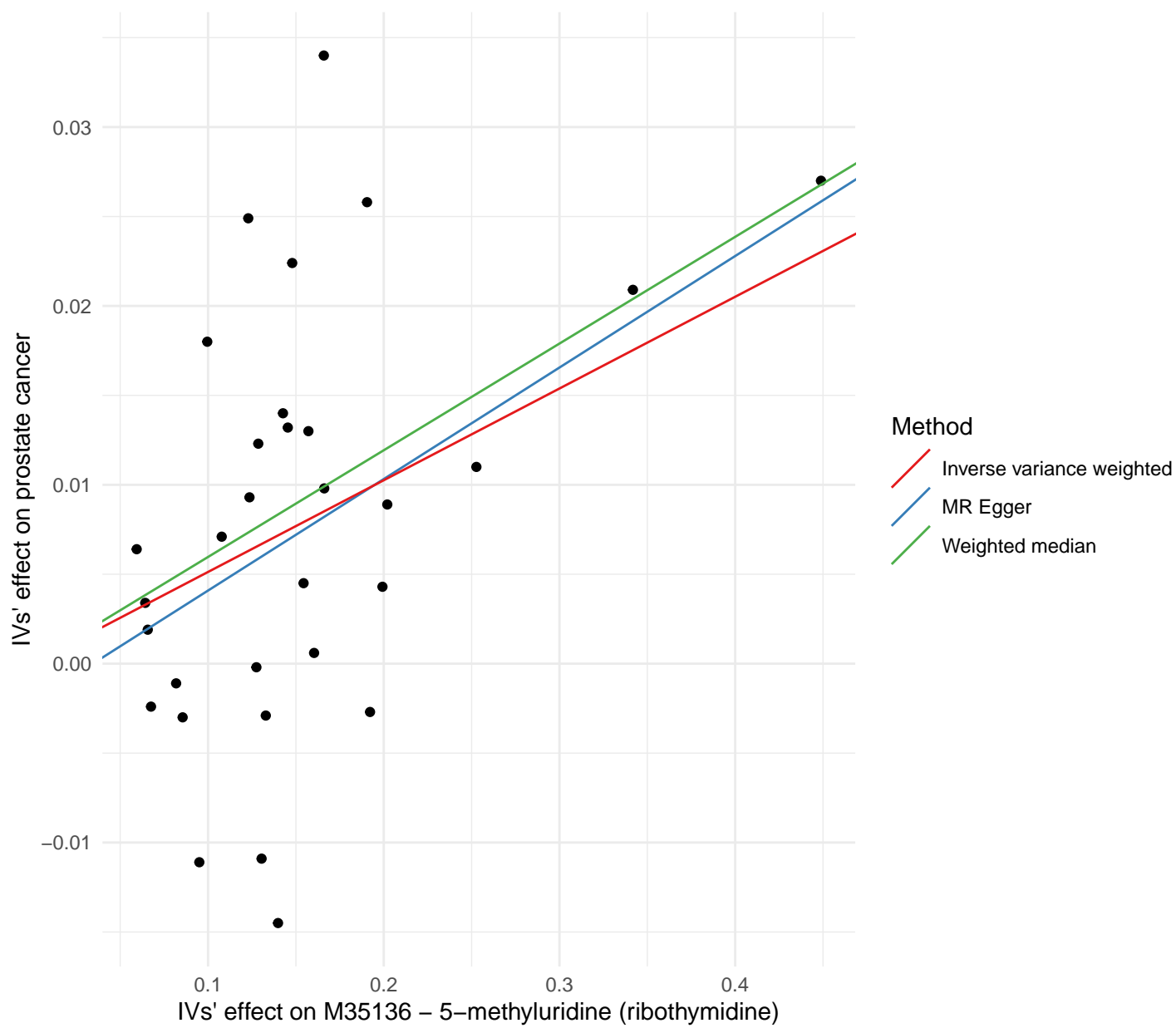

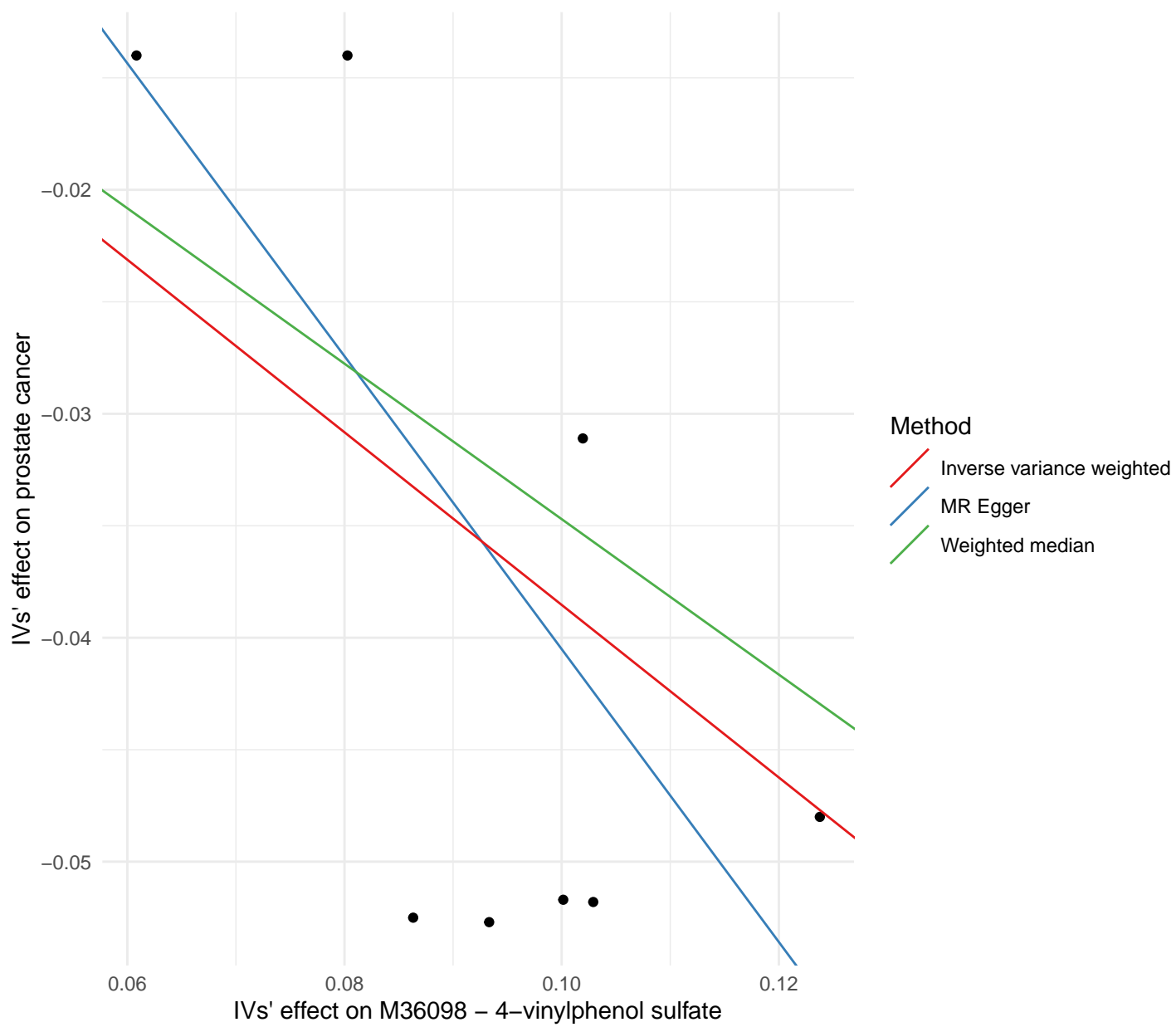

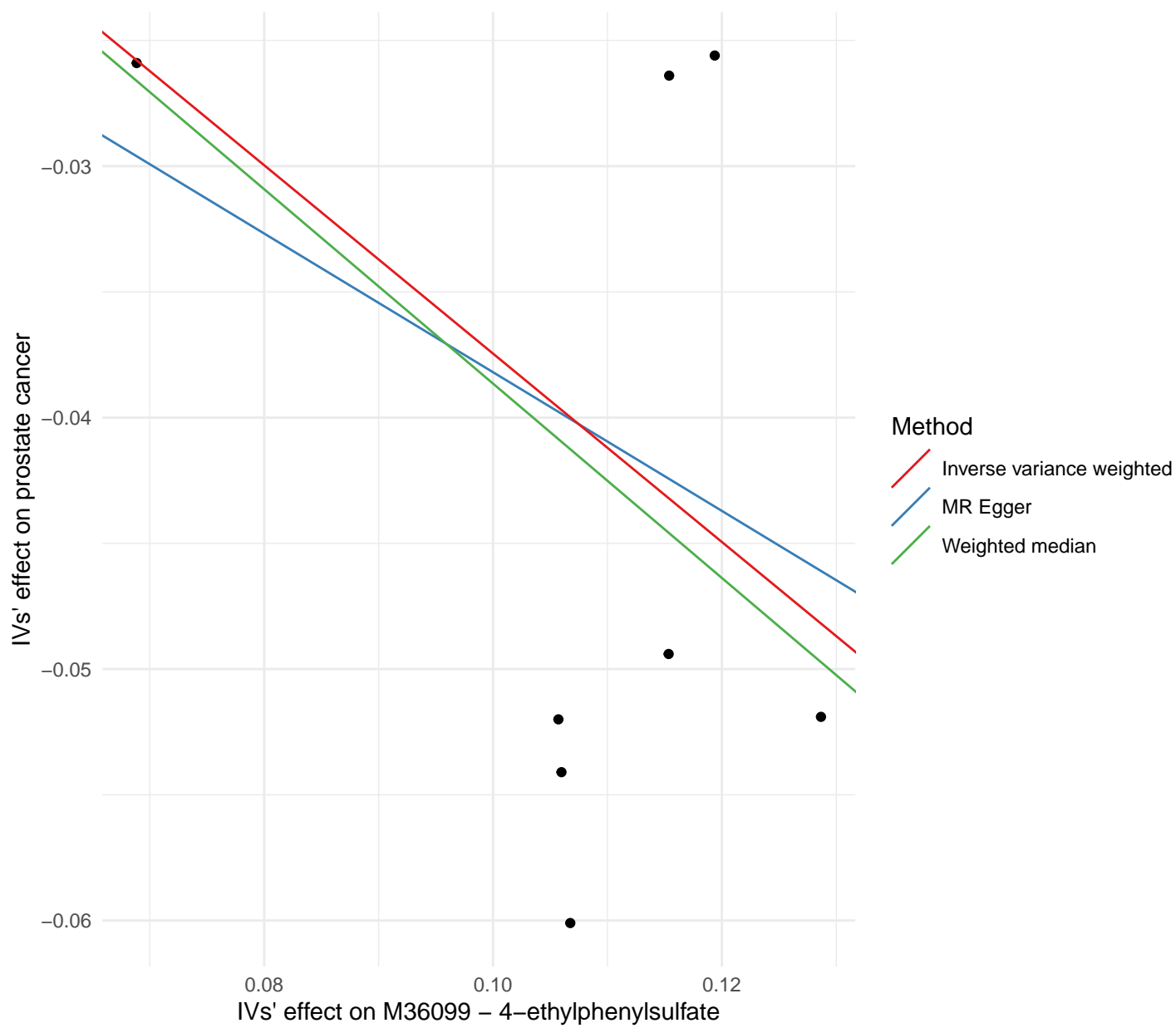

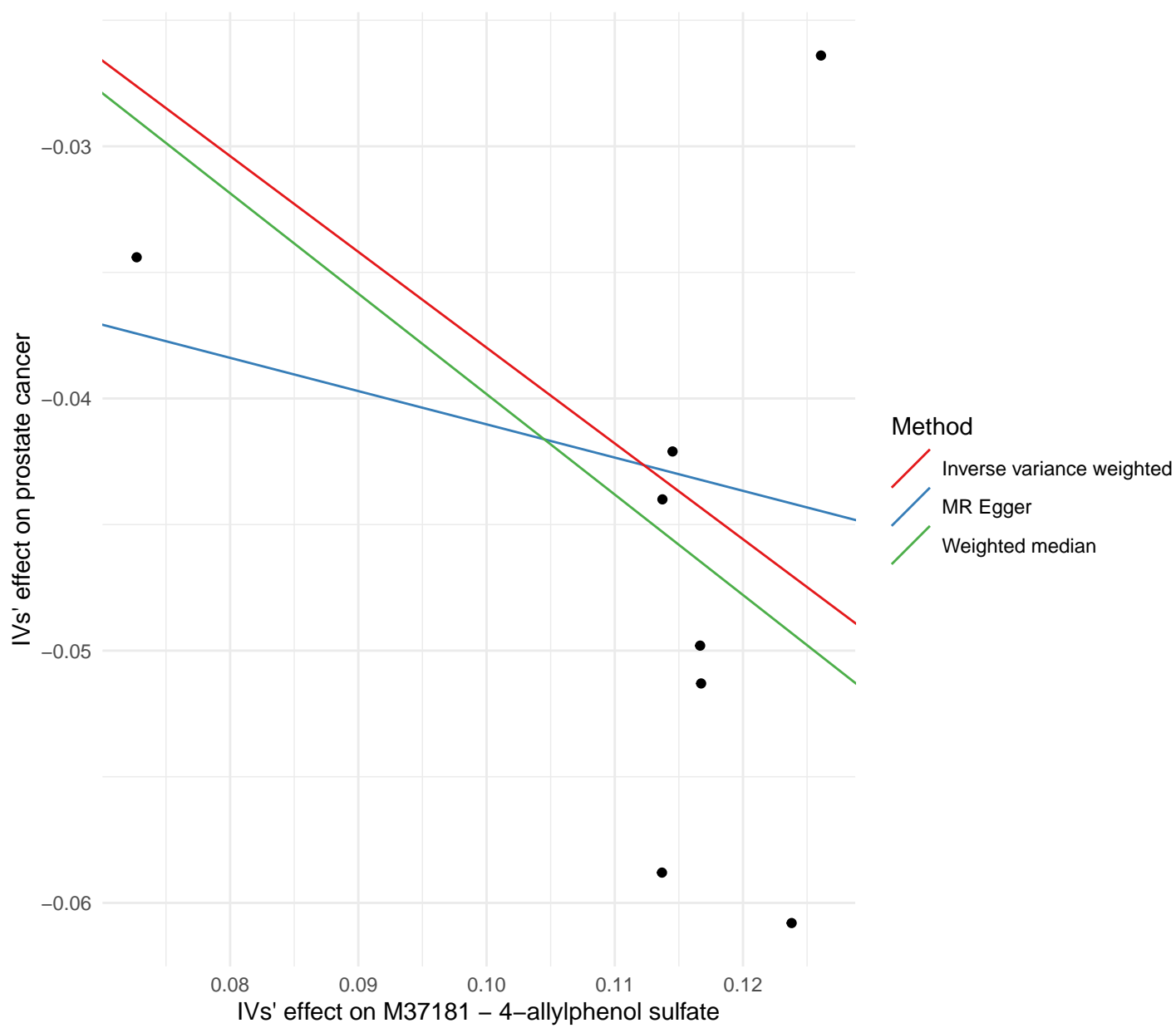

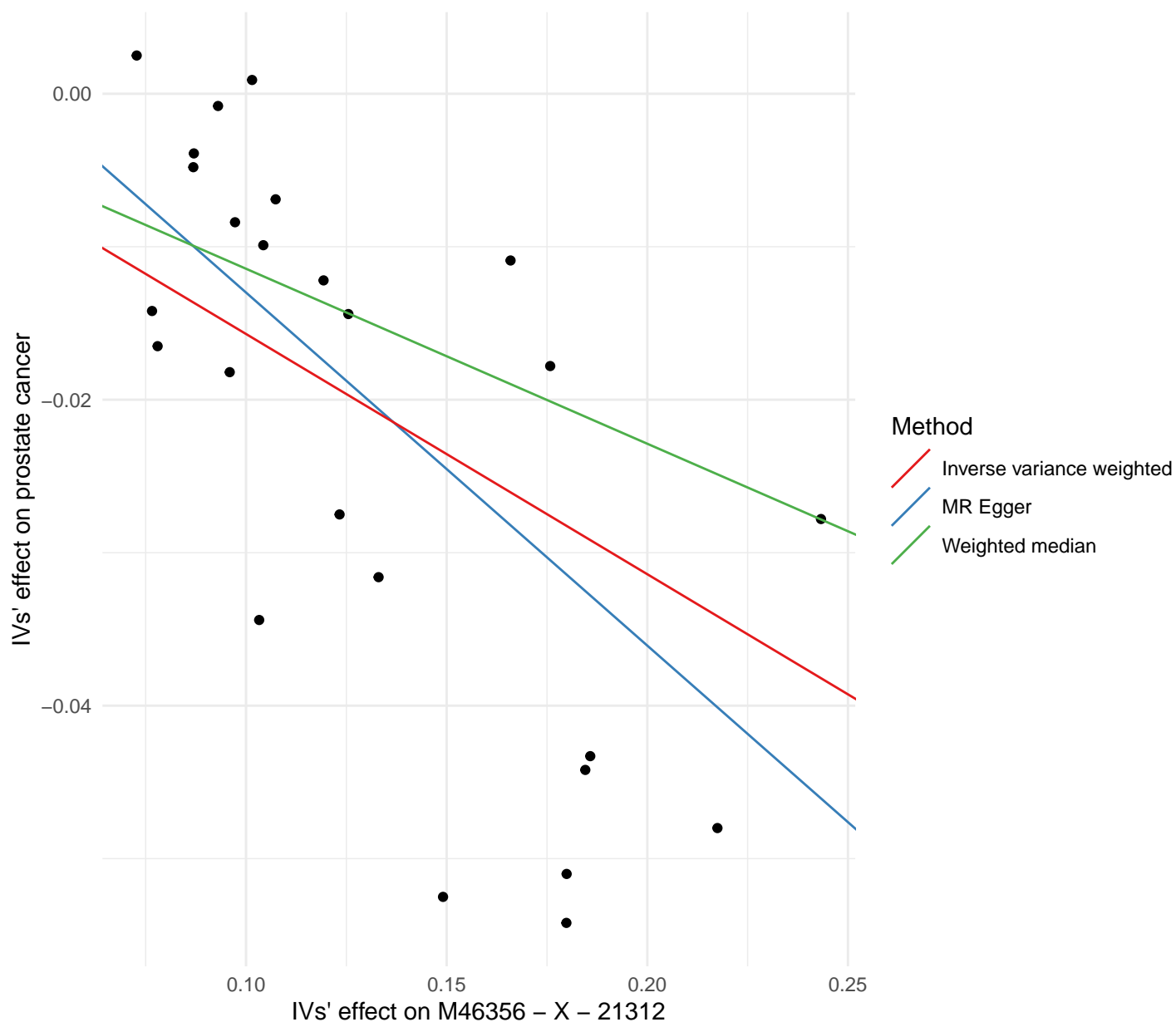

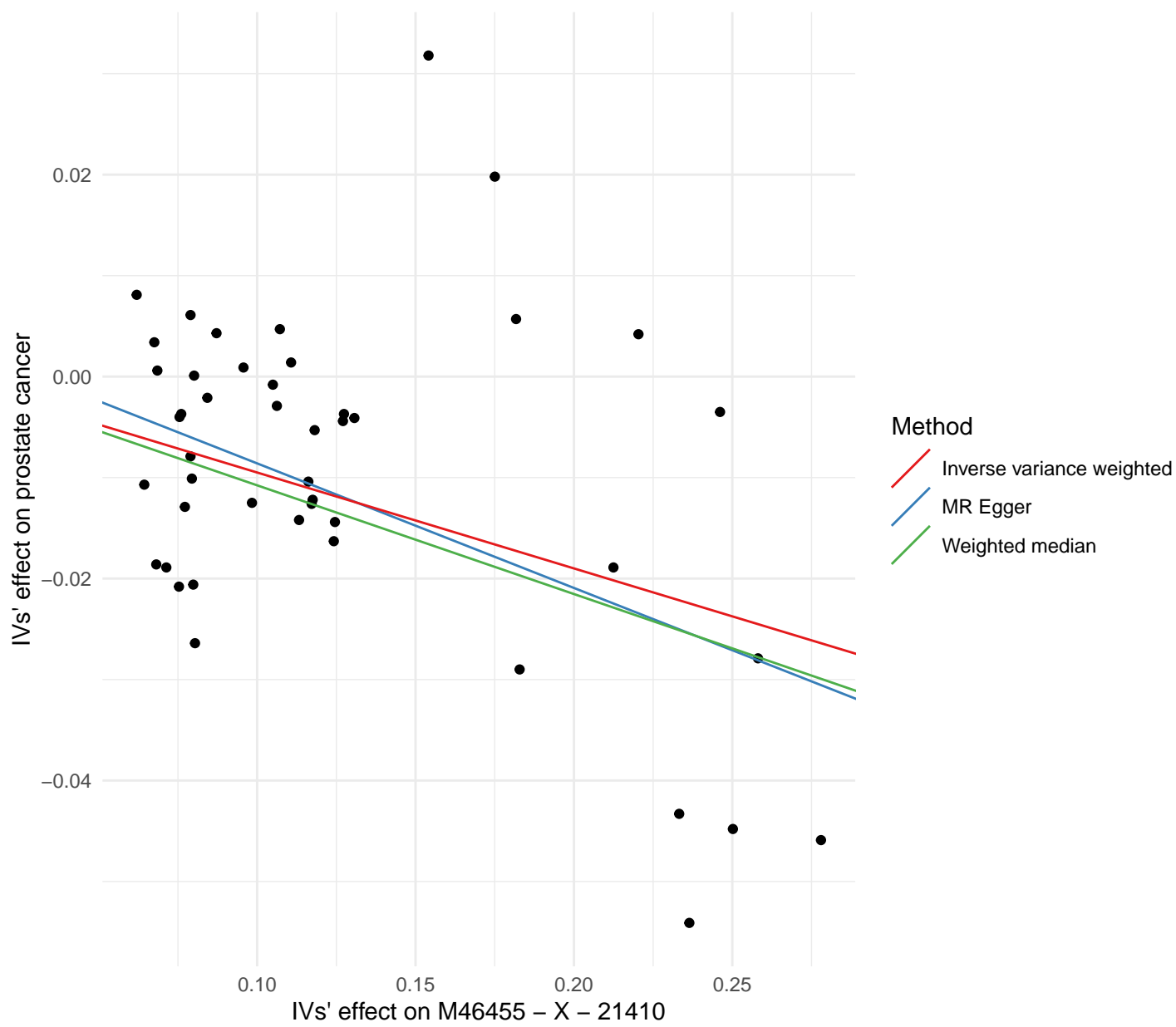

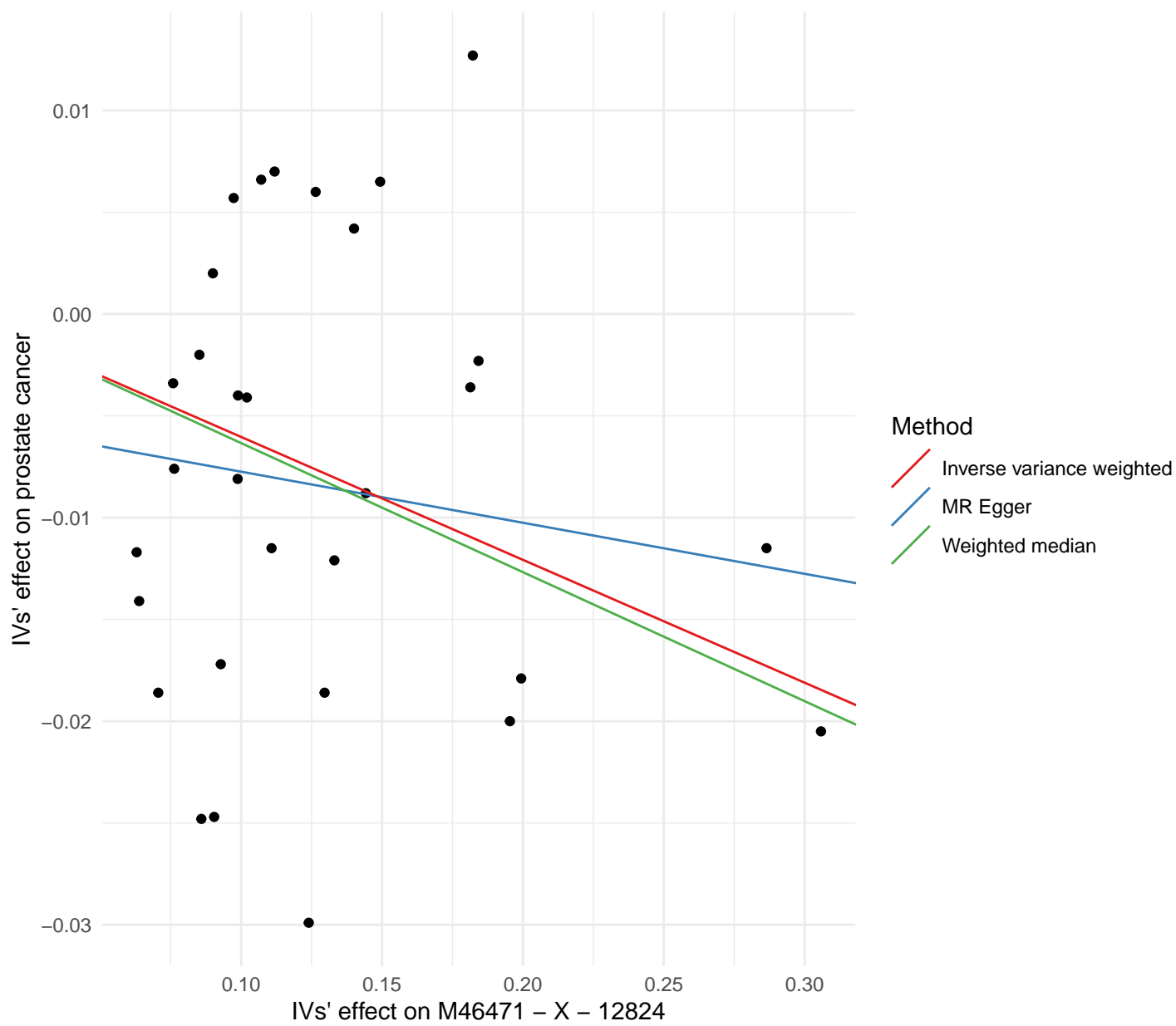

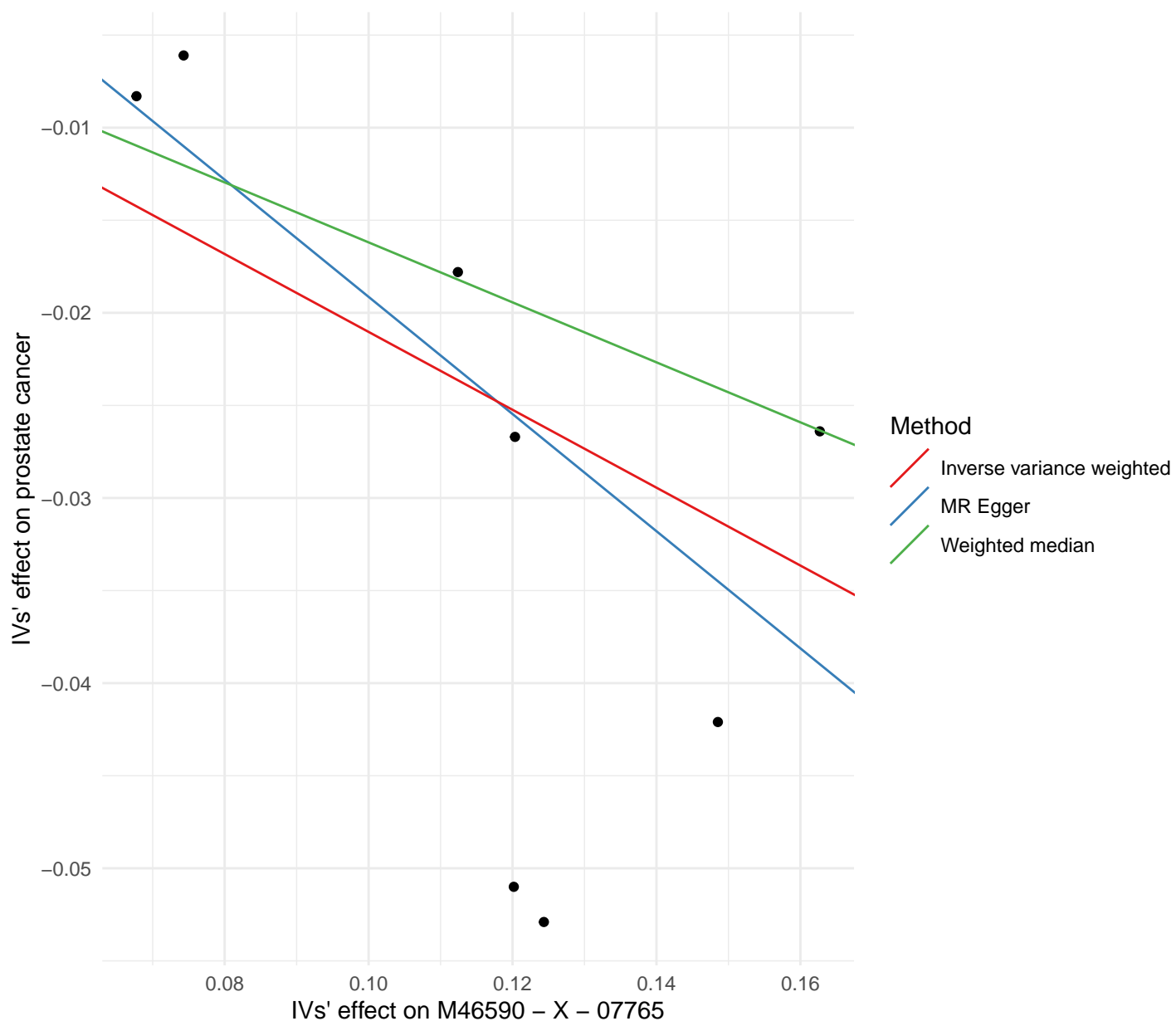

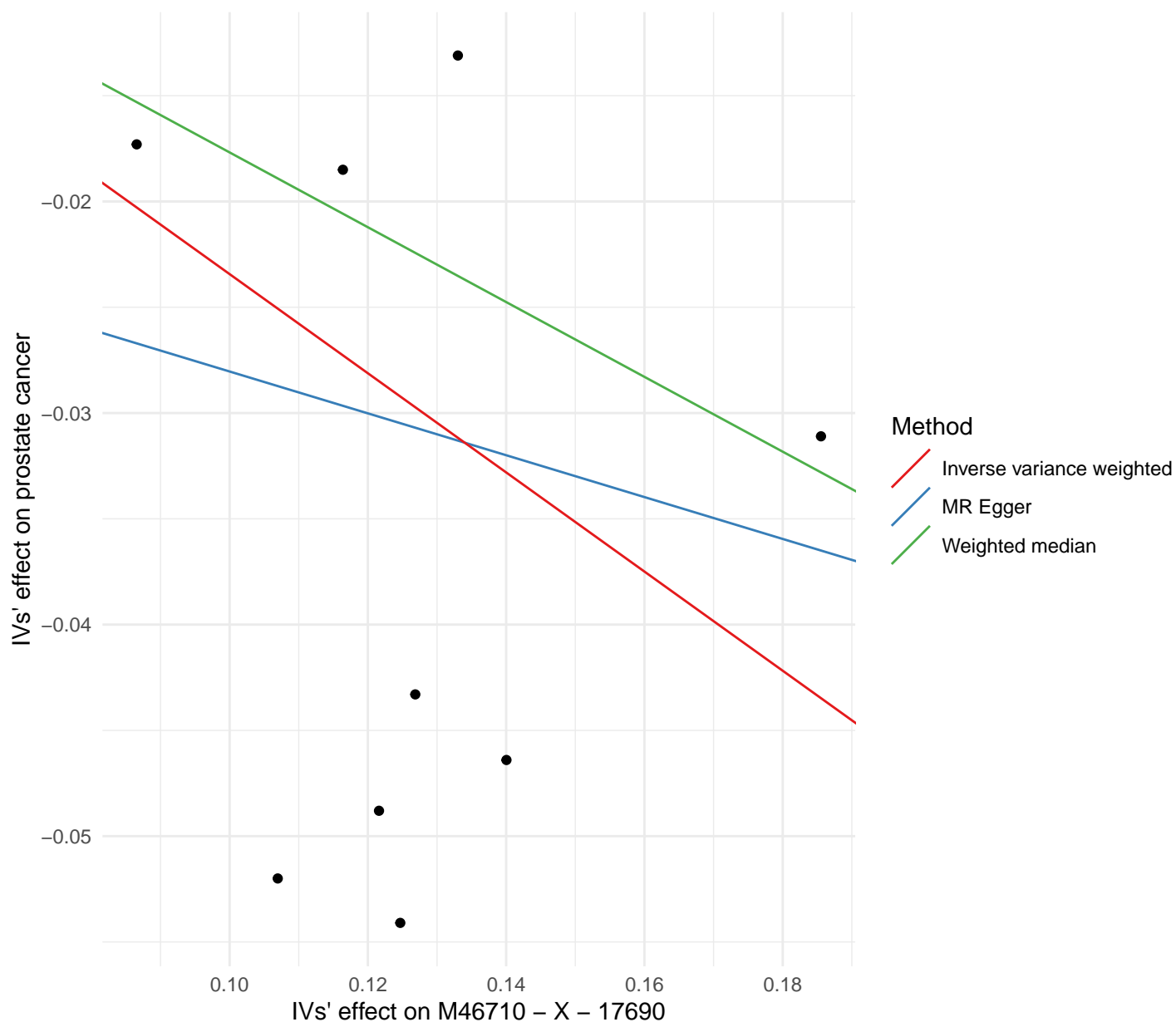

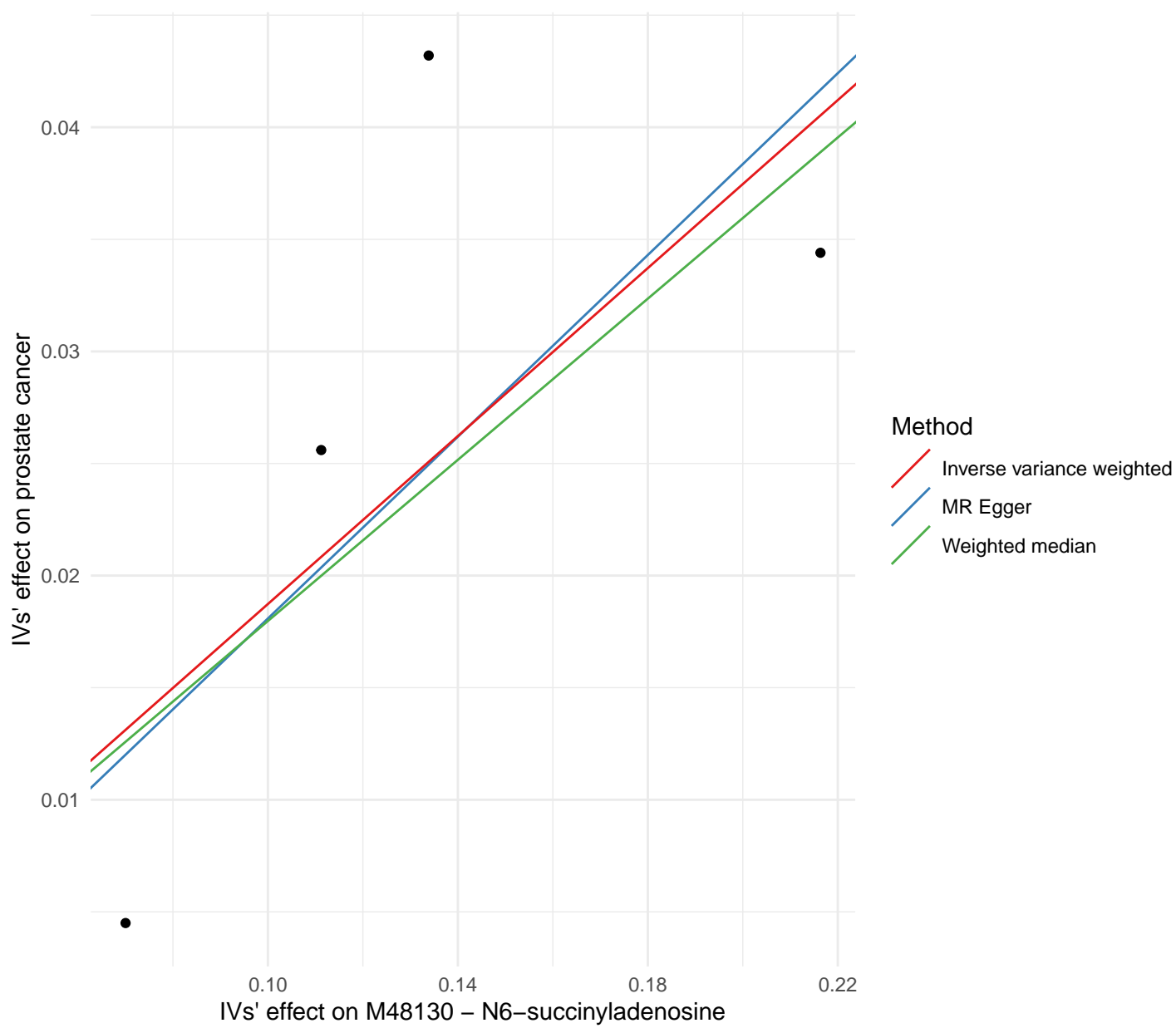

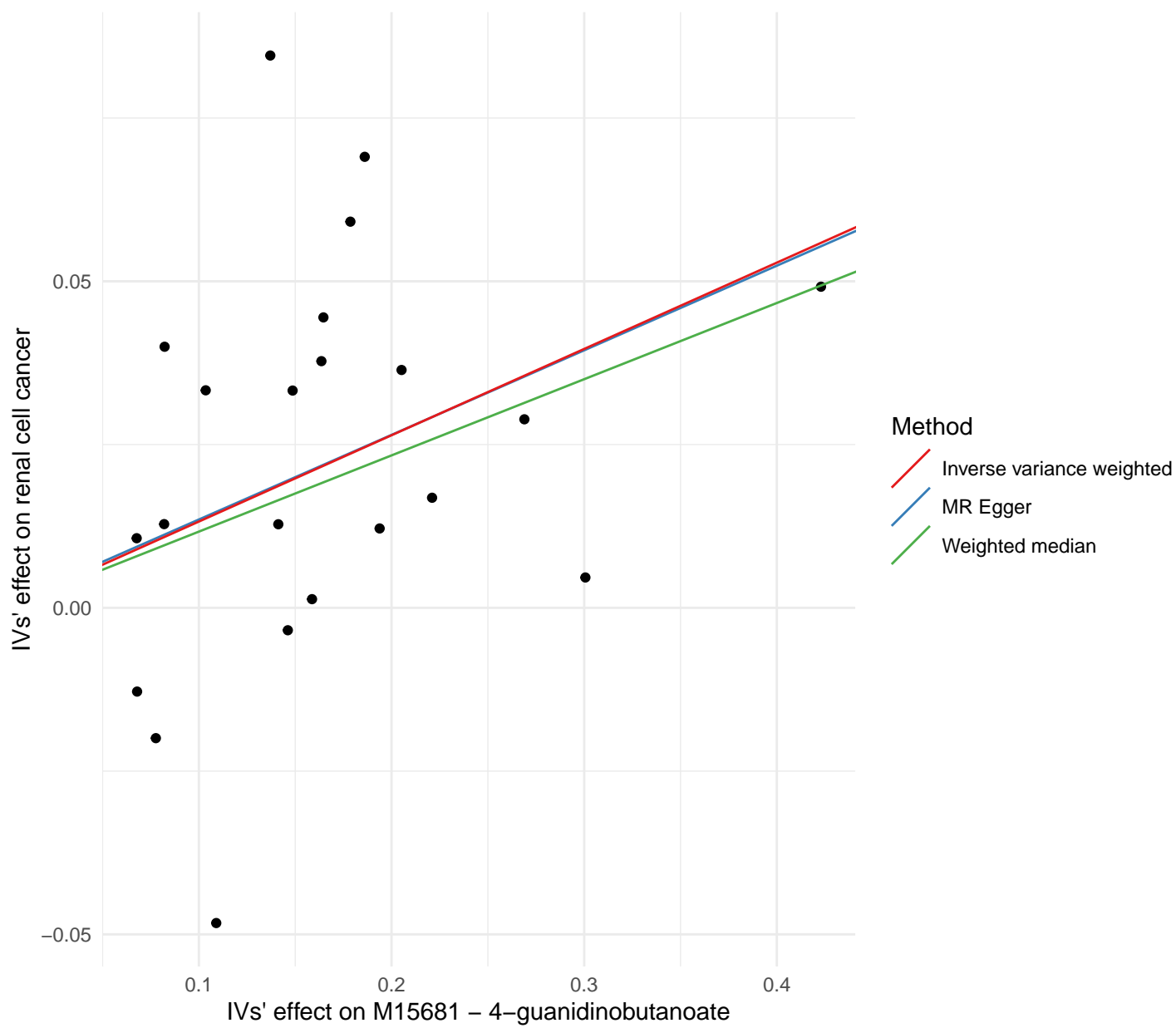

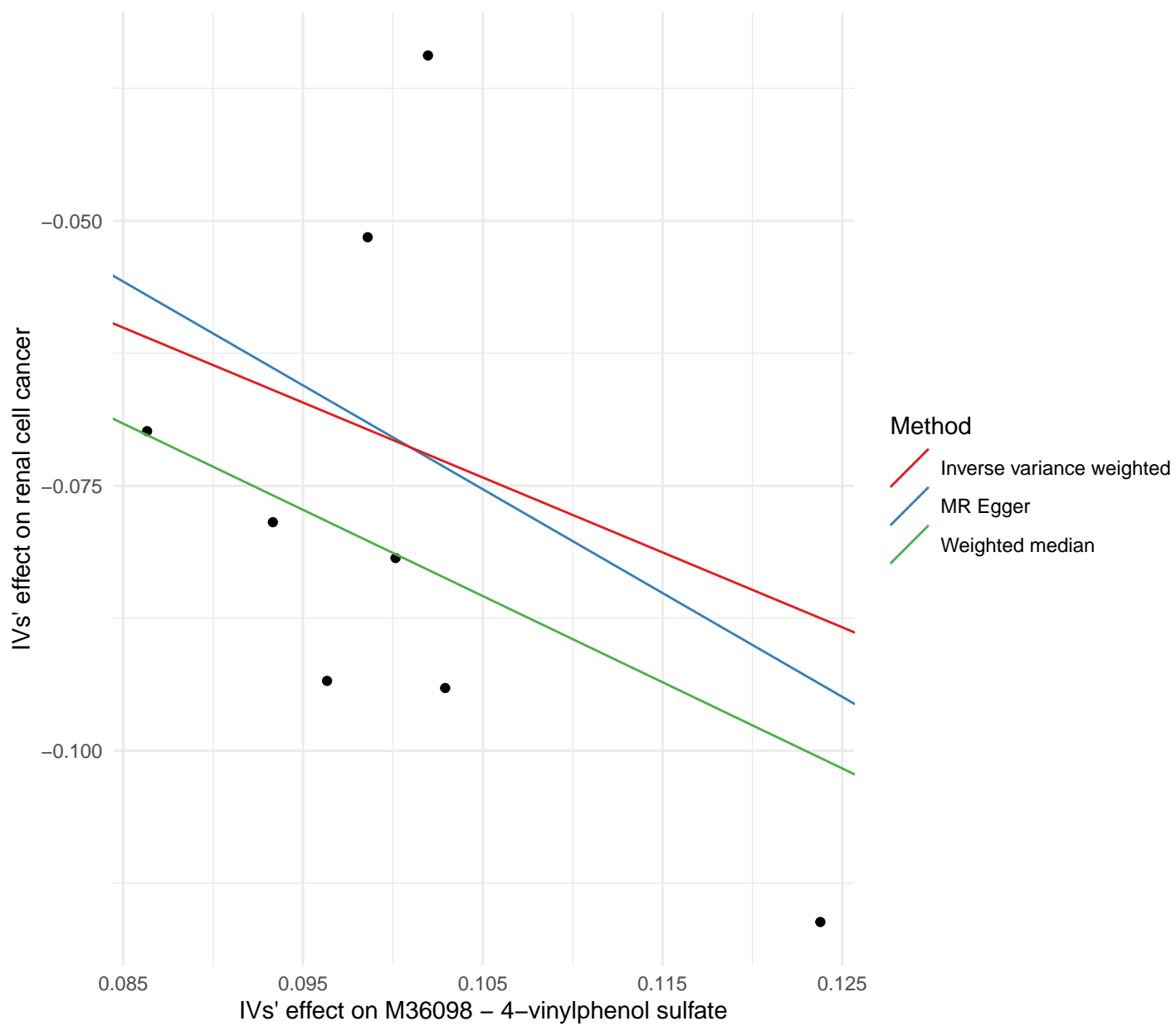

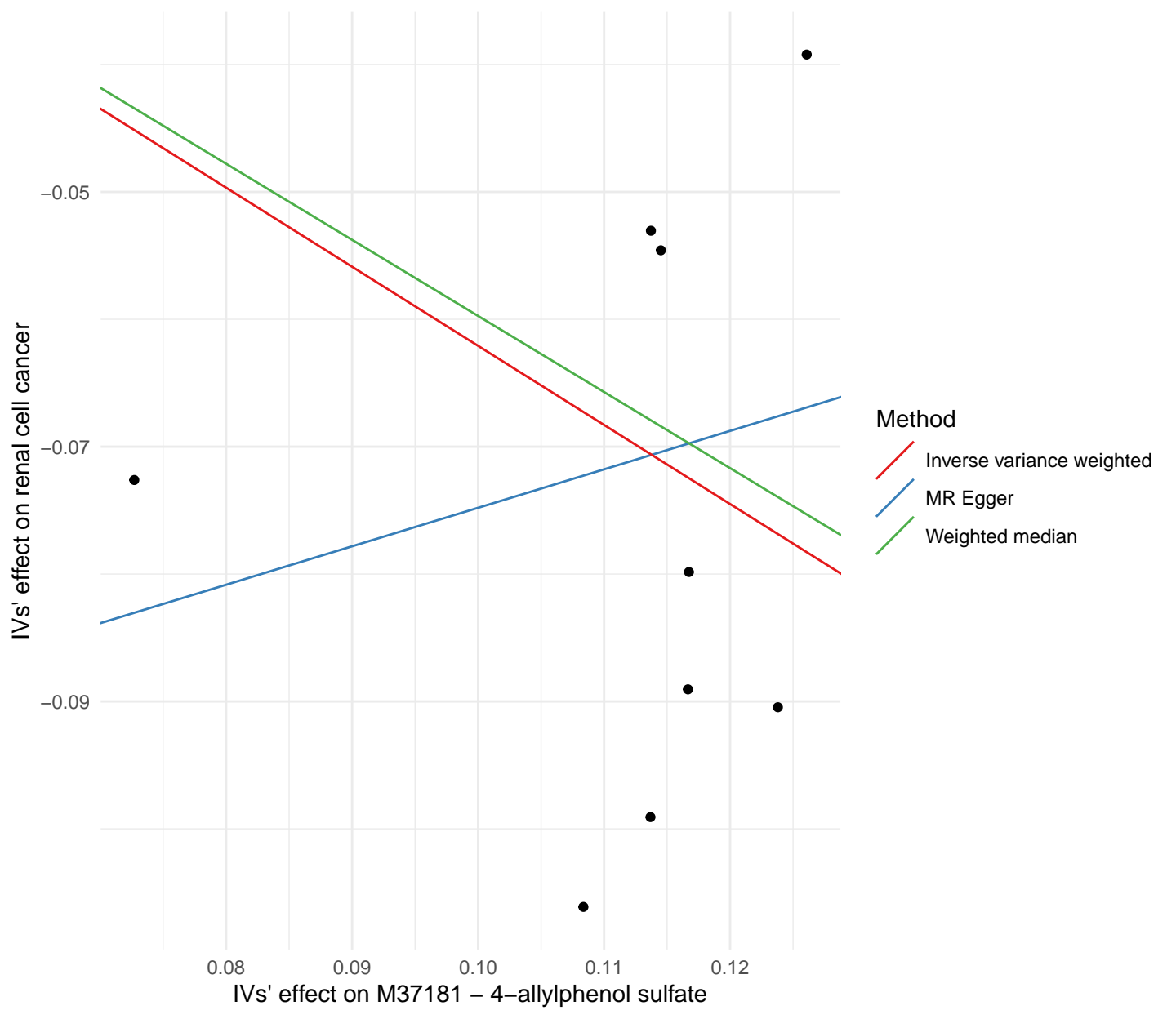

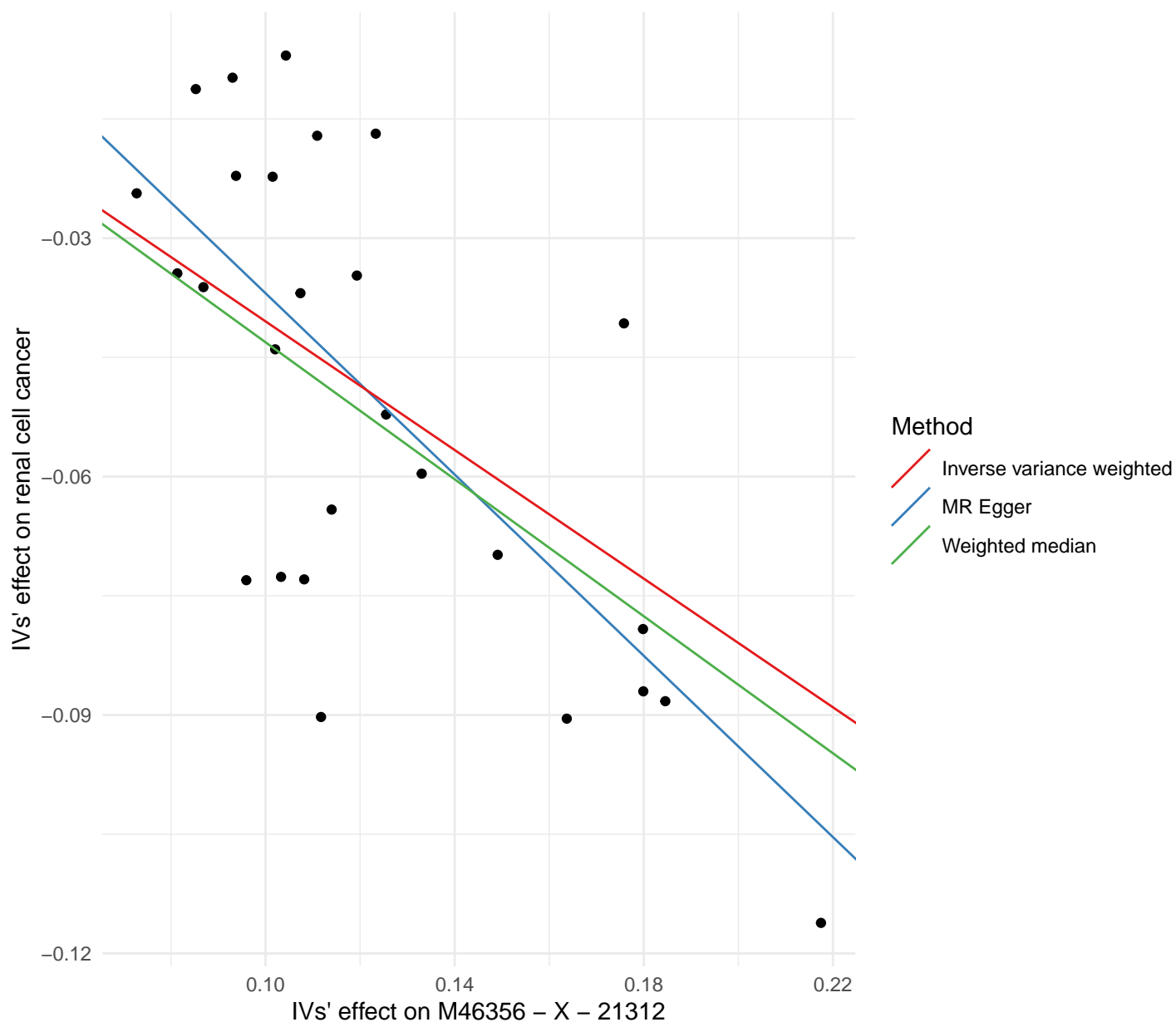

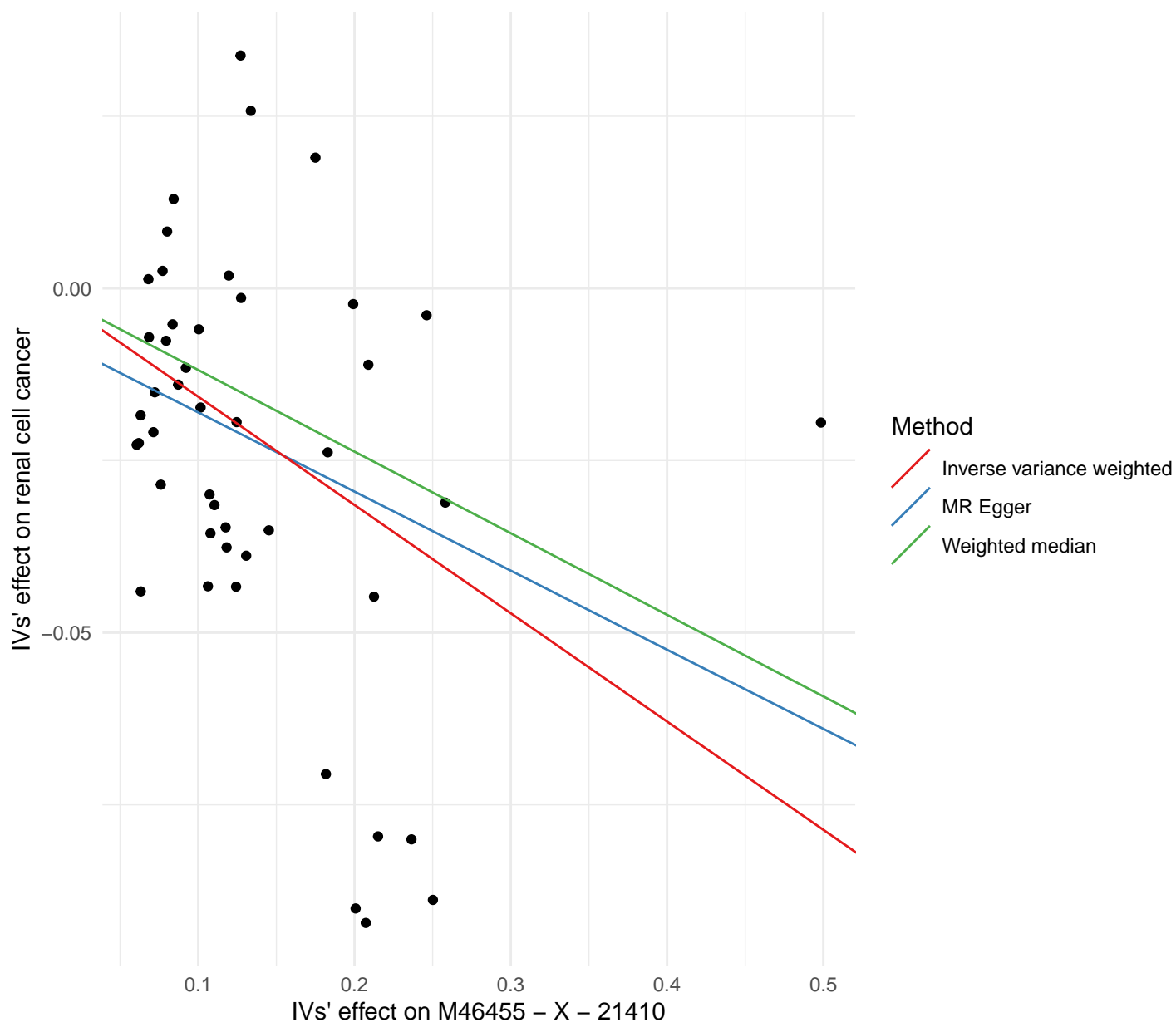

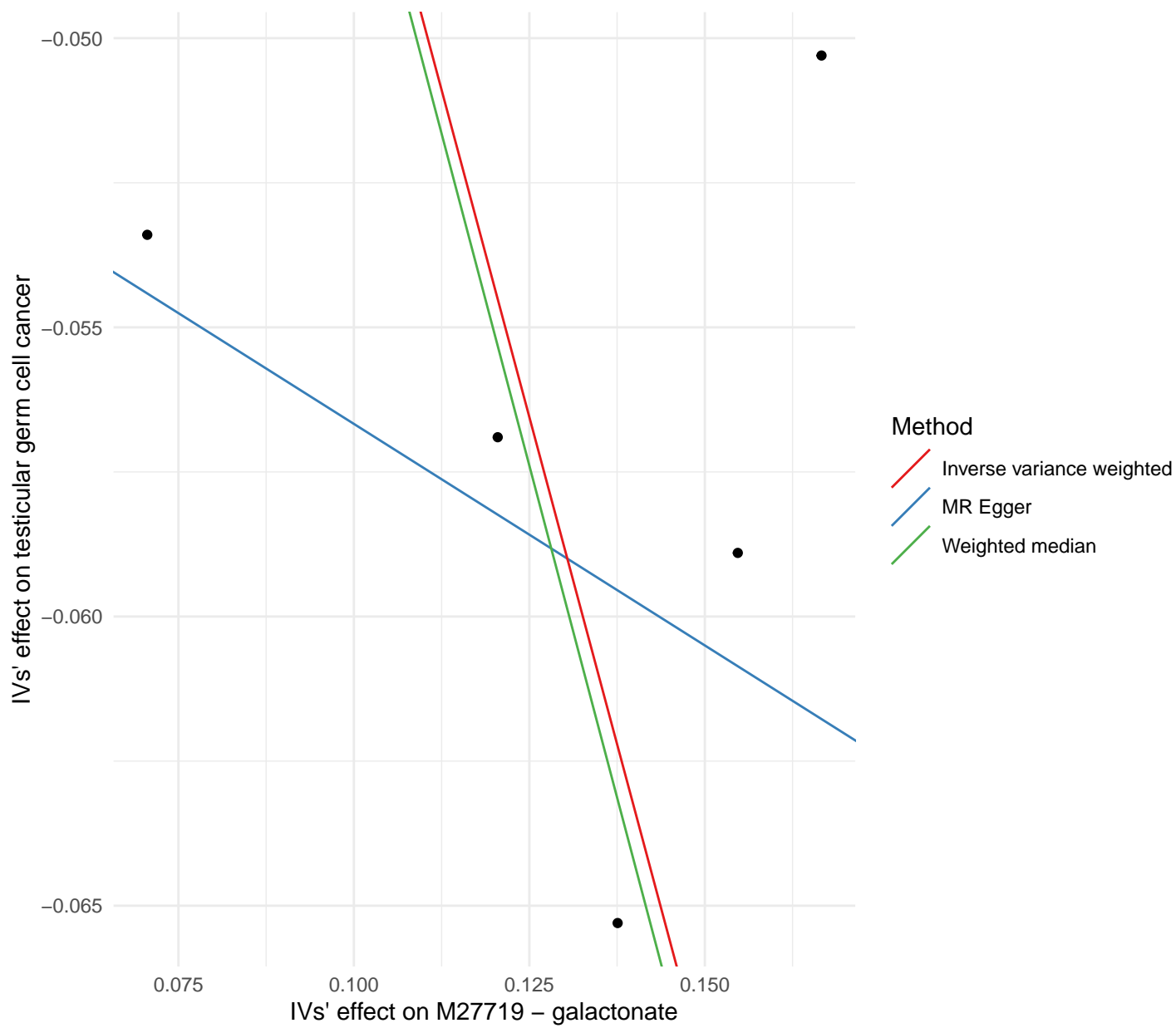

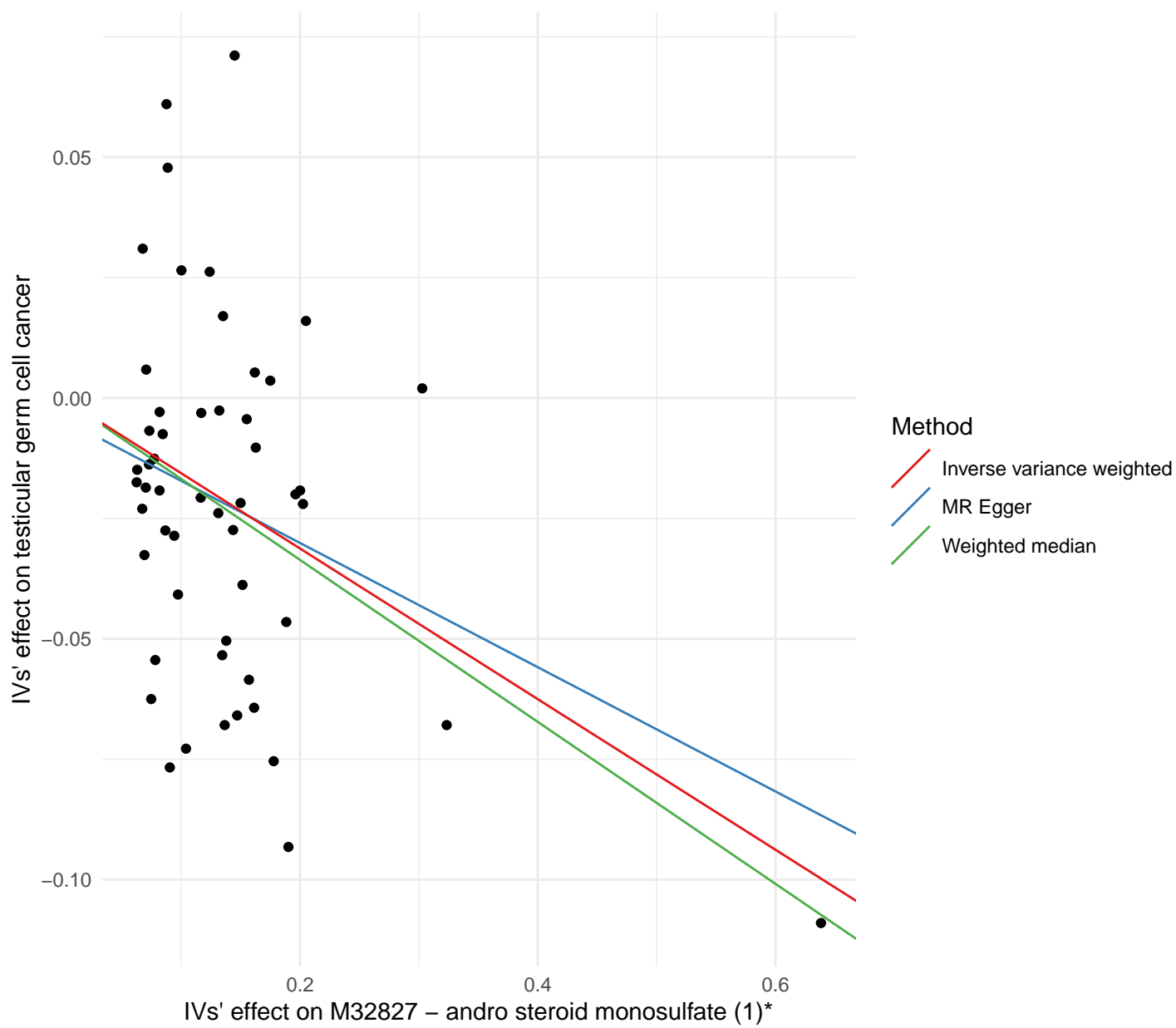

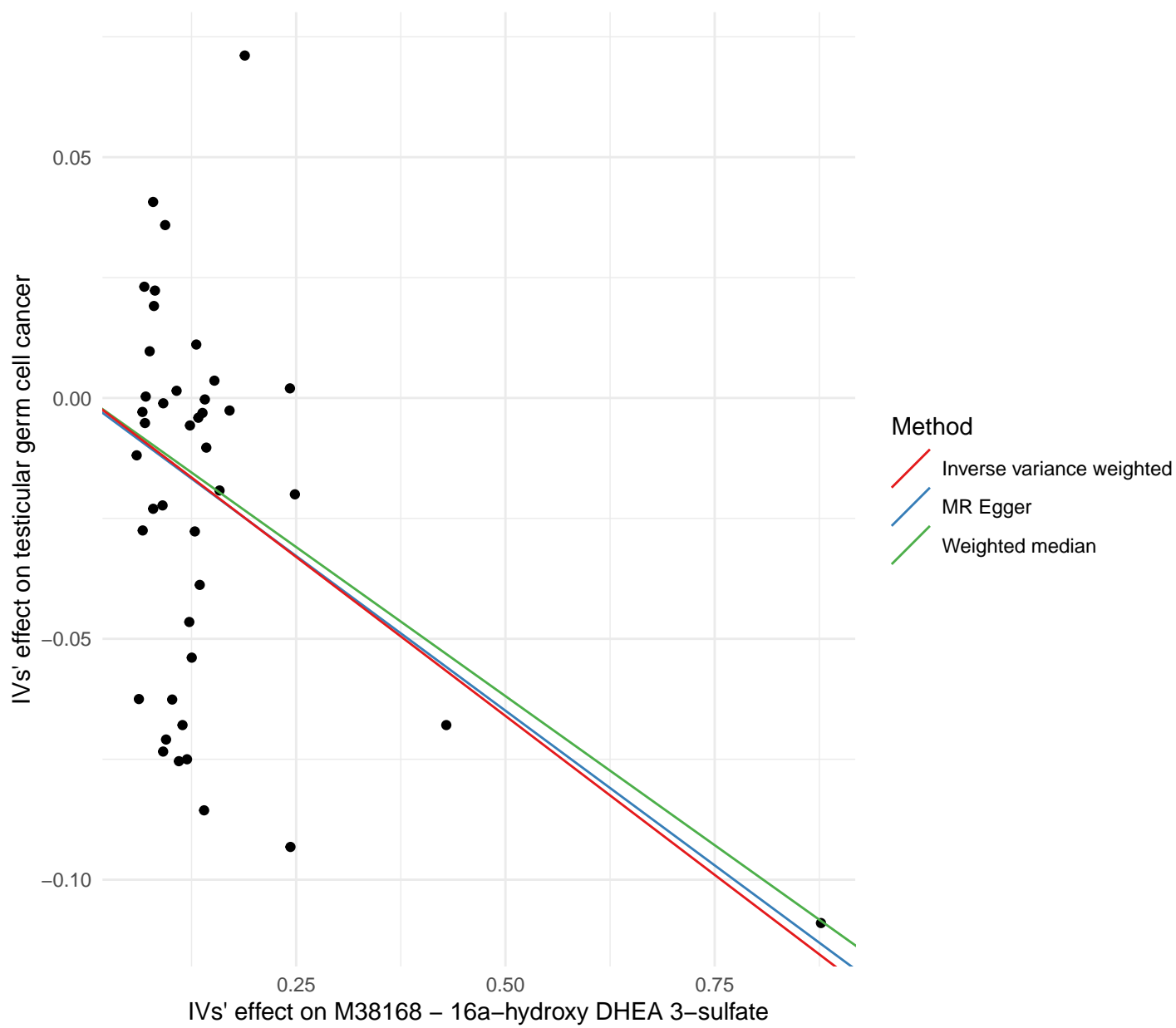

Supplement: Supplementary file 2 — Additional file 2: Fig. S1. Scatter plots of the identified 94 significant metabolite-cancer associations. [file 12916_2024_3272_MOESM2_ESM.pdf]
